# Unraveling the role of *Xist* in X chromosome inactivation: insights from rabbit model and deletion analysis of exons and repeat A

**DOI:** 10.1007/s00018-024-05151-0

**Published:** 2024-03-29

**Authors:** Mingming Liang, Lichao Zhang, Liangxue Lai, Zhanjun Li

**Affiliations:** 1 State Key Laboratory for Diagnosis and Treatment of Severe Zoonotic Infectious Diseases, Key Laboratory for Zoonosis Research of the Ministry of Education, Institute of Zoonosis, and College of Veterinary Medicine, Jilin University, Changchun, 130062 China; 2 CAS Key Laboratory of Regenerative Biology, Guangdong Provincial Key Laboratory of Stem Cell and Regenerative Medicine, Guangzhou Institutes of Biomedicine and Health, Chinese Academy of Sciences, Guangzhou, 510530 China; 3 Institute of Stem Cells and Regeneration, Chinese Academy of Sciences, Beijing, 100039 China; 4 Research Unit of Generation of Large Animal Disease Models, Chinese Academy of Medical Sciences, Guangzhou, 510530 China

**Keywords:** X chromosome inactivation, *Xist*, Repeat A, Animal model, Rabbits

## Abstract

X chromosome inactivation (XCI) is a process that equalizes the expression of X-linked genes between males and females. It relies on *Xist*, continuously expressed in somatic cells during XCI maintenance. However, how *Xist* impacts XCI maintenance and its functional motifs remain unclear. In this study, we conducted a comprehensive analysis of *Xist*, using rabbits as an ideal non-primate model. Homozygous knockout of exon 1, exon 6, and repeat A in female rabbits resulted in embryonic lethality. However, X^∆ReA^X females, with intact X chromosome expressing *Xist*, showed no abnormalities. Interestingly, there were no significant differences between females with homozygous knockout of exons 2–5 and wild-type rabbits, suggesting that exons 2, 3, 4, and 5 are less important for XCI. These findings provide evolutionary insights into *Xist* function.

**Supplementary Information:**

The online version contains supplementary material available at 10.1007/s00018-024-05151-0.

## Introduction

X chromosome inactivation (XCI) is a dosage compensation mechanism that evolved in marsupial and placental mammals to equalize the expression of X-linked genes between females (XX) and males (XY) [1–5]. The initiation of XCI is genetically controlled by a master regulatory locus called the X-inactivation center (Xic) [6–10]. In mice and humans, dosage compensation is mediated by a long noncoding RNA (lncRNA) called *Xist*. *Xist* is up-regulated from one of the two X chromosomes and its RNA accumulates over the inactive X chromosome (Xi) to trigger gene silencing [7, 9, 11]. Once established, XCI is stably inherited upon successive cell divisions in female somatic cells. Extensive studies have confirmed that *Xist* is both necessary and sufficient for XCI [12–14].

Since Lyon proposed XCI in 1961 [1], mouse models have been widely used to study the molecular mechanisms of X inactivation [5–7, 15–18]. In mice, the *Xist* gene consists of seven exons, interspersed with repetitive sequences called repeats A to F [19]. These exons and repetitive sequences play important roles in the localization and spreading of *Xist* RNA on the inactive X chromosome (Xi) [20]. Repeat A, in particular, has been extensively studied and found to be essential for *Xist*-mediated gene silencing. It also facilitates the recruitment of chromatin-modifying factors through interactions with specific proteins [21–30]. Other repeats, such as B and C, also contribute to *Xist*-mediated gene silencing and the establishment of the inactive chromatin state [22, 24, 31–35]. Repeat E is crucial for the proper localization of *Xist* RNA on the inactive X chromosome (Xi) and facilitates gene silencing [13, 36–41]. Studies on mouse embryonic stem cells have identified specific exons that are important in XCI. For example, the 5' region of *Xist* plays a critical role in gene silencing [25, 42], while exon 4 has a lesser impact on X inactivation [43]. In human cells, different exons, like exon 5, are vital for maintaining XCI, while exons 2, 3, and 4 are relatively less significant [44].

A significant amount of our current understanding of X chromosome inactivation (XCI) mechanisms comes from studies conducted on mice and in vitro female stem cells [5, 45, 46]. The gene *Xist*, which is involved in XCI, exhibits species-specific differences in its regulation and function. In humans, *XIST* is expressed on both X chromosomes, which undergo random XCI during cell differentiation [47, 48]. In contrast, mice initially exhibit paternal *Xist* expression during X chromosome inactivation, followed by random X chromosome inactivation during subsequent stages. Therefore, there are notable differences between the mechanisms of X inactivation in mice and humans [48]. Consequently, it is crucial to establish suitable animal models for investigating the functions of *XIST* and studying the mechanisms of XCI.

**Fig. 1:**
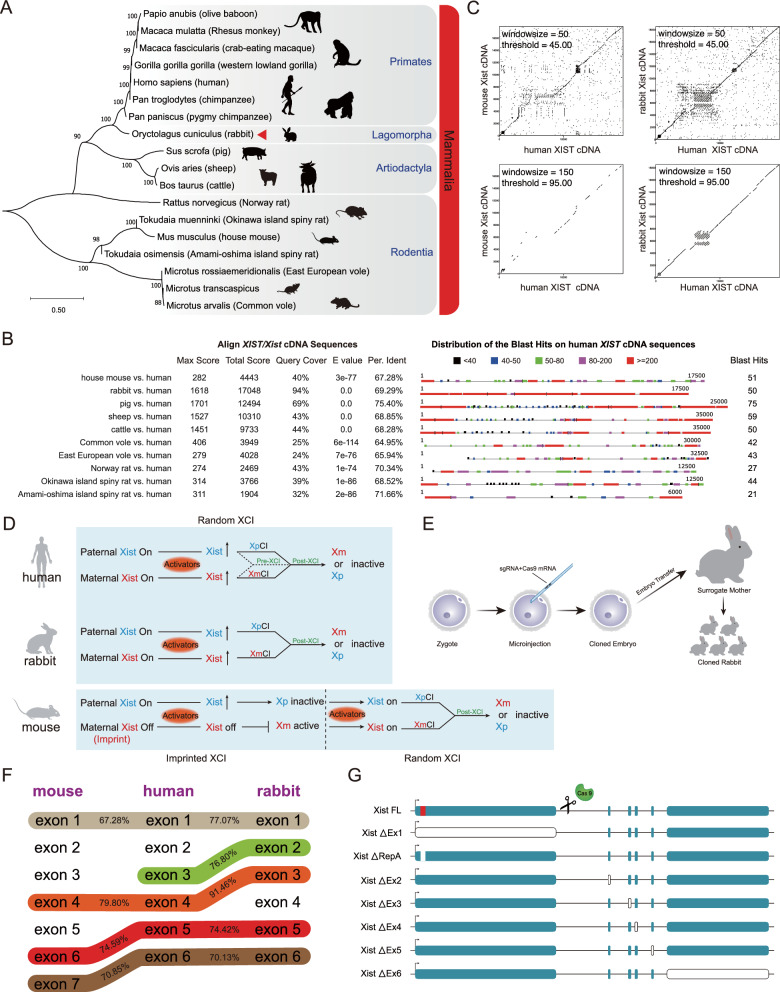
Comparison of *Xist* among different species. **A** Evolutionary relationships of the *XIST/Xist* gene among Primates, Lagomorpha, Artiodactyla, and Rodentia. Mammalian phylogeny was estimated using maximum likelihood from 18 nucleotide sequences. Clades discussed in the text are labeled. Bootstrap support values ≥ 88% are indicated at nodes. The scale bar indicates evolutionary distance. **B** *Xist* sequences were aligned to humans using the NCBI BLAST server. **C** Dot plot analysis of *Xist/XIST* cDNA sequences in mouse, rabbit, and human using the EMBOSS dot-matcher program. **D** Hypothesis explaining differences in *Xist/XIST* regulation and XCI initiation observed in mouse, rabbit, and human embryos, based on previous work [48]. **E** Schematic representation of the sequence homology of *Xist/XIST* exons across mice, humans, and rabbits. The percent identity mentioned in the text is indicated. **F** Schematic diagram illustrating the establishment of a cloned rabbit model using standard microinjection procedures. **G** Schematic representation of the *Xist* knock-out created in rabbits using CRISPR/Cas9; the different exons are highlighted in colored boxes, with repeat A highlighted in the red box. Deleted exons are represented as white boxes

According to a study published in Nature by Okamoto et al., rabbits display a similar XCI mechanism to humans during early embryogenesis [48]. In both species, X chromosome inactivation occurs randomly during both stages of *XIST* expression. However, in contrast, the house mouse exhibits paternal Xist expression during the initial stage of X chromosome inactivation, followed by random X chromosome inactivation during the subsequent stage (Fig. 1D). These findings emphasize the variations in the XCI mechanism that regulate *Xist* expression in mice and humans. Therefore, the rabbit serves as an ideal animal model for studying XCI.

In this study, we performed a thorough phylogenetic analysis that unveiled a strong connection between rabbits and primates. Moreover, our analysis demonstrated that human *XIST* shares a greater sequence similarity with rabbits than with mice. This suggests that rabbits would serve as an excellent animal model for investigating the XCI mechanism. Additionally, we employed CRISPR/Cas9 to delete exon 1–6 and repeat A of the rabbit *Xist* RNA transcript, enabling us to determine its functionality. In conclusion, these findings enhance our comprehension of the functional mechanisms involved in *Xist*-induced XCI at the animal level.

## Materials and methods

### Animals care and use

The Institutional Animal Care and Use Committee of Jilin University approved all animal experiments. New Zealand White rabbits were obtained from the Laboratory Animal Centre of Jilin University (Changchun, China). All animal experiments were conducted by the guidelines for animal experiments of the Laboratory Animal Center of Jilin University.

### Plasmid design and construction

Eleven pairs of sgRNAs were designed to knock out *Xist* different regions according to the previous description [49], which were cloned into the BbsI-linearized pUC57-T7-gRNA vector. Then, sgRNAs were amplified using PCR with T7 primers (T7-Fwd: 5′-GAA ATT AAT ACG ACT CAC TAT A-3’ and T7-Rev: 5′-AAA AAA AGC ACC GAC TCG GTG CCA C-3’) and in vitro transcribe using the MAXIscript T7 kit (Invitrogen) and purified with a miRNeasy mini kit (QIAGEN) according to the manufacturer’s instructions. To produce SpCas9 mRNA, the PCS2 + Cas9 (Plasmid #122,948) plasmid was linearized with NotI restriction digestion and used as a template to in vitro transcribe mRNAs using mMESSAGE mMACHINE SP6 Transcription Kit (Invitrogen) and then Cas9 mRNAs were purified with a miRNeasy mini kit (QIAGEN) according to the manufacturer’s instructions. All sgRNA sequences are listed in Supplementary Table 1.

### Microinjection of rabbit zygotes

The protocol for microinjecting sgRNA/Cas9 mRNA into pronuclear stage embryos is detailed in our previously published study [50]. Briefly, a mixture of Cas9 mRNA (200 ng/ul) and sgRNA (50 ng/ul) was co-injected into the cytoplasm of pronuclear stage zygotes. Finally, 40–50 injected zygotes were transferred into the oviduct of recipient rabbits.

### Single-embryo and rabbit genotyping by PCR

The zygotes injected with sgRNA/Cas9 mRNA were cultured for 4 days and subsequently collected for genotyping analysis. Embryos were incubated in lysis buffer at 50 °C for 20 min and then at 90 °C for 5 min in a PCR machine. Genomic DNA was extracted from newborn rabbits for PCR genotyping, followed by Sanger sequencing and T-A cloning. Please refer to Supplementary Table 1 for a list of all primers.

### RT-PCR and quantitative real-time PCR analysis

Tissue RNA was extracted with TRIzol (Invitrogen) according to the manufacturer’s instructions, and cDNA synthesis was performed on extracted RNA using FastKing cDNA First Strand Synthesis Kit (TIANGEN, KR116). Embryo RNA was extracted using the MicroElute® Total RNA Kit (Omega, R6831) according to the manufacturer’s instructions, and cDNA synthesis was performed on extracted RNA using PrimeScript™ RT Master Mix (Takara, RR036A). A QuantStudio 3 Real-Time PCR System (Thermo Fisher Scientific) was utilized for conducting quantitative real-time PCR experiments. For each gene, three biological replicates and three technical replicates (3 × 3) were carried out. The GAPDH gene was employed as an internal control to standardize the expression data. The gene-specific primers for RT-PCR and qRT-PCR can be found in Supplementary Table 2 and 3, respectively.

### Hematoxylin and eosin (H&E) staining

The hematoxylin and eosin (H&E) staining was performed according to our published protocols [51]. Briefly, the tissues from WT and mutant rabbits were fixed in 4% paraformaldehyde for 48 h, embedded in paraffin wax, and then sectioned for slides. The slides were stained with hematoxylin and eosin, and were viewed under a Nikon TS100 microscope.

### Statistical analysis of weight and survival

To analyze survival, we conducted regular daily monitoring of the rabbits. The survival data are from 6 KO rabbits and 6 control rabbits. Body weight was recorded weekly. All data are expressed as mean ± SEM from at least three determinations in all experiments. The data were analyzed by Student’s unpaired t-test using GraphPad Prism software. p < 0.05 indicated statistical significance (∗ p < 0.05, ∗  ∗ p < 0.01, ∗  ∗  ∗ p < 0.001).

### Phylogenetic tree construction

To conduct the phylogenetic analysis of lncRNA *Xist*, we downloaded all *Xist* sequences of various species from the NCBI database. Using MEGA, we constructed maximum likelihood phylogenetic trees, with 1000 bootstrap replicates [52–54]. The tree is drawn to scale, and the branch lengths (next to the branches) are in the same units as the evolutionary distances used to infer the phylogenetic tree [52].

### Dot plots

To access sequence similarity, dot plots were generated using EMBOSS dot-matcher [55]. To enhance visualization clarity, two different thresholds were utilized to generate distinct dot plots. In this analysis, all positions of the first input sequence were systematically compared with all positions of the second input sequence using a specified substitution matrix. The resulting dot plots were generated as a rectangular grid, with the two sequences serving as the axes. Each dot in the plot represents a position where a similarity was identified between the corresponding positions of the two sequences.

### Motif analysis

Conserved motifs of each *Xist* repeat domain were defined using the MEME program [56]. Run with the following non-default parameters: meme sequences.fa -dna -oc. -nostatus -time 14,400 -mod anr -nmotifs 100 -minw 4 -maxw 15 -objfun classic -minsites 2 maxsites 600 -revcomp -markov_order 0.

### RNA secondary structure

The secondary structure graph is created using the ViennaRNA Package from RNA secondary structure predictions [57]. The red circles (bases) indicate a confidence level of 90% or higher, based on minimum free energy (MFE) and partition function.

## Results

### Rabbits are the ideal non-primate animal model for studying *Xist* in vivo

To investigate the functional role of *Xist*, we performed a phylogenetic analysis using Molecular Evolutionary Genetics Analysis software (MEGA11). We employed the maximum composite likelihood method with 1000 bootstrap replicates [58]. Reference sequences of the *Xist* gene were obtained from the NCBI database [59]. The phylogenetic tree analysis of the *XIST/Xist* gene showed a close relationship between rabbit species and humans, while excluding non-human primates (Fig. 1A). Additionally, the three species, namely pigs, cows, and sheep, were found to belong to the same sister branch in the taxonomic status of the evolutionary tree, indicating a close evolutionary relationship. To validate our evolutionary classification, we used various methods to analyze the phylogenetic relationships based on different theoretical models between species (Fig. S1A and B). These results were consistent with the findings observed in Fig. 1A. The results revealed that rabbits and primates are part of the same main branch of the phylogenetic tree, indicating a closer relationship of the *XIST/Xist* gene between rabbits and primates.

Additionally, we compared the *Xist* DNA sequences of different non-primate species. Our findings show that rabbits have the highest overall score and sequence coverage, with a total score of 17,048 and a coverage of 94%. In contrast, the house mouse, which is a commonly used animal model, scored only 4443 with a coverage of 40%, indicating a lower DNA homology to human *Xist.* This suggests that rabbit *Xist* has a higher similarity to humans (Fig. 1B). Furthermore, we also observed a higher homology of pig *Xist* to humans. To further understand the sequence conservation among humans, rabbits, and pigs, we conducted multiple sequence alignments (Fig. S1C). In the alignment results, we noticed a significant region of low similarity in the *Xist* sequence of pigs, spanning from 4700 to 15,600. Although some local regions show higher similarity to humans, the overall genomic structural homogeneity is lost in pig *Xist*. Additionally, the genetic distance matrix confirms that the *Xist* sequence of rabbits is more closely related to humans (Fig. S1D). This finding aligns with the evolutionary relationship between species [60–62].

To further evaluate the similarity of Xist sequences among the house mouse, rabbit, and human, we conducted a dot-plot analysis [55]. We consistently obtained results across various window sizes and thresholds, confirming that rabbits exhibit a higher sequence similarity to humans than mice (Fig. 1C).

In addition, rabbits exhibit a similar XCI mechanism to humans during early embryogenesis [48]. Both humans and rabbits undergo random X chromosome inactivation during both stages of *XIST* expression. On the other hand, mice display paternal *Xist* expression during the initial stage of X chromosome inactivation, followed by random X chromosome inactivation during the subsequent stage (Fig. 1D).

Then, we conducted a comparative analysis to assess the homology of exons among mice, rabbits, and humans *XIST/Xist* (Fig. 1F and Fig. S3A). The results showed that human *XIST* exon 1 had a higher homology of 77.07% with rabbit *Xist* exon 1 compared to the 67.28% homology observed with mouse *Xist* exon 1. Interestingly, we did not find any homologous sequence of human *XIST* exon 2 in either mice or rabbits. On the other hand, human *XIST* exon 3 showed a homology of 76.80% with rabbit *Xist* exon 2, but no homologous sequence was detected in mice. Furthermore, human *XIST* exon 4 had a higher homology of 91.46% with rabbit *Xist* exon 3 compared to the 79.80% homology with mouse *Xist* exon 4. Additionally, human *XIST* exon 5 demonstrated homologies of 74.59% and 74.42% with mouse *Xist* exon 6 and rabbit Xist exon 5, respectively. Similarly, human *XIST* exon 6 showed homologies of 70.85% and 70.13% with mouse *Xist* exon 7 and rabbit *Xist* exon 6, respectively.

Besides, we conducted a comparison to determine the similarity of other repeat sequences on the *Xist* loci in human, mouse, and rabbit (Fig. S2A). Initially, we assessed the homology between different repeat sequences and found that the homology between rabbit repeat F and human repeat F was 85.71%. However, no corresponding homologous sequence of human repeat F was found in mice. Similarly, the homology between rabbit repeat C and human repeat C was 76.47%, but no corresponding homologous sequence was identified in mice. The homology between rabbit repeat D and human repeat D was 72.77%, while mouse repeat D exhibited 79.12% homology to its human counterpart. Rabbit repeat E showed a 71.23% homology to the human sequence, whereas no corresponding homologous sequence was found in mice (Fig. S2B). Furthermore, we compared the motif similarity of the different repeat sequences and observed that the rabbit repeats were more similar in length to human repeats (Fig. S2C). Notably, repeat B consisted of repeat units "enriched in cytosine bases," and repeat E exhibited greater similarity between rabbit and human sequences. In contrast, the mouse sequence exclusively consisted of repeat units "enriched in thymine bases." These findings position rabbits as an ideal non-primate animal model for studying *Xist* in vivo.

In summary, these findings highlight the significance of rabbits as an invaluable model for understanding the functions of *Xist*. To generate cloned animals, we co-injected Cas9 mRNA and sgRNA into one-cell stage embryos and transferred them into surrogate mother rabbits (Fig. 1E and Fig. S3B). We utilized CRISPR/Cas9 technology to disrupt the exon sequences of *Xist* in rabbits [63, 64]. Specifically, we targeted exons 1–6 and repeat A to investigate the functional role of *Xist* (Fig. 1G).

### Deletion of exon 1 in female rabbits does not survive

**Fig. 2:**
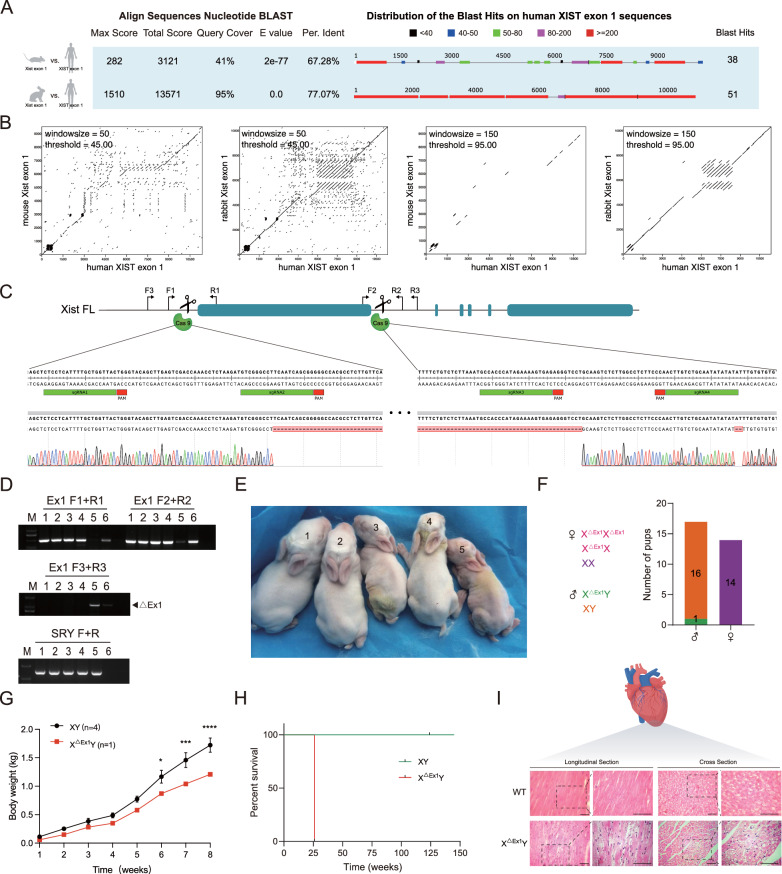
Male rabbit with *Xist* exon 1 deletion exhibit a loose arrangement of the myocardial fibers. **A** The BLAST results of *Xist/XIST* exon 1 are compared between mouse, human, and rabbit. **B** Dot plot analysis of the *Xist/XIST* exon 1 sequence in mouse, rabbit, and human. **C** Target loci and Sanger sequencing results demonstrate the knock-out of *Xist* exon 1 in F0 male rabbits. All sgRNA sequences are listed in Supplementary Table 1. **D** Founder rabbits from the F0 generation are identified through agarose gel electrophoresis. **E** The gross appearance of rabbits from the F0 generation at day 7 reveals that *Xist* exon 1 knock-out rabbits exhibit developmental delay. **F, left** Schematic for generating heterozygous *Xist* exon 1 deletants. (**Right**) Genotype data for F0; the number of pups for each genotype is listed. **G** The body weight of male X^△Ex1^Y F0 rabbit and male littermate controls (*n* = 4). Error bars indicate mean ± SEM. **H** The survival curve for X^△Ex1^Y and male littermate controls. **I** H&E staining for cardiac muscle from X^△Ex1^Y and male control animal. Scale bars, 50um

Sequence homology analysis was conducted on *Xist* exon 1 in mice, humans, and rabbits using the NCBI BLAST service. The coverage rate of rabbit *Xist* exon 1 was determined to be 95% when compared to the human *XIST* sequence, which was significantly higher than the 41% observed in mice. Additionally, the percentage identity between rabbit *Xist* exon 1 and human *XIST* exon 1 was found to be 77.07%, while the percentage identity between mice and humans was 67.28% (Fig. 2A). Dot-plot analysis confirmed that rabbit *Xist* exon 1 showed greater homology to humans compared to mice (Fig. 2B).

To produce knock-out rabbits using CRISPR/Cas9 technology, we designed four single-guide RNAs (sgRNAs) that targeted exon 1 of *Xist.* Genotyping was conducted by PCR using four sets of primers specific to certain genes, including the *Sry* gene, which is only present in males. Sanger sequencing was then performed to confirm the genotyping results (Fig. 2C and D). Among the genotyping results of the founder animals (Fig. 2F), we did not identify any homozygous knockout female rabbits. However, we were successful in obtaining a hemizygous knockout male rabbit, named #5 (Fig. 2E). Unfortunately, this male rabbit showed developmental delays (Fig. 2G) and eventually died at 25 weeks (Fig. 2H). Examination of the #5 rabbit's myocardium revealed a loose arrangement of cardiac fibers (Fig. 2I).

Given the consistent challenges encountered in obtaining homozygous knockout female individuals, our hypothesis was that the X^∆Ex1^X^∆Ex1^ homozygous mutant females could be generated but would die early in embryogenesis. To investigate this possibility, we conducted an embryonic-level investigation by employing a fibroblast injection method to introduce sgRNA and Cas9 RNA into zygotes. After a week-long incubation period, genotyping was performed using PCR and Sanger sequencing. Remarkably, the results revealed successful large-scale deletions at the embryonic level (Fig. S3C and D). The results showed that the X^∆Ex1^X^∆Ex1^ homozygous mutant females can be generated but perish early in embryogenesis.

### Deletion of *Xist* repeat A in rabbits results in embryonic lethality.

**Fig. 3:**
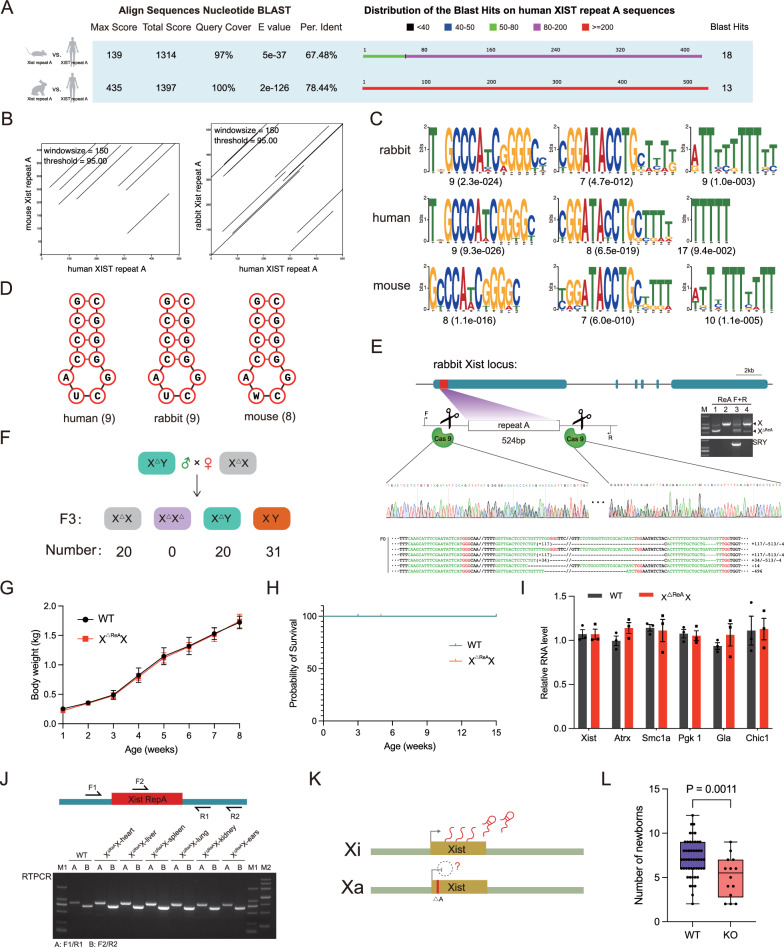
*Xist* repeat A homozygous knockout rabbit does not survive. **A** The BLAST result compares the *Xist*/*XIST* repeat A sequences in mouse, human, and rabbit. **B** Dot plot analysis illustrates the sequence similarities between mouse, rabbit, and human *Xist*/*XIST* repeat A. **C** The top motifs identified by MEME in *Xist*/*XIST* repeat A are compared, with the number of sites and E-value displayed below each motif logo. **D** Secondary structure analysis of *Xist*/*XIST* repeat A is presented for human, rabbit, and mouse. The total numbers of stem loops in repeat A are listed. **E** Target loci and Sanger sequencing results provide evidence of *Xist* repeat A knock-out in F0 rabbit. All sgRNA sequences are listed in Supplementary Table 1. **F** A schematic depicts the process of generating homozygous *Xist* repeat A deletants. Genotype data for F3; the number of pups for each genotype is listed. **G** Body weight of X^△ReA^X and WT rabbits. Error bars indicate mean ± SEM. **H** Survival curve for X^△ReA^X and WT rabbits. **I** Expression of X-linked genes in X^△ReA^X and WT rabbits. Error bars indicate mean ± SEM. **J** Gene expression analysis by RT-PCR. **K** A model for *Xist*-mediated transcriptional silencing across the X chromosome in X^△ReA^X rabbits. **L** Newborn data from cross-breeding between X^△ReA^X / X^△ReA^Y compared to WT control (box and whiskers plot, min. to max., all points shown)

To investigate the impact of *Xist* repeat A on individual development, we conducted a comparative analysis of sequence homology in mice, humans, and rabbits. Interestingly, our findings showed that the rabbit and human repeat A sequences had a higher level of homology (78.44%), while the homology between mouse and human sequences was 67.48% (Fig. 3A). This was further supported by the results of the dot-plot analysis, which confirmed that the rabbit repeat A sequence bore a closer resemblance to the human sequence (Fig. S4A and Fig. 3B). Moreover, a more similar pattern of repeat motifs was observed in rabbits and humans (Fig. 3C and Fig. S4B). Additionally, the major stem-loop structures in both rabbit and human repeat A sequences were consistent, whereas the mouse sequence showed discrepancies (Fig. 3D). Specifically, the RNA hairpin in rabbit and human sequences consisted of a 12-nucleotide AUCG tetraloop, while the mouse sequence had an AWCG tetraloop. These results clearly demonstrate that the rabbit *Xist* repeat A sequence is more similar to the human sequence. Subsequently, two pairs of single guide RNAs (sgRNAs) were designed to target and delete the *Xist* repeat A. The genotyping was determined by performing PCR, and the results were further confirmed through Sanger sequencing (Fig. 3E).

To determine the viability of homozygote knockout *Xist* repeat A (X^∆ReA^X^∆ReA^) females, we attempted to generate offspring through hybridization (Fig. 3F and Fig. S4C-G). Despite multiple attempts, we were unsuccessful in producing X^∆ReA^X^∆ReA^ female rabbits (Fig. 3F and Fig. S4D).

To investigate the timing of development cessation in X^∆ReA^X^∆ReA^ females, we partially tracked early embryonic development. Initially, we examined early embryos at rabbit embryonic day 12 (E12) for characterization. The results indicated the absence of homozygote individuals. Interestingly, all heterozygous individuals exhibited skewed X chromosome inactivation, as evidenced by the transcription of *Xist* RNAs from intact X chromosomes (Fig. S5A-C). Subsequently, we analyzed embryos from the E9.5 period and obtained similar outcomes; no homozygote individuals were present, and all heterozygous individuals showed skewed X chromosome inactivation (Fig. S5D and E).

Furthermore, we observed no significant differences in body weight (Fig. 3G), survival rates (Fig. 3H), and X-linked gene expression levels (Fig. 3I) between X^∆ReA^X females and WT rabbits. The normal development of the heart, liver, spleen, lungs, and kidneys in X^∆ReA^X females was comparable to that of WT rabbits (Fig. S4H).

RT-PCR results confirmed the previous observation of skewed X chromosome inactivation in all samples (Fig. 3J). These findings suggest that Xist repeat A is transcribed from the complete X chromosome (Fig. 3K). Additionally, the average number of offspring in the X^∆ReA^X female and X^∆ReA^Y male cross-group (5.071 offspring) was lower than that in the WT group (7.118 offspring), further supporting the conclusion that embryos lacking *Xist* repeat A function do not survive (Fig. 3L).

### Deletions of *Xist* exon 2 in rabbits are viable and develop normally

**Fig. 4:**
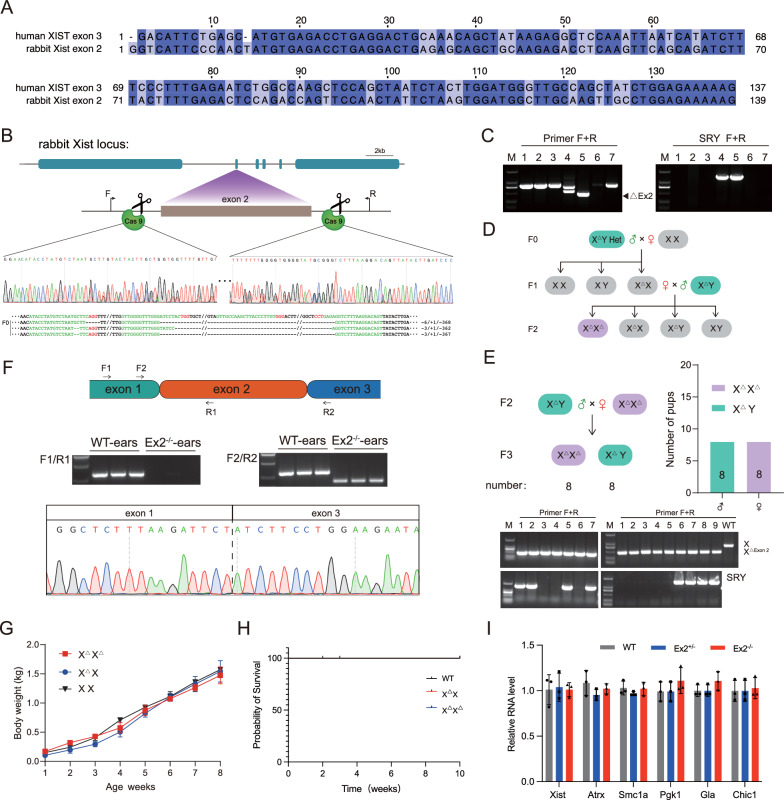
Viability of *Xist* exon 2 knockout rabbits. **A** Sequence alignment comparing human *XIST* exon 3 and rabbit *Xist* exon 2, with identical bases highlighted on a dark background. **B** Sanger sequencing results and target loci confirm *Xist* exon 2 knock-outs in F0 rabbits. All sgRNA sequences are listed in Supplementary Table 1. **C** Agarose gel electrophoresis result of PCR product in the F0 generation. (**D**) Schematic illustrating the generation of the F1 and F2 generations. **E, up** Schematic diagram outlining the breeding strategy employed to generate the F3 generation. Genotype data for F3, along with the number of pups for each genotype, is provided. (**Down**) Agarose gel electrophoresis result of PCR product in the F3 generation. **F, up** The *Xist* expression was analyzed using RT-PCR. The agarose gel electrophoresis of the PCR products is presented. (**Down**) The Sanger sequencing results confirmed the findings from the RT-PCR analysis. **G** Comparison of body weight between KO and WT rabbits, with error bars representing the mean ± SEM. **H** Survival curve for KO and WT rabbits. (**I** The qPCR results of X-linked genes in Ex2^±^ and Ex2^−/−^ rabbits are displayed. The error bars represent the mean ± SEM

Analysis of sequence homology revealed that there is no homologous sequence of human *XIST* exon 3 in mice. However, there is a higher homology (76.80%) between rabbit *Xist* exon 2 and human *XIST* exon 3 (Fig. 4A), which was also confirmed by dot plot analysis (Fig. S6A). Therefore, deletions of *Xist* exon 2 in rabbits were used as a model to mimic the function of XIST exon 3 in humans.

Seven founder (F0) pups were identified using Sanger sequencing. We obtained a chimeric male with exon 2 deletion (Fig. 4B and C). By further backcrossing, we successfully produced female homozygous knockout rabbits (Ex2^−/−^) in the F2 generation (Fig. 4D and Fig. S6B). RT-PCR results confirmed the absence of exon 2 sequence in the expressed *Xist* RNA of the rabbits (Fig. 4F). It is noteworthy that there were no significant differences in terms of body weight (Fig. 4G), survival rates (Fig. 4H), X-linked gene expression (Fig. 4I), and reproductive efficiency (Fig. 4E) between the Ex2^−/−^ and WT rabbits. These findings indicate that the Ex2^−/−^ rabbits exhibit normal growth and development.

### Deletions of *Xist* exon 3 in rabbits are viable and develop normally

**Fig. 5:**
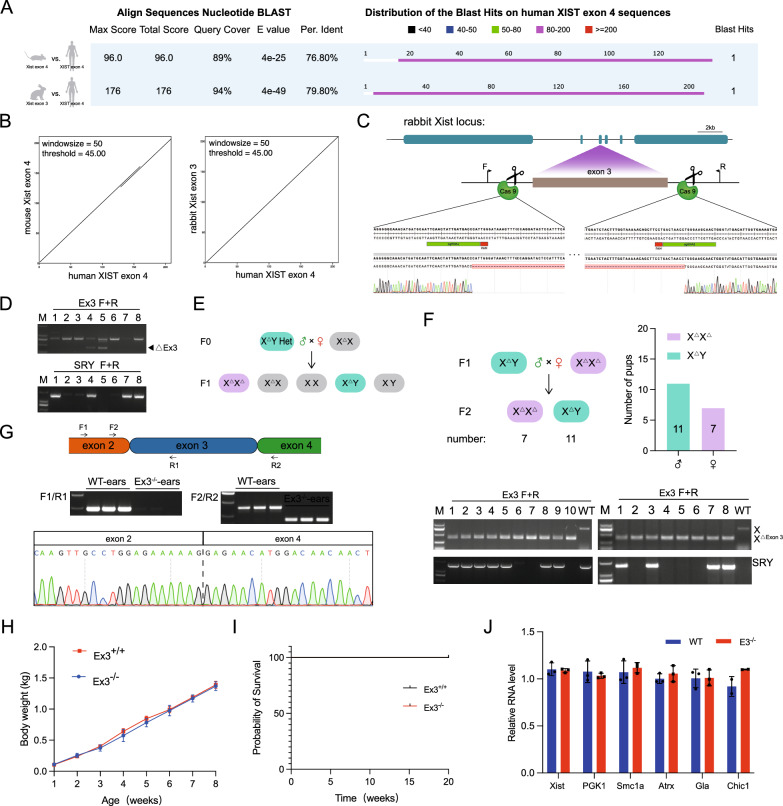
Viability of *Xist* exon 3 knockout rabbits. **A** BLAST results comparing human *XIST* exon 4, rabbit *Xist* exon 3, and mouse *Xist* exon 4. **B** Dot plot analysis of mouse, rabbit, and human sequences. **C** Sanger sequencing results and target loci confirm *Xist* exon 3 knock-outs in F0 rabbits. All sgRNA sequences are listed in Supplementary Table 1. **D** Agarose gel electrophoresis shows PCR product of F0 generation. **E** Schematic representation of generating the F1 generation. **F, Up** Schematic diagram illustrating the breeding strategy employed to generate the F2 generation. Genotype data for the F2 generation is provided, listing the number of pups for each genotype. (**Down**) Result of agarose gel electrophoresis showing the PCR product for the F2 generation. **G**, **up** RT-PCR analysis of *Xist* expression, with agarose gel electrophoresis of the PCR products shown. (**Down**) The Sanger sequencing results support the RT-PCR analysis findings. **H** Comparison of body weight between KO and WT rabbits. Error bars represent mean ± SEM. **I** Survival curve comparing KO and WT rabbits. **J** qPCR result displaying the expression of X-linked genes in Ex3^−/−^ rabbits. Error bars represent the mean ± SEM

The results of the sequence homology analysis revealed that there was a 79.80% sequence homology between rabbit *Xist* exon 3 and human *XIST* exon 4, with a coverage of 94%. This was significantly higher compared to the 76.80% sequence homology and 89% coverage observed between human *XIST* exon 4 and mouse *Xist* exon 4 (Fig. 5A and Fig. S6C). These findings were further confirmed by the dot plot analysis (Fig. 5B).

By using Sanger sequencing, we were able to identify the founder (F0) pups, and obtain a male chimeric rabbit with exon 3 knockout and a single knockout female rabbit (Fig. 5C and D). Subsequent breeding allowed us to successfully obtain female homozygous knockout rabbits (Ex3^−/−^) in the F1 generation (Fig. 5E and Fig. S5D). The RT-PCR results demonstrated the absence of exon 3 sequence in the expressed *Xist* RNA of the rabbits (Fig. 5G). Importantly, there were no significant differences observed in terms of body weight (Fig. 5H), survival rates (Fig. 5I), X-linked gene expression (Fig. 5J) and reproductive efficiency (Fig. 5F) between the Ex3^−/−^ and WT rabbits. These findings indicate normal growth and development of the Ex3^−/−^ rabbits.

### Deletions of *Xist* exon 4 in rabbits are viable and develop normally

Rabbit *Xist* exon 4 is the only exon that does not have sequence homology with its human counterpart. To investigate the functionality of rabbit *Xist* exon 4, we employed a targeted approach using a pair of sgRNAs to specifically disrupt this exon. The F0 generation rabbits were identified using Sanger sequencing, and we obtained a male chimeric individual with a complete knockout of exon 4 and a single knockout female individual (Fig. S7A and B). By subsequent breeding, we successfully obtained female homozygous knockout rabbits (Ex4^−/−^) in the F1 generation (Fig. S7C and Fig. S6E). RT-PCR results demonstrated the absence of exon 4 sequence in the expressed *Xist* RNA of the rabbits (Fig. S7E). Importantly, we found no significant differences in terms of body weight (Fig. S7F), survival rates (Fig. S7G), X-linked gene expression (Fig. S7H), and reproductive efficiency (Fig. S7D) between the Ex4^−/−^ and WT rabbits. These findings indicate normal growth and development of the Ex4^−/−^ rabbits.

### Deletions of *Xist* exon 5 in rabbits are viable and develop normally

**Fig. 6:**
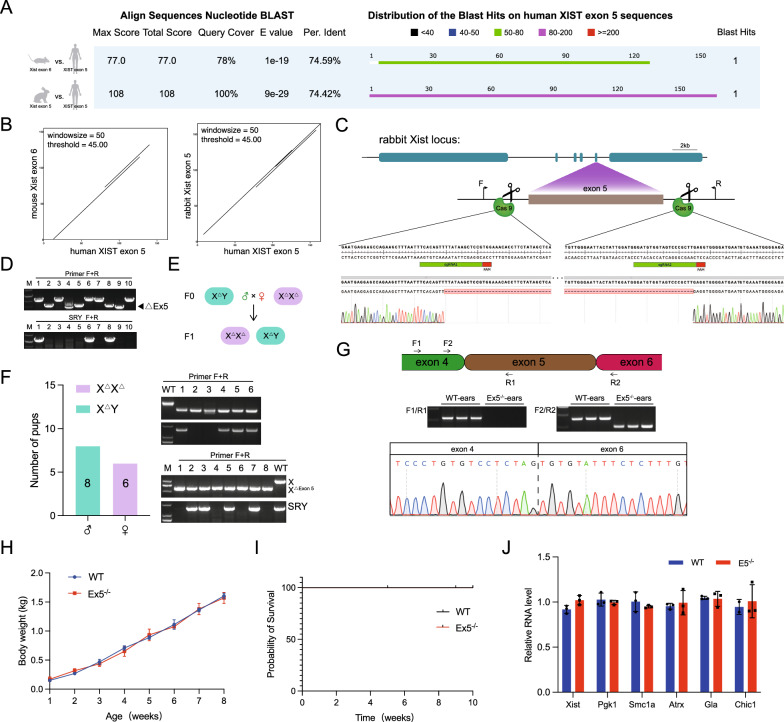
Viability of *Xist* exon 5 knockout rabbits. **A** BLAST analysis comparing human *XIST* exon 5, rabbit *Xist* exon 5, and mouse *Xist* exon 6. **B** Dot plot analysis of mouse, rabbit, and human sequences. **C** Sanger sequencing results and target loci confirm *Xist* exon 5 knock-outs in F0 rabbits. All sgRNA sequences are listed in Supplementary Table 1. **D** Agarose gel electrophoresis result of PCR product in the F0 generation. **E** Schematic representation of the process for generating the F1 generation. **F**, **left** Genotype data for F2 rabbits; the number of pups for each genotype is provided. (**Right**) Agarose gel electrophoresis result of PCR product in the F2 generation. **G**, **up** RT-PCR analysis of the *Xist* expression. Agarose gel electrophoresis of the PCR products is shown. (**Down)** The Sanger sequencing results supported the RTPCR analysis results. **H** The body weight of KO and WT rabbits. Error bars represent mean ± SEM. **I** The survival curve for KO and WT rabbits. **J** qPCR result of X-linked genes in Ex5^−/−^ rabbits. Error bars represent mean ± SEM

Sequence homology analysis revealed that rabbit *Xist* exon 5 had a 74.42% sequence homology with human *XIST* exon 5, with 100% coverage. This was significantly higher than the 74.59% and 78% sequence homology observed between human *XIST* exon 5 and mouse *Xist* exon 6 (Fig. 6A and Fig. S6F). Dot plot analysis confirmed these findings (Fig. 6B).

Sanger sequencing identified the founder (F0) pups, and homozygous exon 5 knockout male and female rabbits were obtained (Fig. 6C and D). Breeding subsequently led to the generation of F1 rabbits (Fig. 6E). RT-PCR results demonstrated the absence of exon 5 sequence in the expressed *Xist* RNA of the rabbits (Fig. 6G). Importantly, no significant differences were observed in terms of body weight (Fig. 6H), survival rates (Fig. 6I), X-linked gene expression (Fig. 6J), and reproductive efficiency (Fig. 6F) between the Ex5^−/−^ and WT rabbits. These findings indicate normal growth and development of the Ex5^−/−^ rabbits.

### Deletion of *Xist* exon 6 in rabbits results in embryonic lethality

**Fig. 7:**
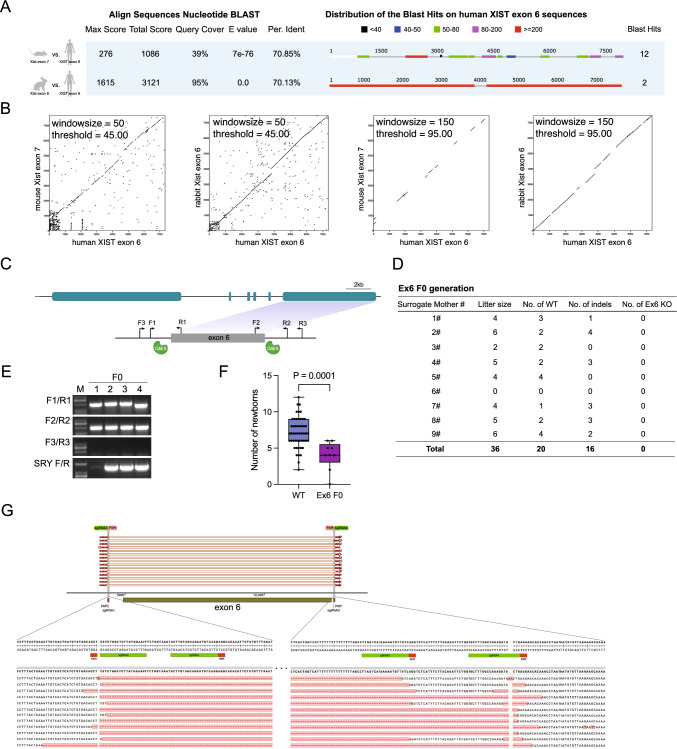
*Xist* exon 6 knockout rabbits do not survive. **A** The BLAST result shows the comparison of human *XIST* exon 6, rabbit *Xist* exon 6, and mouse *Xist* exon 7. **B** Dot plot analysis illustrates the sequences of mouse, rabbit, and human. **C** The map indicates the target sites of CRISPR/Cas9 in the *Xist* loci. All sgRNA sequences are listed in Supplementary Table 1. **D** A table summarizes the litter size of live-born offspring in different genotypes of the F0 generation. **E** Representative images of agarose gel electrophoresis display the PCR products from the F0 generation. **F** The newborn data from the F0 generation is compared to the WT control using a box and whiskers plot, showing the minimum to maximum values and t-test results (*P* = 0.0001). **G** The target loci and Sanger sequencing results confirm the knock-out of *Xist* exon 6 in rabbit embryos

The results of the sequence homology analysis showed that there was a 70.13% sequence homology between rabbit *Xist* exon 6 and human *XIST* exon 6, with a coverage of 95%. This was significantly higher compared to the 70.85% sequence homology and 39% coverage observed between human exon 6 and mouse *Xist* exon 7 (Fig. 7A). These findings were also confirmed by dot plot analysis (Fig. 7B). Thus, the deletions of *Xist* exon 6 in rabbits were used to mimic the function of *XIST* exon 6 in humans.

To disrupt the function of *Xist* exon 6 in rabbits, we designed two pairs of sgRNAs targeting *Xist* exon 6 (Fig. 7C). The F0 generation rabbits were subsequently confirmed through PCR and Sanger sequencing. Surprisingly, no *Xist* exon 6 knockout animals were obtained from pregnant females (Fig. 7D and E), indicating that the absence of exon 6 resulted in non-viability. Additionally, statistical analysis of offspring production revealed a reduced number of offspring from pregnant females compared to wild-type (WT) rabbits (Fig. 7F).

To further characterize the lethal stage of early embryonic development, we injected Cas9 mRNA and sgRNA into fertilized zygotes. Surprisingly, we were able to successfully knockout rabbit *Xist* exon 6 in early-stage embryos (Fig. 7G and Fig. S6G). However, our results revealed that the cloned embryos had a significantly lower blastocyst rate (13.3 ± 0.9%) compared to the control group (74.7 ± 5.4%). These findings provide conclusive evidence that the deletion of *Xist* exon 6 leads to embryonic lethality in rabbits, hindering proper development.

## Discussion

*Xist* is continuously expressed in female somatic cells to maintain X chromosome inactivation (XCI) [65]. Previous studies on *Xist*'s functional domains have primarily used mouse models and cellular-level experiments. However, it is important to note that mice display paternal *Xist* expression during the initial stage of XCI, followed by random X chromosome inactivation during the subsequent stage, which differs significantly from the random XCI mechanism observed in humans [66, 67]. Therefore, the consistency of cellular-level results in vivo remains uncertain. Initially, we chose to focus on the homozygous deletion of different exons due to the high degree of homology among different species. However, the exact functional mechanism was unclear, especially regarding rabbit *Xist* exon 2, which corresponds to human *XIST* exon 3. In mice, there is no homologous sequence for human *XIST* exon 3. Therefore, we embarked on a systematic characterization of these exons. Subsequently, numerous studies suggest that repeat A is crucial for gene silencing. Deletion of repeat A has been found to result in X chromosome silencing failure [24, 25, 42, 68, 69]. However, the implications of this finding in vivo are unknown. In our study, we deleted repeat A in rabbits and found that individuals homozygous for the deletion exhibited embryonic lethality and were unable to develop. Interestingly, individuals heterozygous for the repeat A deletion transcribed the *Xist* gene from the intact X chromosome, indicating skewed X chromosome inactivation. This suggests that the choice of which X chromosome to inactivate is consistent across all early embryonic stem cells, either Xp or Xm, rather than a combination of the two. There were no phenotypic differences compared to individuals with the wild-type genotype. When we analyzed the reproductive quantities in the offspring, we found a lower number compared to the control group, suggesting that X^∆ReA^X^∆ReA^ or X^∆ReA^X (*Xist* is transcribed by X^∆ReA^) may lead to embryonic lethality. These findings indicate that repeat A plays a crucial role in establishing X chromosome inactivation and female development.

Deletion of the 5' conserved region of *Xist*, which includes exon 1, in mice revealed that female mice lacking *Xist* RNA were able to develop and survive until birth [70]. However, there was a lower frequency of female births and they had a smaller size at birth, although most organ development was normal. In our study, when exon 1 was deleted in rabbits, it led to delayed development and premature death in males. Histological analysis further revealed impaired heart development. Deletion of *Xist* exon 1 had an impact on male development and led to decreased birth rates. Moreover, females were found to be incapable of surviving, indicating the crucial role of exon 1 in the process of X-chromosome inactivation in females. Specifically, the deletion of exon 1 hindered both the development and survival of females.

The functionality of regions other than *Xist* repeat A is currently poorly understood. According to a few reports, repeat B and C play a critical role in recruiting epigenetic modifier proteins to maintain the epigenetic state of XCI [35], while repeat E is essential for *Xist* localization and gene silencing [41]. However, the functions of other regions are still unknown. In a previous study, it was suggested that *XIST* exon 5 is crucial for maintaining XCI status in human K562 cells, while exons 2, 3, and 4 seem to be dispensable [44]. However, the implications of these findings in vivo remain unknown. The results of the study indicate that rabbits lacking these exons can be born and survive normally, showing no significant differences in body weight, survival rate, or X-linked gene expression compared to WT individuals. This suggests that these exons may not be necessary for normal functioning in living organisms. Moreover, the previously emphasized importance of exon 5 in maintaining XCI status at the cellular level seems to have little significance in vivo. Additionally, the functional role of *Xist* exon 6 remains unknown. Our study found that the deletion of exon 6 in living organisms led to a lower rate of embryo blastocysts and the absence of offspring lacking exon 6. These findings demonstrate the vital and essential role of *Xist* exon 6 in embryonic development and individual survival. In conclusion, our study comprehensively elucidated the functional roles of *Xist* exons and repeat A in vivo, enhancing our understanding of the functional landscape of different regions within *Xist* and offering new insights into the functional mechanisms of *Xist* in X chromosome inactivation.

### Supplementary Information

Below is the link to the electronic supplementary material.

Supplementary file1 (PDF 49677 KB)

Supplementary file2 (PDF 131 KB)

## Data Availability

The authors state that all data necessary for confirming the conclusions presented in this article are represented fully within the article or can be provided by the authors upon request.

## References

[1] Lyon MF (1961). Gene Action in the X-chromosome of the Mouse (Mus musculus L.). Nature.

[2] Lyon MF (1962). Sex Chromatin and Gene Action in the Mammalian X-Chromosome. Am J Hum Genet.

[3] Graves JA (1996). Mammals that break the rules: genetics of marsupials and monotremes. Annu Rev Genet.

[4] Arthold S, Kurowski A, Wutz A (2011). Mechanistic insights into chromosome-wide silencing in X inactivation. Hum Genet.

[5] Morey C, Avner P (2011). The demoiselle of X-inactivation: 50 years old and as trendy and mesmerising as ever. PLoS Genet.

[6] Avner P, Heard E (2001). X-chromosome inactivation: counting, choice and initiation. Nat Rev Genet.

[7] Brockdorff N, Duthie SM (1998). X chromosome inactivation and the Xist gene. Cell Mol Life Sci CMLS.

[8] Goto T, Monk M (1998). Regulation of X-chromosome inactivation in development in mice and humans. Microbiol Mol Biol Rev MMBR.

[9] Heard E, Clerc P, Avner P (1997). X-chromosome inactivation in mammals. Annu Rev Genet.

[10] Willard HF (1996). X chromosome inactivation, XIST, and pursuit of the X-inactivation center. Cell.

[11] Lyon MF (1999). X-chromosome inactivation. Curr Biol CB.

[12] Loda A, Collombet S, Heard E (2022). Gene regulation in time and space during X-chromosome inactivation. Nat Rev Mol Cell Biol.

[13] Pandya-Jones A, Markaki Y, Serizay J (2020). A protein assembly mediates Xist localization and gene silencing. Nature.

[14] Chu C, Zhang QC, da Rocha ST (2015). Systematic discovery of Xist RNA binding proteins. Cell.

[15] Dossin F, Pinheiro I, Żylicz JJ (2020). SPEN integrates transcriptional and epigenetic control of X-inactivation. Nature.

[16] Arnold AP (2022). X chromosome agents of sexual differentiation. Nat Rev Endocrinol.

[17] Markaki Y, Gan Chong J, Wang Y (2021). Xist nucleates local protein gradients to propagate silencing across the X chromosome. Cell.

[18] Yang C, Chapman AG, Kelsey AD (2011). X-chromosome inactivation: molecular mechanisms from the human perspective. Hum Genet.

[19] Brockdorff N, Ashworth A, Kay GF, McCabe VM, Norris DP, Cooper PJ, Swift S, & Rastan S. (1992). The product of the mouse Xist gene is a 15 kb inactive X-specific transcript containing no conserved ORF and located in the nucleus. Cell 71(3):515–526. 10.1016/0092-8674(92)90519-i 10.1016/0092-8674(92)90519-i 1423610

[20] Loda A, Heard E (2019). Xist RNA in action: Past, present, and future. PLoS Genet.

[21] Brockdorff N (2018). Local Tandem Repeat Expansion in Xist RNA as a Model for the Functionalisation of ncRNA. Non-Coding RNA.

[22] Bousard A, Raposo AC, Żylicz JJ, et al (2019) The role of *Xist* ‐mediated Polycomb recruitment in the initiation of X‐chromosome inactivation. EMBO Rep 10.15252/embr.201948019 10.15252/embr.201948019 PMC6776897 31456285

[23] Carter AC, Xu J, Nakamoto MY (2020). Spen links RNA-mediated endogenous retrovirus silencing and X chromosome inactivation. Elife.

[24] Colognori D, Sunwoo H, Wang D (2020). Xist Repeats A and B Account for Two Distinct Phases of X Inactivation Establishment. Dev Cell.

[25] Trotman JB, Lee DM, Cherney RE (2020). Elements at the 5′ end of Xist harbor SPEN-independent transcriptional antiterminator activity. Nucleic Acids Res.

[26] Lu Z, Zhang QC, Lee B (2016). RNA Duplex Map in Living Cells Reveals Higher-Order Transcriptome Structure. Cell.

[27] Royce-Tolland ME, Andersen AA, Koyfman HR (2010). The A-repeat links ASF/SF2-dependent Xist RNA processing with random choice during X inactivation. Nat Struct Mol Biol.

[28] Wutz A, Rasmussen TP, Jaenisch R (2002). Chromosomal silencing and localization are mediated by different domains of Xist RNA. Nat Genet.

[29] Jones AN, Tikhaia E, Mourão A, Sattler M (2022). Structural effects of m6A modification of the Xist A-repeat AUCG tetraloop and its recognition by YTHDC1. Nucleic Acids Res.

[30] Sarkar MK, Gayen S, Kumar S (2015). An Xist-activating antisense RNA required for X-chromosome inactivation. Nat Commun.

[31] Pintacuda G, Wei G, Roustan C (2017). hnRNPK Recruits PCGF3/5-PRC1 to the Xist RNA B-Repeat to Establish Polycomb-Mediated Chromosomal Silencing. Mol Cell.

[32] Nakamoto MY, Lammer NC, Batey RT, Wuttke DS (2020). hnRNPK recognition of the B motif of Xist and other biological RNAs. Nucleic Acids Res.

[33] da Rocha ST, Boeva V, Escamilla-Del-Arenal M (2014). Jarid2 Is Implicated in the Initial Xist-Induced Targeting of PRC2 to the Inactive X Chromosome. Mol Cell.

[34] Colognori D, Sunwoo H, Kriz AJ (2019). Xist Deletional Analysis Reveals an Interdependency between Xist RNA and Polycomb Complexes for Spreading along the Inactive X. Mol Cell.

[35] Wei G, Almeida M, Bowness JS (2021). Xist Repeats B and C, but not Repeat A, mediate de novo recruitment of the Polycomb system in X chromosome inactivation. Dev Cell.

[36] Pandya-Jones A., Markaki Y, Serizay J, Chitiashvili T, Mancia Leon WR, Damianov A, Chronis C, Papp B, Chen CK, McKee R, Wang XJ, Chau A, Sabri S, Leonhardt H, Zheng S, Guttman M, Black DL, & Plath K. (2020). A protein assembly mediates Xist localization and gene silencing. Nature 587(7832):145–151. 10.1038/s41586-020-2703-0 10.1038/s41586-020-2703-0 PMC7644664 32908311

[37] Ridings-Figueroa R, Stewart ER, Nesterova TB (2017). The nuclear matrix protein CIZ1 facilitates localization of Xist RNA to the inactive X-chromosome territory. Genes Dev.

[38] Sunwoo H, Colognori D, Froberg JE (2017). Repeat E anchors Xist RNA to the inactive X chromosomal compartment through CDKN1A-interacting protein (CIZ1). Proc Natl Acad Sci.

[39] Smola MJ, Christy TW, Inoue K (2016). SHAPE reveals transcript-wide interactions, complex structural domains, and protein interactions across the *Xist* lncRNA in living cells. Proc Natl Acad Sci.

[40] Yue M, Ogawa A, Yamada N (2017). Xist RNA repeat E is essential for ASH2L recruitment to the inactive X and regulates histone modifications and escape gene expression. PLOS Genet.

[41] Cherney RE, Mills CA, Herring LE, Braceros AK, & Calabrese JM. (2023). A monoclonal antibody raised against human EZH2 cross-reacts with the RNA-binding protein SAFB. bioRxiv : the preprint server for biology. 10.1101/2023.04.03.535391 10.1242/bio.059955 PMC10259849 37283223

[42] Coker H, Wei G, Moindrot B (2020). The role of the Xist 5’ m6A region and RBM15 in X chromosome inactivation. Wellcome Open Res.

[43] Caparros M-L, Alexiou M, Webster Z, Brockdorff N (2002). Functional analysis of the highly conserved exon IV of XIST RNA. Cytogenet Genome Res.

[44] Lee HJ, Gopalappa R, Sunwoo H (2019). En bloc and segmental deletions of human XIST reveal X chromosome inactivation-involving RNA elements. Nucleic Acids Res.

[45] Berletch JB, Ma W, Yang F (2015). Escape from X Inactivation Varies in Mouse Tissues. PLOS Genet.

[46] Yamada N, Hasegawa Y, Yue M (2015). Xist Exon 7 Contributes to the Stable Localization of Xist RNA on the Inactive X-Chromosome. PLoS Genet.

[47] Briggs SF, Dominguez AA, Chavez SL, Reijo Pera RA (2015). Single-Cell XIST Expression in Human Preimplantation Embryos and Newly Reprogrammed Female Induced Pluripotent Stem Cells. Stem Cells Dayt Ohio.

[48] Okamoto I, Patrat C, Thépot D (2011). Eutherian mammals use diverse strategies to initiate X-chromosome inactivation during development. Nature.

[49] Liu Z, Chen M, Chen S (2018). Highly efficient RNA-guided base editing in rabbit. Nat Commun.

[50] Song Y, Yuan L, Wang Y (2016). Efficient dual sgRNA-directed large gene deletion in rabbit with CRISPR/Cas9 system. Cell Mol Life Sci CMLS.

[51] Yuan L, Yao H, Xu Y, et al (2017) CRISPR/Cas9-Mediated Mutation of αA-Crystallin Gene Induces Congenital Cataracts in Rabbits. Invest Ophthalmol Vis Sci 58:BIO34–BIO41. 10.1167/iovs.16-21287 10.1167/iovs.16-21287 28475701

[52] Felsenstein J (1985). CONFIDENCE LIMITS ON PHYLOGENIES: AN APPROACH USING THE BOOTSTRAP. Evol Int J Org Evol.

[53] Saitou N, Nei M (1987). The neighbor-joining method: a new method for reconstructing phylogenetic trees. Mol Biol Evol.

[54] Tamura K, Nei M, Kumar S (2004). Prospects for inferring very large phylogenies by using the neighbor-joining method. Proc Natl Acad Sci U S A.

[55] Madeira F, Pearce M, Tivey ARN (2022). Search and sequence analysis tools services from EMBL-EBI in 2022. Nucleic Acids Res.

[56] Bailey TL, Johnson J, Grant CE, Noble WS (2015). The MEME Suite. Nucleic Acids Res.

[57] Lorenz R, Bernhart SH, HönerZuSiederdissen C (2011). ViennaRNA Package 20. Algorithms Mol Biol AMB.

[58] Tamura K, Stecher G, Kumar S (2021). MEGA11: Molecular Evolutionary Genetics Analysis Version 11. Mol Biol Evol.

[59] Sayers EW, Beck J, Bolton EE (2021). Database resources of the National Center for Biotechnology Information. Nucleic Acids Res.

[60] Reyes A (2003). Congruent Mammalian Trees from Mitochondrial and Nuclear Genes Using Bayesian Methods. Mol Biol Evol.

[61] Murphy WJ, Pringle TH, Crider TA (2007). Using genomic data to unravel the root of the placental mammal phylogeny. Genome Res.

[62] Damas J, Corbo M, Kim J (2022). Evolution of the ancestral mammalian karyotype and syntenic regions. Proc Natl Acad Sci.

[63] Sui T, Yuan L, Liu H (2016). CRISPR/Cas9-mediated mutation of *PHEX* in rabbit recapitulates human X-linked hypophosphatemia (XLH). Hum Mol Genet.

[64] Yao B, Liang M, Liu H (2020). The minimal promoter (P1) of *Xist* is non-essential for X chromosome inactivation. RNA Biol.

[65] Yang T, Ou J, Yildirim E (2022). Xist exerts gene-specific silencing during XCI maintenance and impacts lineage-specific cell differentiation and proliferation during hematopoiesis. Nat Commun.

[66] Balaton BP, Fornes O, Wasserman WW, Brown CJ (2021). Cross-species examination of X-chromosome inactivation highlights domains of escape from silencing. Epigenetics Chromatin.

[67] Goto T, Monk M (1998). Regulation of X-Chromosome Inactivation in Development in Mice and Humans. Microbiol Mol Biol Rev.

[68] Sakata Y, Nagao K, Hoki Y (2017). Defects in dosage compensation impact global gene regulation in the mouse trophoblast. Development.

[69] Brockdorff N (2017). Polycomb complexes in X chromosome inactivation. Philos Trans R Soc B Biol Sci.

[70] Yang L, Kirby JE, Sunwoo H, Lee JT (2016). Female mice lacking Xist RNA show partial dosage compensation and survive to term. Genes Dev.

