# Supplementary material for: Unraveling the role of Xist in X chromosome inactivation: insights from rabbit model and deletion analysis of exons and repeat A
Source: Cell Mol Life Sci. 2024 Mar 29;81(1):156. doi: 10.1007/s00018-024-05151-0 (PMC10980640; doi:10.1007/s00018-024-05151-0)
Supplement: Supplementary file 1 — Supplementary file1 (PDF 49677 KB) [file 18_2024_5151_MOESM1_ESM.pdf]

SUPPLEMENTARY FIGURES AND FIGURE LEGENDS

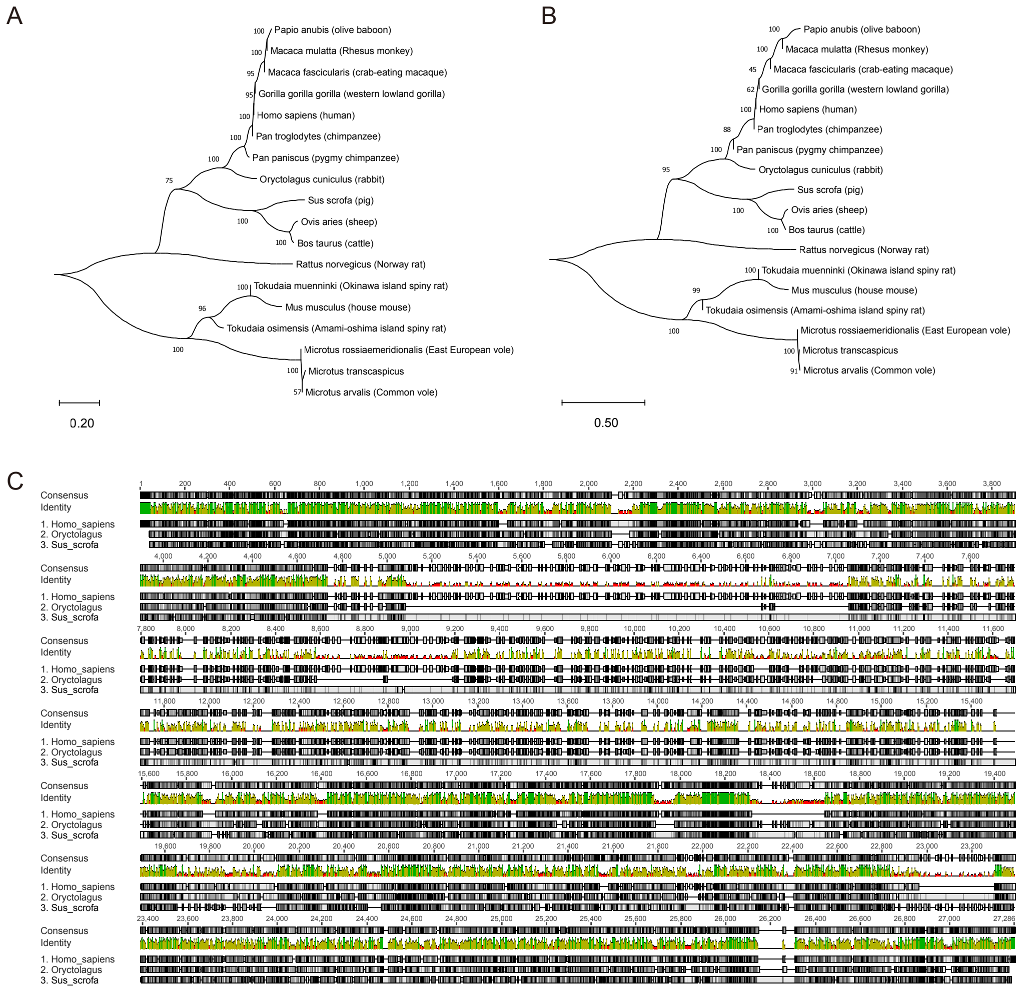

D

	Homo_sapiens	Oryctolagus	Sus_scrofa
Homo_sapiens		63.044%	50.861%
Oryctolagus	63.044%		46.101%
Sus_scrofa	50.861%	46.101%	

Matrix：%Identity（Identities: Number of bases/residues which are identical）

Figure S1. Alignment of human, rabbit, and pig *Xist* sequences

(**A**) The evolutionary history was inferred using the Neighbor-Joining method and Tamura 3-parameter model.

(**B**) The evolutionary history was inferred using the Minimum Evolution method.

(**C**) Alignment of human, rabbit, and pig *Xist* sequences.

(**D**) Genetic distance matrix of human, rabbit, and pig.

A

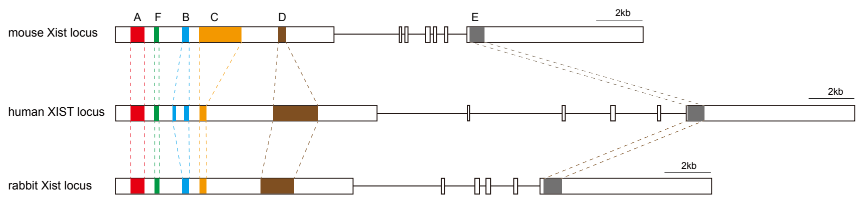

B

Table : Align Sequences Nucleotide BLAST

	mouse vs. human	rabbit vs. human
repeat A	67.48%	78.44%
repeat F	n.s.	85.71%
repeat B	n.s.	n.s.
repeat C	n.s.	76.47%
repeat D	79.12%	72.77%
repeat E	n.s.	71.23%

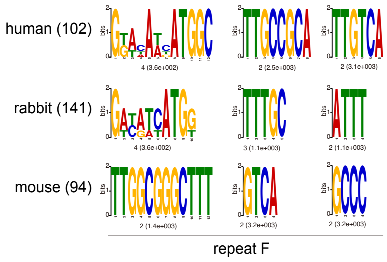

C

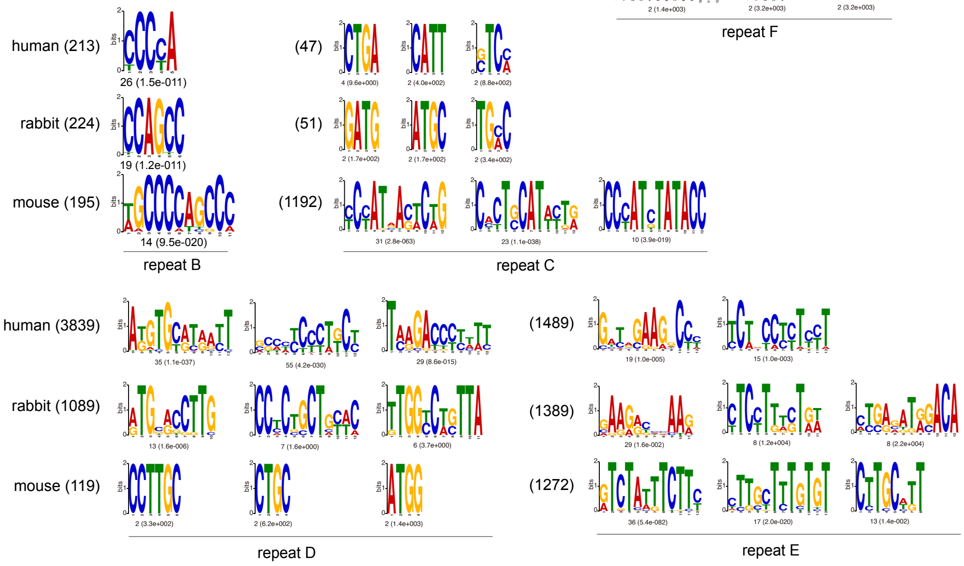

Figure S2. Characterization of *Xist*/*XIST* repeat sequences.

(**A**) Comparison of the gene structure and tandem repeats of *Xist*/*XIST* in mouse, human, and rabbit. The core repeat regions are highlighted in different colors.

(**B**) The BLAST analysis shows the nucleotide sequence homology of *Xist*/*XIST* repeat among mouse, human, and rabbit, as indicated in the table.

(**C**) Comparison of the top three motifs identified by MEME in *Xist*/*XIST* repeat. The number of sites and E-value for each motif logo are provided. The length of each repeat is mentioned in nucleotides (shown in parentheses). All *XIST/Xist* sequences used in this study are listed in Supplementary Table 4-6.

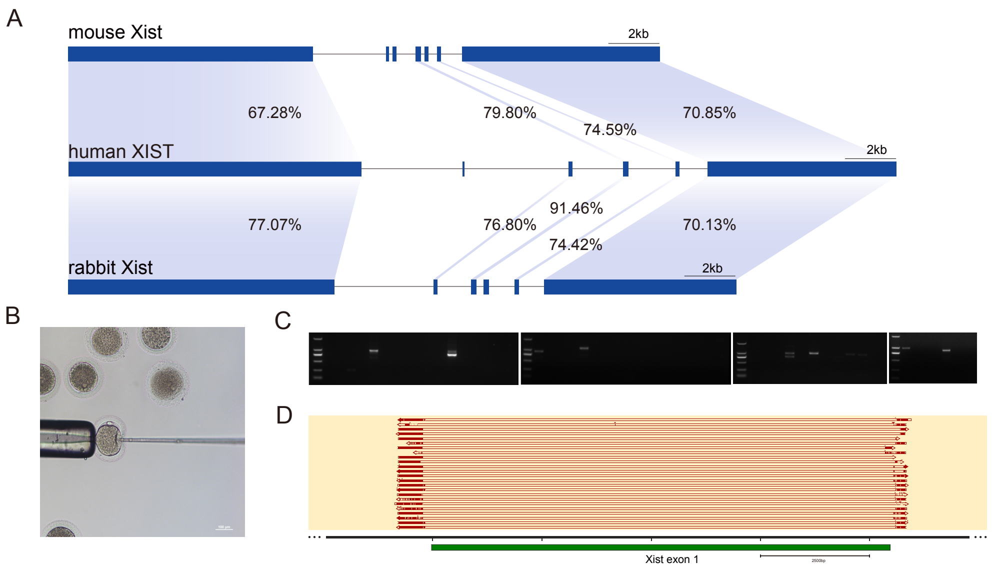

Figure S3. Characterization of the *Xist* exon 1 knock-out mutant in the embryo.

(**A**) Comparison of the gene structure and homology region of exons between mouse, rabbit, and human *Xist*/*XIST*. Exons are marked in dark blue.

(**B**) Diagram illustrating microinjection in a rabbit embryo.

(**C**) Identification of the cloned embryo through agarose gel electrophoresis using primers F3/R3.

(**D**) Characterization of the *Xist* exon 1 knock-out mutant in the cloned embryo. Scale bars: 2500bp. All sgRNA sequences are listed in Supplementary Table 1.

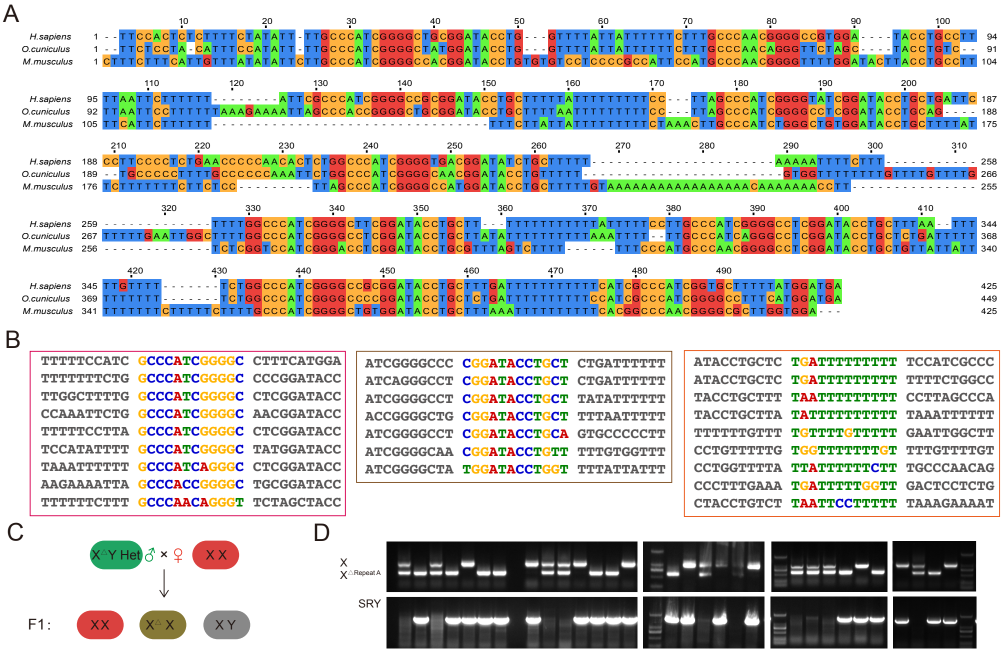

E

X△X - X Y (*Xist* repeat A F2 generation)

Pair #	Litter size	X X	X△X	X△Y	X Y
1	9	1	1	3	4
2	6	1	2	2	1
3	6	1	2	2	1
4	6	1	2	1	2
**Total**	**27**	**4**	**7**	**8**	**8**

F

X X - X△Y (*Xist* repeat A F3 generation)

Pair #	Litter size	X△X	XY
1	9	4	5
2	5	4	1
3	6	3	3
4	5	4	1
5	8	4	4
6	5	1	4
7	5	1	4
**Total**	**43**	**21**	**22**

G

X△X - X△Y (*Xist* repeat A F3 generation)

Pair #	Litter size	X△X△	X△X	X△Y	X Y
1	7	0	2	4	1
2	7	0	2	2	3
3	6	0	2	1	3
4	6	0	3	2	1
5	4	0	1	1	2
6	3	0	1	0	2
7	2	0	0	1	1
8	2	0	0	1	1
9	6	0	1	2	3
10	2	0	0	1	1
11	4	0	1	1	2
12	8	0	3	2	3
13	5	0	1	1	3
14	9	0	3	1	5
**Total**	**71**	**0**	**20**	**20**	**31**

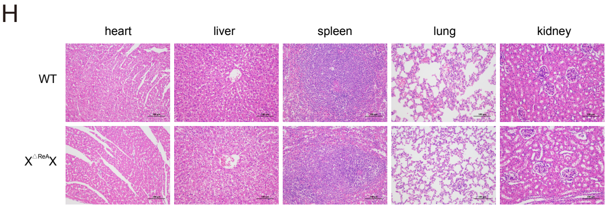

Figure S4. Alignment of tandem repeats in *Xist* repeat A.

(**A**) Multiple sequence alignment of repeat A in human, rabbit, and mouse.

(**B**) Alignment of three sets of tandem repeats in rabbit *Xist* repeat A.

(**C**) Schematic illustrating the generation of the F1 generation.

(**D**) Agarose gel electrophoresis result of PCR product in the F3 generation.

(**E**) Table of *Xist* repeat A F2 generation (X△X-X Y).

(**F**) Table of *Xist* repeat A F3 generation (X X-X△Y).

(**G**) Table of *Xist* repeat A F3 generation (X△X-X△Y).

(**H**) H&E staining for main organs from X△ReAX and control animal. Scale bars, 100um.

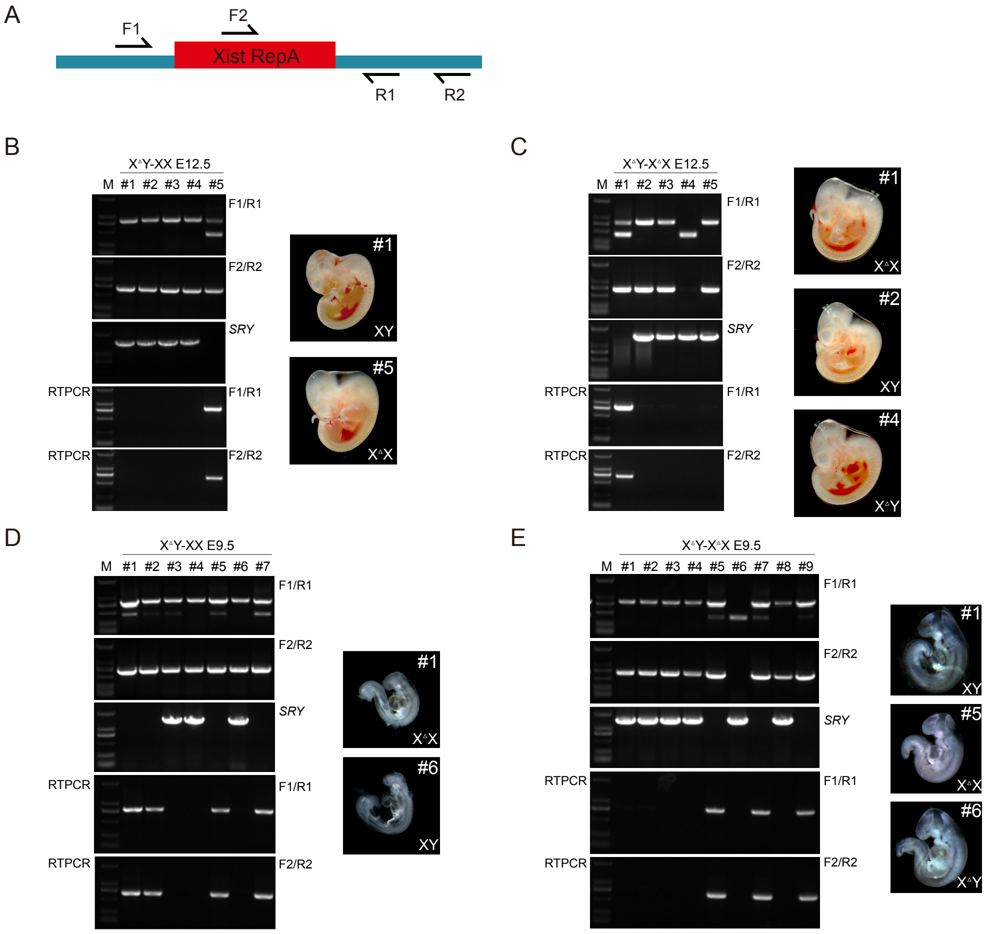

Figure S5. PCR and RT-PCR results for early embryos and their representative images.

(**A**) Position of RT-PCR primers.

(**B**) E12.5 (X X-X$^{\triangle}$Y) PCR and RT-PCR results and their representative images.

(**C**) E12.5 (X$^{\triangle}$X-X$^{\triangle}$Y) PCR and RT-PCR results and their representative images.

(**D**) E9.5 (X X-X$^{\triangle}$Y) PCR and RT-PCR results and their representative images.

(**E**) E9.5 (X$^{\triangle}$X-X$^{\triangle}$Y) PCR and RT-PCR results and their representative images.

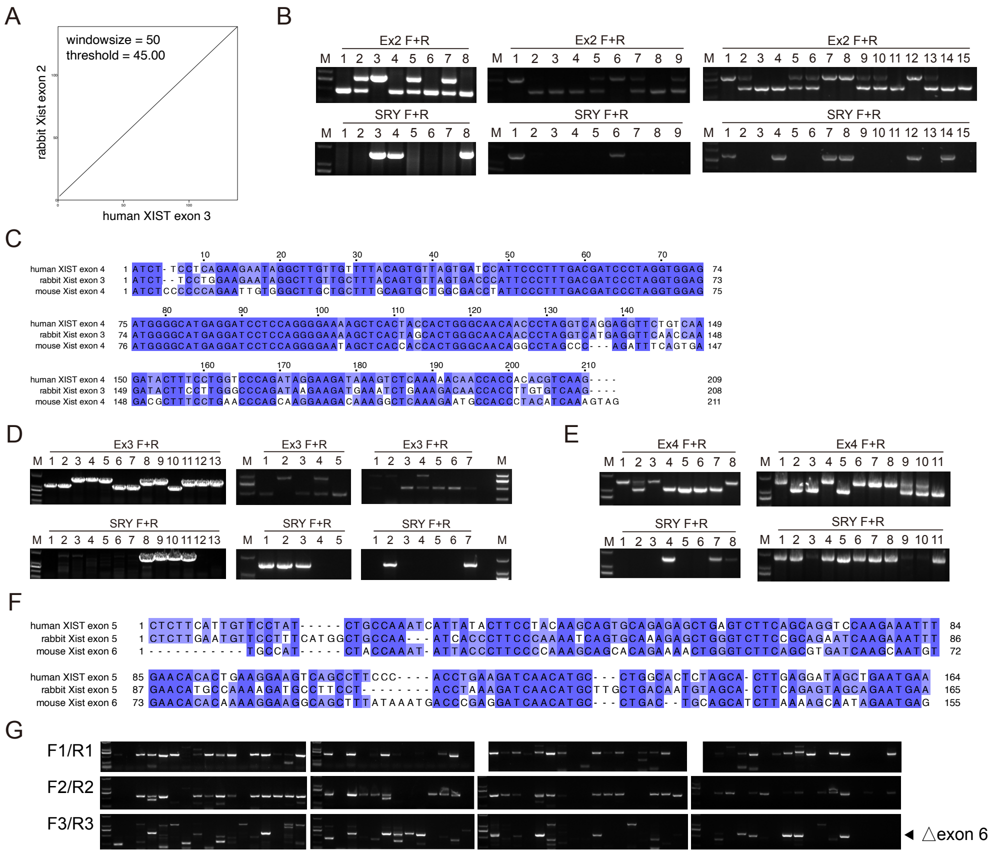

Figure S6. Sequence alignment and agarose gel electrophoresis results.

(**A**) Dot plot analysis comparing rabbit *Xist* exon 2 and human *XIST* exon 3.

(**B**) Agarose gel electrophoresis result of the PCR product (Ex2 F1 generation).

(**C**) Sequence alignment of human *XIST* exon 4, rabbit *Xist* exon 3, and mouse *Xist* exon 4. Identical bases are shown on a dark background.

(**D**) Agarose gel electrophoresis result of the PCR product (Ex3 F1 generation).

(**E**) Agarose gel electrophoresis result of the PCR product (Ex4 F1 generation).

(**F**) Sequence alignment of human *XIST* exon 5, rabbit *Xist* exon 5, and mouse *Xist* exon 6. Identical bases are shown on a dark background.

(**G**) Identification of cloned embryo by agarose gel electrophoresis using primers F1/R1, F2/R2, and F3/R3.

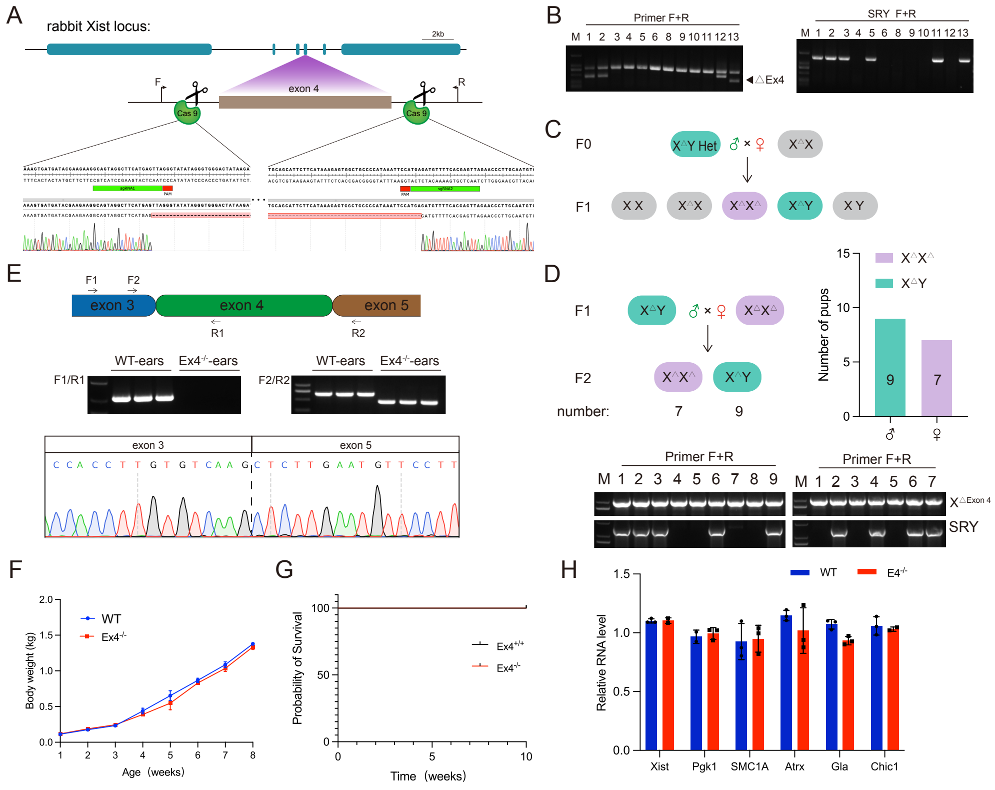

Figure S7. Viability of *Xist* exon 4 knockout rabbits.

(**A**) Sanger sequencing results and target loci confirm *Xist* exon 4 knockouts in F0 rabbits. All sgRNA sequences are listed in Supplementary Table 1.

(**B**) Agarose gel electrophoresis shows the PCR product of the F0 generation.

(**C**) Schematic representation of the generation process of the F1 generation.

(**D, up**) Breeding strategy schematic for generating the F2 generation. Genotype data and number of pups for each genotype are provided. (**Down**) Agarose gel electrophoresis result of the PCR product from the F2 generation.

(**E, up**) RT-PCR analysis of *Xist* expression. Agarose gel electrophoresis shows the PCR products. (**Down**) The RTPCR analysis results are supported by Sanger sequencing.

(**F**) Body weight comparison between KO and WT rabbits. Error bars represent mean ± SEM.

(**G**) Survival curve for KO and WT rabbits.

(**H**) The qPCR result of X-linked genes in Ex4$^{-/-}$ rabbits. Error bars represent mean ± SEM.
