# Supplementary material for: Unraveling the role of Xist in X chromosome inactivation: insights from rabbit model and deletion analysis of exons and repeat A
Source: Cell Mol Life Sci. 2024 Mar 29;81(1):156. doi: 10.1007/s00018-024-05151-0 (PMC10980640; doi:10.1007/s00018-024-05151-0)
Supplement: Supplementary file 2 — Supplementary file2 (PDF 131 KB) [file 18_2024_5151_MOESM2_ESM.pdf]

**Table S1. Summary of sgRNA and primer sequences used in this study**

| sgRNA name | sgRNA sequence | PAM |
|---|---|---|
| Xist-Ex1-sgRNA1 | CTCCTCATTTTGCTGGTTAC | TGG |
| Xist-Ex1-sgRNA2 | GTCGGGCCTTCAATCAGCGG | GGG |
| Xist-Ex1-sgRNA3 | GCCACCCATAGAAAAGTGAG | AGG |
| Xist-Ex1-sgRNA4 | TATATATATTGCAGACAAGT | TGG |
| Xist-ReA-sgRNA1 | CAAGCATTTCGAATATTCAT | GGG |
| Xist-ReA-sgRNA2 | GGTTGACTCCTCTGTTTTGG | GGG |
| Xist-ReA-sgRNA3 | CTGTGGGTTGTCGCACTATC | TGG |
| Xist-ReA-sgRNA4 | ACTTTTTGCTGCTGATCGTT | TGG |
| Xist-Ex2-sgRNA1 | ATACCTATGTCTAATGCTTC | AGG |
| Xist-Ex2-sgRNA2 | GTTGGGGTTTGGTATCCTAC | TGG |
| Xist-Ex2-sgRNA3 | GTTGCCAAGCTTACCCTTGT | GGG |
| Xist-Ex2-sgRNA4 | ACTGTCCTTAAAGACCTCTC | AGG |
| Xist-Ex3-sgRNA1 | TTCAACTATTGATGACCCAT | TGG |
| Xist-Ex3-sgRNA2 | AGTTGCTTCCCAGGTTAGTC | AGG |
| Xist-Ex4-sgRNA1 | GCAGTAGGCTTCATGAGTTA | GGG |
| Xist-Ex4-sgRNA2 | TAACTCGTGAAAACATCTCA | TGG |
| Xist-Ex5-sgRNA1 | TCACAGTTTTATAAGCTCCG | TGG |
| Xist-Ex5-sgRNA2 | GGATGTGGTAGTCCCGCTTG | AGG |
| Xist-Ex6-sgRNA1 | ACTTGTAGCAGAATGTCAAG | AGG |
| Xist-Ex6-sgRNA2 | ATTTCCATAAGATCCAGACG | AGG |
| Xist-Ex6-sgRNA3 | TTAGTCATAAAAGTGTTATC | AGG |
| Xist-Ex6-sgRNA4 | TGCTTTGGCCAAAGATACTG | AGG |

| primer name | primer sequence |
|---|---|
| Ex1-F1 | CACCATTATGCAGTTTGTGGAC |
| Ex1-R1 | GAGGTGTCAAGAAGATATCAGGAA |
| Ex1-F2 | GTGGAATTGCTGAAGCACATAG |
| Ex1-R2 | GGCTCAACAACAATCTCAGAAA |
| Ex1-F3 | TCTCTCCATCCTCTGTTTCTCT |
| Ex1-R3 | AAACCCATCTCCTTGAACTCTATC |
| ReA-F | CAGCAGCCATATTTCTACTTCTCC |
| ReA-R | AAGATGGATAGCGGGCGA |
| Ex2-F | AGCAGCAACAAGAAGTCCTATAA |
| Ex2-R | ACTGTCCTTAAAGACCTCTC |
| Ex3-F | TGATTTCTTGACTGCCACTA |
| Ex3-R | ATCCCAAGGTGTTGTAATGT |
| Ex4-F | CTTGAGGCGATGTGGAGAAT |
| Ex4-R | CTAACAAGCTCTGCTGGTAACT |
| Ex5-F | CAACTACAGGTGGGACACAAA |
| Ex5-R | GCCTCTTGACATTCTGCTACA |
| Ex6-F1 | TCAGACACTCCGGTCTACTT |
| Ex6-R1 | ATCACCAACACAGATGCTCTT |
| Ex6-F2 | ACTGCTTTGAGAACTTGCATTT |
| Ex6-R2 | CCCAGGATATGGTTGACATTCT |
| Ex6-F3 | CTATTGGATGGGATGTGGTAGTC |
| Ex6-R3 | CCCAGGATATGGTTGACATTCT |
| SRY-F | AGGCATGGAATCCACAATGA |
| SRY-R | AATGGGTAGTTTACAGGGAGATG |

**Table S2. RTPCR primer sequences**

| primer name | sequence |
|---|---|
| ReA-RTPCR-F1 | CTTCTTGACACCTCCTCCTTATTT |
| ReA-RTPCR-R1 | CGCTATCCATCTTGCTTTCCT |
| ReA-RTPCR-F2 | CTTTGCCCAACAGGGTTCTA |
| ReA-RTPCR-R2 | ACCAAGTCAGCTGATTCGATTA |
| Ex2-RTPCR-F1 | GCAGTGGGATAGCTGCCTAGT |
| Ex2-RTPCR-R1 | GAACTTGAGGTCTCTTGCAGC |
| Ex2-RTPCR-F2 | CTCTGAGAAGGATGCCGAAAG |
| Ex2-RTPCR-R2 | CTAGGGATCGTCAAAGGGAATG |
| Ex3-RTPCR-F1 | GGTCATGAGGTTCAACCAAGATA |
| Ex3-RTPCR-R1 | TACGTGGCATCCATCAATCAG |
| Ex3-RTPCR-F2 | TGCAAGTTGCCTGGAGAAA |
| Ex3-RTPCR-R2 | CTACGTGGCATCCATCAATCA |
| Ex4-RTPCR-F1 | CTGCCACATTACAACACCTTGG |
| Ex4-RTPCR-R1 | ACCCAGCTCTTTGCACTGATT |
| Ex4-RTPCR-F2 | GCAAGAGACCTCAAGTTCAGCAG |
| Ex4-RTPCR-R2 | GCTACTCTGAAGTGCTACATTGTCAGC |
| E5-RTPCR-F1 | GCACTTCAGAGTAGCAGAATGA |
| E5-RTPCR-R1 | TAGCGTATGACCCAGAGAGTAG |
| Ex5-RTPCR-F2 | CTTGGGATGTCCATGGGATATAA |
| Ex5-RTPCR-R2 | CACAAGGAAGAAAAAAGACAAAGAGAAA |

**Table S3. qRT-PCR primer sequences**

| primer name | sequence |
|---|---|
| Xist-F1 | GGGCCCAGATAAGAAGATGAAA |
| Xist-R1 | GGACATCCCAAGGTGTTGTAA |
| Xist-F2 | GTTTGCACTACTGGCTCCTT |
| Xist-R2 | TCAGTCCTCAGGTCTCACATAG |
| Gapdh-F | ATCCATTCATTGACCTCCACTAC |
| Gapdh-R | GTACTGGGCACCAGCATCAC |
| Pgk1-F | CCCTATGCCTGACAAGTATTCC |
| Pgk1-R | CACAGGCTTTCTCCACTTCT |
| Gla-F | GCGCTTCCTATGTAACCTTGA |
| Gla-R | AGTCGTCAATGCAGAGGTATTC |
| Smc1a-F | GTCGACAGATTATCGGACCATT |
| Smc1a-R | CGAAGCTGATAGCATCCATGA |
| Atrx-F | CTGGCACAGTCATCTGAAGAA |
| Atrx-R | ATCAGAGTTATTTCCAGAACCACT |
| Chic1-F | GGCTTGAGCAACAAGTTTGATA |
| Chic1-R | CATTGACTGGGAGAGCCTTT |

**Table S4. Sequence of the rabbit *Xist* locus**

**exon 1**

**exon 2**

**exon 3**

**exon 4**

**exon 5**

**exon 6**

repeat A

repeat F

repeat B

repeat C

repeat D

repeat E

TTTCTACTTCTCCCGAGGTTGGAAGCTCGCTAGCCATAGTTCTTTCTGTACTTATGGT
TCTTTCTGGAACATTTTCCAGTTCCCCCCAATCGCTTATGGGGCATTTCTTAAGAAA
AATCACCAAAATGACATAAAGGGTTTTAAGAATTCTGTACTTTTTCCTGATATCTTCTTG
ACACCTCCTCCTTATTTTTCAAGCATTTCGAATATTCATGGGCAATTTTTCATTTTTTCA
AGGATTTTTTGCCCTTTGAAATGATTTTTGGTTGACTCCTCTGTTTTGGGGGTTCAGA
TTTCTTTTCTCCTACATTTCCATATTTTGCCCATCGGGGCTATGGATACCTGGTTTTATT
ATTTTTTCTTTGCCCAACAGGGTTCTAGCTACCTGTCTTAATTCCTTTTTTAAAGAAAAT
TAGCCCACCGGGGCTGCGGATACCTGCTTTTAATTTTTTTTTCCTTAGCCCATCGGGG
CCTCGGATACCTGCAGTGCCCCCTTTTGCCCCCCAAATTCTGGCCCATCGGGGCAA
CGGATACCTGTTTTTGTGGTTTTTTTTGTTTTGTTTTGTTTTTGAATTGGCTTTTGGCC
CATCGGGGCCTCGGATACCTGCTTATATTTTTTTTTTTAAATTTTTTGCCCATCAGGGC
CTCGGATACCTGCTCTGATTTTTTTTTTTTTCTGGCCCATCGGGGCCCCGGATACCTG
CTCTGATTTTTTTTTTCCATCGCCCATCGGGGCCTTTCATGGATGAAAATGTGTTGGT
TCTGTGGGTTGTCGCACTATCTGGAATATCTACACTTTTTGCTGCTGATCGTTTGGTG
GTGTGTGAGTGAACCTACGGCTTTGGCAGGGGAATGACTTTGCAGTTAAGCTAAGG
GGGTGTTCAGGCTTTGGAGGAAAAATATCCGCCATTTTAGAGTTGCCTCATAATTTGG
CTGACTAGTCAGTTGTGTTAAATCTAAATTAGGAAAGCAAGATGGATAGCGGGCGAG
GCAGAGGTAGTCATGTGCATTGCACGAGCTAAAGGTAATCGAATCAGCTGACTTGGT
GCTTGTTAGCAGCTTTGCATGAATTGTGGTACTGGGATGCCGGAAAACAAGATTGTC
CTGCAGCAGTACGAGGCGTTAAGTTGAAAATGAGACAGGACTTAAAAGTGCTGGGCT
CAAAATGGCGCTTTTGCCATTGCAGCGTTTCTCAGCATGGCTCCCTGTGCTTTGTTAA
ATCGTCTAAAATGGCGGATTCAGTTCGGCCGCAACGTTAAAGTGGCGGGAAGGCCA
CATCATGATGGGTGGGGTCTTTTTTTGTAATGCGCAGCATGATGGTGGCAGAGTTCG

GTCACACAGTGAAAGATGGCGGCTGAAACCCTTGCCGCAGTGTAAAATATGGCGGG
CGTCTTTGTCTTTGCCGTGTGCATTTCGTGGCGGGTTTTGCCGCAGGGACAATATGG
CTGACGATATCATGTGGTTATCATGGAGATTTGTCACGTGGACGTCATGGCGCGTTTT
TGCTCGGTAAATTGGCGGGCTTTGCGCAAGGTGGACCTACGGCATTGTTAAAGATGG
CGGGCGTTGCCGCATTTTGCCGGTCAAAGCAGGAGAAAACCGGGTGCTGGATTGC
CGCATGACTTAACCAATCAGAAATGAGGGTGGATTAGGCACAGCCAATTAGCATGGA
GGATGGAATTAGACTGTCGATGCACTGTCCAGTTACTGAGCGAGGATGTGGGTGAGT
TAGCATGGCTCTTTGCAGCTTTCTTTTTTCAGCCAGTCAGGACGAAGAAGTGGAGCG
GCCATGTAGTGTGCCCCTCCCAGTGGGTGGTATTAAAGTTCTTTCCAAGGTCTTTTCA
AGGACATTTGGCCTTTCTACCTCCCTTCCCTCTTGTTTGTCCTCTCCTCTCCAGACAT
GGTCTCTTGCAGTGCTGGATATCTGGCTATGTGGGCTGAACCCCACCCACATTCCTC
TGTATTGATGCAATGGCCTTTGGCTAATCTACTCCCGCCCTGGCCTGTGGTCCTCCC
TGCACTGTGGCCACCGGCAGTGCTCCAGGCCTGCCTGGTGTGGACATGGTGGTGA
GCTGTGGCAAGGACCAGAATGGATCACAGATGATCGAAGGGAAATCCTGCCTTTCTC
AGGAGGAACATCTACCCCGTGGCTAATGCTGGGGTCGGATTTTGATTTATATTATTTTT
TTGGATGTCAGTCTTAATAGTCTGACTATGTGGTTTGCTAGTGTTCCAATTTAAGTCTT
AAGTGACTAGTATAGAAATGTATTTTGTGACTTTTTTAATTTGTAGAATTCATTTTAGTTG
CACTTAATGAGCTTTCATTTTGCATTCCATAGAATTCCTTAAATTTCTCTATAAATTCATA
AACTAGTTTTGGTAGTAGATAGTCAAATTCAAATCATCTGACATTTAAAAACTTTTATTT
CTGATTATGTTCTTTGAAATTGGCTTAACAAACTACTTCCCTTCGAATCTGTCAAGAAT
TTTGTAGCACAGAAACAGTTAATTAAAAAACTTCATGACCAAGAATAATAAAGTATTTGA
AGGTCTTCACACCCACCCAGGCCTTGCTTTATTCCTATACTTGATGCTGCACTAATTG
ATTAATGACCTACCTACCTATCAGGCAGGAAACTTGAATTTCTGTGGTCTTGTGCCCT
CTAATCAGACACATTGTATTGGAGTATTAATGCCTACATTTCAATTTTGTATGGTTTCCT
GCCTCTCCAACCTTGCTCACAGCAACCCCCAAGGGTAGCCCGAGCTATGTCCATGC
CCCAGCCCCAGCCCAGCCCCAGCCCCAGCCCCAGCCCCGTCCCCAGCCCAGTCC
CCAGCCCAGTCCCCAGGCCCAATCCCAGCCCCAGCCCCAGCCCCTGCCCCGACCC
CAGTCCCAGCCCCGGCCCCGGCCCCGGCCCCGGCCCCGGCCCCAGCCCCAGCCC
CCAGCCCCCAGCCCCAGCCCCAGCCCAGAGCTCCCCACTCCAAAACCCAGCCCTA
ACCTCAGTCTTAGCCACTCAGCCAAAGCTTGGCTGCTCCCTAGACCCACTCCAATTT
TCATTTGTTGAGCACTCAATAAGTTGTCCGTTGCTCATCTTATTTTACTGGCATTGATG
ATCTTAAATGCTGACCATCTGAGTTGCCATTTTCAGTTAATGCATACAATTCCTTTTGTC
CATGGTCCTATGTTAAAGACTTCCTGTACTCTGTATGTTAATGTTAAGAAATGTTAACT
GAGAACTTCATATGAGGGAATTATTGCCCATAATTCGAGTTACTCATCAACGTCTCAAT
CCCTTGCCTTTAAGGAAGGAGAAACCTTTCCACTGTCATTGCTGCAGTAGTTGAAGT
CCCGGTTTTCTGAGAAGTGCTCAGTGGTCTTTTCTCATGTACTGTGTTGACCACCTAA
AGCTCTGCATTGAATGAAACTCTAATTTTCCATAATCCTACCCATTGGATTAGATGTTAG
TCCAAACCGCATTTGTATTCAGGGGGAGCAAACAACCCAAGTGTTTTGTGGAACAAT
TAATGTGCTCAATCCCAGGTGTCATAAACTTATCCATTAAACAAAGATACTCTACTGCC
CATCTTGCCCTGCATTTGGAAAGTCCTGCTGTTCTTGAGCAGTTAAAGTACAGTAATT
GTTCATTGTCTTGCCCATTAATCATGAGGACCTCCAATTCTCTTTGTATTTGCCTTGAG
GCAGTGCTAATCACCTGAGACTAGGGTTTTTTCCTCTTCCCAGTAACAAACATCACAA

TTGTCCATATTCCTGTTCTTTACAAATAAGGGCATTTGCAACATTTTTGCACCACAGCA
GAAGTGCTCATTCTCAAAGTCCCTTCTCTTGAAATATGGATGAATACAACTACAGTTGT
ATATTGTCCTAACAAAAGAAACCCACCTATGCTCTTTGCTCCTAGGTTGTGCAACTTG
CCAACCACTTTGCATTAATATGTAAAACCTTTCTTTCCCATGGCCACATCCTTTAATGTA
GGATGTCCTCTACCTCTATACTATATTGCACTGAGGAGTTGACTACCCGAGATCCCTT
CTCTTAAACAGTTGAAATACATTAATTGCATATCATGTGTGTCTTACACAACAAAAATGT
ATTTGCACCCCATTGTATCTTTTGGGTGTTGTCACTAGGGGGTGCACATTATTCAAGA
ACCCAGCTCTTGGACAGTTAATTTGCAGTTACAGTTGTCCAGAGTGCTATCCACTGAA
GAAGGGCCAATATATCCTGTGTGCATTCTATAATAGTGGGATGATCACCCAAGGTTACT
TTCCTTGAGTTGATGATGCACGTAATTGCATTTGTCTATGATCTTGTGCATTGGCCTCA
TCCACTCCCTTCATTCTGGGAGACCTTTCCTACTCAAGATCTGCACTAGGACAGTTGA
TATGCGCAATTTTAATTTATTAGAATTGAGTCTTTTTAAAAGTCAAAATCCCTGCCACTT
TCTTTGCTTTACATCAAGAGGGTGCTTGATTACCTGAGGCCCCTTGTCTTGATATTGC
TGATATCTCCTGTTATTCTGGGATCCCATCCCCTCAGGCTCCATTTGCATTGCCTTAG
GGGATACTTGGGACTATAAATGTGCATATTGTGATACAAAATAACCCTAGGGTCCCATC
CCTTCACATCTCCTTAGCATAACAACCAGAATGCTGGCTAGCTATGGCTGCCTTTCCT
ATACCATTGGAATGCGCACAATTAATAGCAATCTATTCACCCTCAAATTAGAGCAGGAT
ACCATCTTCTTGCCTAATTTGCATTACTGCAGAGGCTAAGATTTACTCTTTTGAATTGT
TAATCTTATACTTTCAGTCCTGTATTCCTTACCTGTATACCAGGTAAGGACTCCACCCA
CTCTATTTGCATTACAGCAGGAGGTACTTAAAGCCACAGTGCACAATCTTAATTGTTCT
CAACACACACTCAGTTGTCCAGAACTGTCTTTTAGATCAGGACTCCTGTACTTATCAA
AGCAGAGAATGATAACCGACATAAAACCCTCCTTTTGGGTCTGTAGTTGTATATGATTG
CATCTGTCAGTGATCCTTCAAGTTAGACTTGGGACTCTGACTTTTCACACCGTCTTTG
CTTTATTGCATGAGACAGTGTTCACTTAAGGCCCCTTTATTAAACTGTTAATGTAGTTG
CATAATGACAACTACATTAGGATTCTTCCCCTTCAAATTCCCAAAAAAAAGTACTATAG
GAATGCTGACCACTGATGCTTTAATTTGTGGCCTACTAATATAAGTGATGGTATTTGTC
CTTGAGTGCTTATATGCACCAGAGAAGGAAATCCCTCCTCTCCCGTCACCAATTTTAC
CTTGTCTTCAAGCAGGGATGCTGATTCCATAAAATTCTTATGTTTATATAGAAATGGGA
CACAGCTGTAAGTGTGCACAGCCAAGCACCTTGGACATTAACGTGTATAACTGCACG
TGGCTCATCCCATTTAAATAAGATCCTACTATGAGGCTCCTTTTGCAGTACAGCAGAG
GTGCTGATCACCAAGGCCTATATTGGCCTGTTAATGTGGGTGACTGTATTTATCCAGT
TTCTTGTATACTAGACAAGGAAGCCTCCCTTGCTCACCCCTCCCCCACCTTTCCTTTC
CTTTCTAGCACAAATGCCCACTCCATAAATCTTACATTGGGAGAACAAGGTGCACAAT
TGTAAAGAAGCACAGCCATGCACCTTGGATGTTAACGTGCATAACTTCACGTGGCTC
ATCCATTTGAATAAGATCCTACTCCTTTTACAATACAGAAGGATGCTGATCACCAAGGC
CCCACATGTTGGCCTATTACACGGGTGATTATATTTGTTCAATTTTCTGCTTTCTAAAG
ATTTACTTATTTATTTGAAAGGCAGAATTACAGAGAGAGAGATCTTCCATCTGCTGGCT
CACTCCCTAAAAGGCTGCAACAACCTGGCCTAGGCCAGGCTGAAAGCAGGAGCCAG
GAGCTTCCTCTGGGTCTCCCACATTAGTGCAGAGGCCCAGGGACCTGGGACATCTG
CTGCTTTGCCAGGCATACCAGCAGGAGTTGGAGGGGAAGTGGAGCAGCCGGGTCT
TGAACTGGCACCTATATGGAATGCTGGCACTACAGGCTGTGGCTCTAACCTGCTGTG
CCACAGTGCTGGCCCCCAGTTTCCTGCTTTCTCTAGACAAGAAAGCCTCCCCTGCTC

CATCGCTAACCCCATCTTCCTCTCCCTTGCAGCAGAGGTGTCCATTCCATATGACCAT
TACAGTCAAGGTGCACAATTGTAGTGACCACAACCATGTACCGTGGACATTAATGTAC
ATAAGTGCACAAGGCTCATCCCATTTTGTGGTACAGCAAGAATGCTGATCACTAAGGA
ACCTATTATTGGCCTGTTAAGTGGTTGATTATATTCGTCCAGTTTCCTGTGTGCCAGAA
AAGGCATTTCCCACTGCTCCTCCTGGCCCTCATCTTCCTTTCACTTCCAGTTCTGATG
CCTAATCCATAAGACATTACATGGGAACAGTCAAGGTGCACAATTGTAAGTGACCAAA
GCCATGGATCTTGGATATCAACGTGCATAACTAACTACATGCGGCTTGTCCCATTTTG
ATTAAGCTCCTACTCTCAGAACCCTTTTGAAGTATAGCAGGGGTGCTGAGAAGTAAG
GCCTGTAACCTTGGCCTGTTATGTGGGTGATTATATTTGTCTGAGTTCCCATGTATTAG
ACGAGGAAACCTACTTCCTTCCACCCTCTGCTGCTCCTAGCGTTTTCCTTCCAGAAG
AAATGTCCACTCCATAAGATCATTACATGTGGGCAGTCTAGTTTCACAATTATAAATGC
CACGACCATGCACCTTGGACATTAATGTTCATAACTGCATGTGGCTCATCCTATTTAAA
TAAGATCCTACTCTCAGGCCCCATATGCAGTACAGCAGGGGTGCTGATTACCAAGGC
CCATATTCTTGGTCTGTTAAATGGGTGATTATAATCTGTGTGCTAGACAAGAAAGTCTT
TCTTGCTCCAACCCCAACCTAATCTTCCTTTCCATATCAGCAAAGATACCTACTCCATA
AAACCATTGAATTTGGACAGTGAAGGTGCACAATTTTAAGTGACCACAATGCACCTTG
GACATTGATGTGTGTAACTGCGTGTGGCTTGTCCTATTTAGATGAGATCCTACTCAGA
CCCCTTTTGCAGTACAGCCGGGGTGCTGATCATCAAGGCCCATGTTCTTGGCCTGTT
ATGTGGGTGACTATATATGAGTTCCCATGTACTTGTTCATATTCCTGTACAGTAGGTAAT
GCTAAGCCCACCTACTACTTTAATATCTTAATGTTTGTACAAAATAAATGGTTTTTACTT
GACCACAACTGGATGCCTTTGACATTAATGTATGTAATCATGACATTGTTCATCCTATTT
GACCAAGATCCTATCCTCTTGCTCTCCATTTACAATGTACCAGGGATGCTGACCTCTA
AGCTCCCTTTTCTTGGTTTGTTAATGCACATAATATATTTGTCTGGGTTCTTGTGCATTA
AACAAGGATAGCCCACCTCTGAGTTCCTATGCCTTTTAATAGGTGGTTTCCACTGACT
CAGACCTTTGAATTTGGACAATTAATATGCATAATTGCAGTTGTCCACATCCCCATTAC
TTCCAAGACCAATGTACCTCCTTTACATTACTGCAGGGGATACTGCCCCCCCCCCC
CAGATCCCATCTAGTGGATCATGAATGTACATAATTGAAATTTTCTTTTGTCTTTGTCAG
TAGCATATAAAGTCTTATCCCCTCACTTTTCCTTAGTAGTACAGCAAGATGTACTGCTC
AACCAAGGCCCTCTTCGTTTGGCGGGAGTGTTAAGTGCAATTCCATTTTCTCCTGTA
CCCTTCACTAGATAAGGGTCCCATCAACTTCCTTAGCATTTTCAGCAGGTAGTGCTCA
CTACAAAAGAATTTTTATTTGGAAAGCTCACATACATGAACAGTTTGCTAAGTATGTCC
TATACCAGAACTCCTGTAATCTCCCTGCTTTACAGCAGGGGGTGCTATGCACTCAGTG
CCTTGTCTTTTGGATTTAATGTGCATTAATGCAATTGCTCCTCTCTTAGGGTATCACTT
CCTTATACTCCTTTTTTTTTGAATCAGGGGCATATTAACTATGTATTGGCCCCTTTTCCA
TGAATATTAATGTACATAATTGGTCTGTGGACCTATGTGGTAGAAAAGAACCCTATGCT
CCTACCACTGCCTTGTGATTGCAGCTGAGGAGAGCTAACCATAACCTCAGCTCTTGG
GCAATTAATATGCAACAATAACACTTATTGAGAATACGGATTGTCAGATGGAAACCCTG
TACCCTCTTCATTTGTAACAGGGGTGCTGACCAACTGTGACACCTTGAACTATTAACT
TGAATATTTGCACTTGGGCTCCCCCTTTCTGTTAGACTGGAGTAATACACCCTTACATA
ACCTGTGCATTACTTCAGGGTGTGCTGGCCACTCCAAACTTCTCCTTGGACTGTTCA
TGGGCACAATGACAATCAATGGTTCTTTACCTCTTTGAAACCTCTCCTGGGACTGTTA
ATAGGCACAATGGCAATTATCAATGGTTTTTTTCTCTCCCTGGCATTGTTAAACAACAC

TTTCTTTCCCATGGTGCAATAAATTGTAGTATGCCATGCATTTGTCCATCAAAGAACAA
CCTATATTTCCATTCCCAGGTAGCCATGGTCTCACCATCTATGCAAGGTTTTCCCTCTC
CATTGCCCCTTGTGTGCCCTTGGACCCTTCTCATGGACTATTAATGCTCACAGTCCAG
CCATTTGAAAAGATTGTGCTCCTTCTGTCCCATCTGCCCCCACCTGGTCCATTTTGCC
TCTTTTGGTATGTAATGTTGACTGAACAAGGCCCTCTCTCTTCGACTTAAAATGTTAAT
CCCAAATGCCACTGTTTTACCCACTTTGCATTGCTGTAGTGGCTCAGTTGCACTTTCC
TTGGTCCCGCCCATTAGACATGGACCCCTCCCACTTACTTTGCATTGCTTCTGAGCA
CTGCTGACTACCCAAAGCCCTTTCTGTGTTATTAATGAATGAACACAATACTGATTGTC
CCACTTTGCAGCCTGTCTTTAAGATCTCTCTACCACTTTGATGTGTTTGTGCAGCATT
CATTAACAAAAGCAGGCCTGGAACTATGTGGATGAACCTTCATTTTTTTCCTGCAATT
GCTAATTATTCTGATTTCAATTATTGTCACAATCTGGGTACAGGAGTCATTCCTTCCCA
GCTCTATTATTGCTTTATTGTGCAAAATATTTACAGCACATCAGGCTCAGTGAGCTGAA
ATACCATCCTGTATTTTGGGTATTTAACATATAGTGATCAGTTGATTGGAGGAGAGTTG
TGATTTTCTTTGCATTCTTCTTCCAGAGAAGATTGCCTGGTTAAGAATCTTTCTTTTGT
GTATTGCTGTGGTGCTGTGCCGACTGCTAGGATACAACCAGAAGCGTTGTTAATTGC
TTGTTTTTTAAGAAAGACATCTGGATTACAAGGTGGAATTGATAGGCTGGTCATTAATT
TTTTTAAGCTGAAAAGTCCATTTATACTATGTACTTAATGATCAGTGTCTCTCATTTTAC
CGAGTGTGGTGGGTCTGTGGATAGACCACTGTGACTCTTGTATTATAATTATGGCATT
ACCAAAAGGGTTCTGGAGTGGAGTGGACCTCTTAAGATCAGTATCTTTGGGCTGTAC
TACCATTTTAAAATCAATCCTTGTTTGGTTTTTACCACCATTTGCTTTTAGAAAAATGAT
CTAAATGTTCTAATCCTTCAATTTCTTCATCTGGAGCACCAGCCTCTAATTATTTCAAGA
AGATGGCTATAAAAATGATTAAATGAGAGAACATAATGCTGAGGTGCTTCTGAAAACC
ATAGGTCACCATTTAGTTCTGCTAATAGCTTGAAGCATCACACTGAAGTGAGGACTTA
ACCATAGAAATGATGGGATCAGTTTCCCCATTTTATAAGAAAAATAAGCCATTACCTCA
TCATTCTTCTGAACATAAATCTCAGCAGTGGGATAGCTGCCTAGTAAAAAGGAGTAATA
TCCCGGCCTCTAGTGTACAGTGTCTTATGCCAAAGGAGTATTTAATGTGGAATTGCTG
AAGCACATAGCTAGTCATCACAACAGCAGTTCTTTTTAACCACTGAAAAAGGATACTAT
GACTCTGAGAAGGATGCCGAAAGATCAGCCCAGCCCAGGGTGCAGTTTGCACTACT
GGCTCCTTGGACAGCTGCAAGAAGAGTCTCTGGCTCTTTAAGATTCTGGTCATTCCC
AACTATGTGAGACCTGAGGACTGAGAGCAGCTGCAAGAGACCTCAAGTTCAGCAGA
TCTTTACTTTTGAGACTCCAGACCAGTTCCAACTATTCTAAGTGGATGGCTTGCAAGT
TGCCTGGAGAAAAAGATCTTCCTGGAAGAATAGGCTTGTTGCTTTACAGTGTTAGTGA
CCCATTCCCTTTGACGATCCCTAGGTGGAGATGGGGCATGAGGATCCTCCAGGGGA
AAAGCTCACTAGCACTGGGCAACAACCCTAGGTCATGAGGTTCAACCAAGATACTTC
CTTGGGCCCAGATAAGAAGATGAAATCTGAAAGACAACCACCTTGTGTCAAGGAGAA
CATGGACAACAACTGCCACATTACAACACCTTGGGATGTCCATGGGATATAACACACA
ACAAGCTATTTGAGAAGATAAATAAGAGTTGTAGCTTTTTGCTCTGTGCCTGCTGATT
GATGGATGCCACGTAGCTACGAATTAAAACATGGGAAATGACACAGTTTTCTATCATT
GGGCAAGAACTAAAGGATGAGTTCCCTGTGTCCTCTAGCTCTTGAATGTTCCTTTCAT
GGCTGCCAAATCACCCTTCCCAAAATCAGTGCAAAGAGCTGGGTCTTCCGCAGAATC
AAGAAATTTGAACATGCCAAAAGATGCCTTCCTACCTAAAGATCAACATGCTTGCTGA
CAATGTAGCACTTCAGAGTAGCAGAATGAATGTGTATTTCTCTTTGTCTTTTTTCTTCC

TTGTGCGGCTTTGCTCTTCTCTAAAGTGATTGTTATCCATTTCCATGTTTCTCTTGCTA
ATTTCTTCTATGTGTGCTTTTGCTTCATTTTCTCTTTTTGTCCCTAAGTGTGATCTCTGC
CTTGTATTTGTTGTCTCTCTCTAGTTTGTCCACTTTGTTGCTGTTAACTCTTTGCTCTC
CTACATCTGGCTCTTCTTTCACTATGTCTTCCTTTTGTTTCTACTCTCTGGGTCATACG
CTATGTGCTTTTTTGCCACTTTCTTCTTCTACCTATCTTTCTTTGTCTCTTTGTGACCCT
TCCACTTCGTGTTGGCTTGATGTTTCATGTTTCTCTGATTCTGAGCTCCTTTCTGATGT
TTCTCCTCCTTTTCTTGCATTTCTCTTTTCTACCTTCTCTTTTTGCCCCTCTTGGGCTA
TTTTCTCTCTTTCCTCCTTTGTGTGCCTAAGTGTCTCTTTGCTATTTGTAATTGTCTACC
TCAGCATCAATCTCTGCTTATTTGTGTTTCTTCTCTGCTTTTCCCCTCTCTATTAACCTT
TGACTCTTTCAGCCTCTATGTTTGCCTCTCTTTGATTTTTATGTAATTTCTCCTTGGATC
TGTCTTTGTGTATGTGGGCATGTGTGCATATATGTGCATGTCATGTGTGATTGAGGGG
GTTCCTAACCCCTTCCCAATAGGTGCAGAATGTCAGCTATCAAAATGAACAGTGTAGA
AGCTGTTTCTTATGCCAAGTTACCTAATGAGATGATCAAGACCAAAACAGCACCCTAA
GATCAGAACTGAAGTAAAACCCAAGACCACAAATGGACAGAAGGTGGAAGGTGCAT
GATGGATAAAAGACGCAAAGTGAAAGACAGATGGTTTAGACCTTGAACCTTGAGGAC
TAAGGTTGAAAATACAAGACCCCAAAAAAGATGAGACCCTCAGGCCTGAAACCAAAC
AGAAACTTTGAAAACTAACATGTACAGAAAGTGGTCTGAGTTGGACAGAAGGCCAAA
GACCCAGGGTGGAGGCTGGAGGCCCAAGAGCTGGAATGGACACATGGGAAGCTTA
AAAACTGAGATGTATAATACAAAGCCTAAGACCAAGTTATAGTTTGAAGACCTAAGGC
CCATGGATGGAAGGCTGCAGACACAAACAACACCCAGGACTCAAGCCACCCCAGAT
GGACAGAAGACCCAAGCGAAACAGTGATCTCCTGACTAAAGGTTGGAAGCTGAAGT
TCCCTTAACCAGAGCCCAGGAGGGGAGAAACCCGGGGAAGTCCCAAGATGAGAAC
CCTAAACCCCAACTCTTTTCTATTGTTGTACCTTCTACTCTTAGATATTTTGAATTTTCT
CCTCCCAATTATCATATTGCCATTAAGCCTGCTTCTTTTGTGATGTCCTTCAGAAAGGA
ATGTGTCTTTAGAGTGGTAATATGCATGGGCCAGTCTTGAGCCAGTTTTTGTTGTAGT
TTTTAGCCATTCATTTTTTTCATTTCTCTTCATGTCTATTCCATTTGAGAGAGACAACAA
AATTCAATCAATATCTAGTCTGGATTTTGGTATTACACTCAGGAGCAAGCATCTGTATA
GGTTATATTGCATTTCTGTCTTCTTTTTTAATCTCAGAAGCCTTGGGCTGATGAGAAGA
CAAAAGCTGTTGTGGTAAAAAGAAGTGCCAGGCTATCTAGAGAAAATGTGAAGAGAT
GCTCTGGCCATTGAGAAGAATTAGACAAGAAATAAACAGATCATACCAGCCTTCTGAA
AAGCACTAGCCAACAGCACCTTCCTTTGAGCTTATCCAGATTTCTGAGATCTGGAACT
TCTAGTAATGGTTACAGATGGGTGATTGCCTGCCCAGAGTGAGGCTTACACTCCCAG
CTCATTGTAAAGGCCAGATAATTTGGTGGACCACACAAACCCTATTTCTGAGTTTAAC
ATCCACTTGTAGGTTTAAAGATGACAGAACATGAGATGATATAATGGTTTAATCCTTTG
CTTGATCACTAATTCTCCCCCTTTTGTGAAGGAAGTTGATGTAAATGATTGATAATGTG
TTTTTAAACACTGTAAAACACAAGACAAAAACAAGCAAGGCAACCTCATTAATAGCATA
TTGGCAGTCACAGACAATTAACATTTAAAAATACAGTGCCTCTTGTGAAGCATTATGTA
AATGCTCATCACACATTTTCTCTGAGGTCAGATCTTAAATATTTAGGTTTTTGGGGCCA
TAAGCTCTCAGCATGTTTTTTTTTTACACAACCTTTTTAAAAGGTAAGAACCATTCCTA
CCTTGTGAGCCAAGTGGATTTGATCTGCAGTTCAAAGTTTACCAACTTCTGTTGTAAA
TGCCATAGAATATTTCTGAATATTATTTCCTTTTCTGTTTGTGTCCTTGTGCTAGTCTCT
AAGGATGTGTGGTCATATCCTGGTGACTTGTAGAAAAGTGTGTGAATATGTTTTATAAA

CTTCTTTGAAGCACTGCCAAAAACTTAAGAGTTTCAGTATTGATGAGTGAATATTGGCT
GCCTATTCTGTGAATCCTGTGCTAAGCATTGGAGATACAAAGATTTAAAGCAGCATGA
ATAAAAAAGTGCTTTTTCAAAAGAATGTAGTACTACATAGTACTATGCAGACATAAGAC
ATTAAGTTTTGGATAAATGAAGAATTTGTATAGTTGTGTTTCTGTTGCTCTTTTGATTTT
GAGTAAAAGTTCAGTATTCTAGGCAACAGAAAGCACATGGACATGGCTTTTAAACTAT
AAACTATACTACAAACAAACGGGATTAACCTACCGTGATCCTCTACATTGTGGCAAATA
TGCTGGAGGAGTCTTCTCACAGACTAAAAGTTTCAGAAAGGAAGTGCCTATTAAAGT
GCTTTGCAAACTAAAGCACTATACAAAATATCAGTGAGCTACATGTTTATGAATGAATG
AACATTAACTACTTCTTACATACCCAGTATTGTTTTGGGCACTAAAACGCCCAAAATAA
GTTTTCCCAAGTGATTCTCTCATGTAGTAAAAATTTAGACACAAGGAATAAGATATCAA
TTGATCTAATCAATAAAGACTTTCCCGGTGAGATTGAGTTAAATTATTGCTGATATGTTT
TCCATACTAATGGAACGTAATGAATGTGAATGAGATTTCTAAACTACAAAATGCTATGTA
ATTTGAAGACTTCAGATGTTTCTGTATGAATAAAACGTTCCACAAGATAGGCATTGTTT
CTACAAAACTCTCATTTGTGTTTGAACCACAAGGAGAAAGATAGTTACACAAACTAAG
GGCTTAAATACTTACTAAAGGAATGATTCCATGTCAAATTGCTTATTGTGTGAACTCAC
TGTTACAGGCAGTGAGGAAATACAGTTTTGTATTCAATTGTGCCTGCAGCTGAGAATA
AGGCAGTACATACGTTAGCATTTTGTGAGGTCTAAAGCACCATAAAAATGTTACTATGG
AGTTTATTTCTCACCTGGTGCTGAATACTAGGTTTATAAAAATTAAATCACAGTTATTATA
CTATGCAAATAAAGCAGAAAAGTTGACTGAGGATTGAACATGCTAAATATAATATGTAA
AGTGTTCAATGTCTTATGAATGCAGAGTGGATAGTAATAATTTAATATACACCAGTCTCC
TTTGCAAGGCATTGAGGATGCAAAGATAAACCATCTTTTTATCTAAAAAAAGATTTTATT
TATTTGAAAGGCAAGGAAATAGAGATCTTCCACCCACTGGTTTATTCCCCAAATGGCC
ACACTGCTGGGTCTAGGCCAGGGCCAGGCTGAAGCCAGGAGCCAGGAGCCAGGAA
CTCCATCTGATTCTCCCACGTGGGTGGCAGGGGCCCAAGTACTTGGGCTATCTTCTG
CTGCCTTCCCAGGCACATTAGCAGGAAGCCAGATTGGAAACTGAGCAGCCGGAACT
TAAAATGGAGCTCATGTAGAATGCTAGCGTTGCAGGTGGAGGCTTAACCAGCTGTGC
CACAACGCCAGCCCCAAAGATAAAACCATCTTTATTGTGTCCTGGGTAGCAGAGGAA
TGTGTATGAAAATAATGTCATTTATAAAAGGCTATGTGTACATTAGCAAAATTTGCTAAA
TGAATACTCTTTACTATTATCCCTAGACATAGTAACTGGGGTTACAAGAATTCATTGCCA
ATATTATTGAAGCTGCAATGGAGATCTCTATGTAATTCACAAAGCCTACAGTTCTATAAA
GATAGGAATTAACTGTTGGCTTAATGAATACAGATTACTTTCTGTGAAGCTCAGAATTG
CATATCCTATTATGTAAAAATGAACTTACCATTGTGTGATCTCACAGGAGGAAATAAAAT
AATAGTTGTGAGATCTGGAGCAGAAGTTACTTAACTACTAAAAAACTTATTTGATGATG
ATGATGAAAACATGGGGAAAAAGGATTTGTGTAAGGCTGTGGTAATGCTGAATTAAGT
GAAATGATGAAATCCTGTTCATCAGAATACTTGGCTCCTTATAGAAATGATGGAAATTC
TTTTGTTAAAAATCAAAGTGTTGTCTTTCCATCCAGTTTTTGAATGCTCCATGCTGACA
ATGAAGGTGAAGTAGATTCACCATGGCGTGGCGTAGAGATGATGAAGGAGAAAAATG
AAAGGCTCAAGTTTGCTTTTTGATTCACCTGATAAACATTGTTCATGTGGAATACCCCA
TTCCAGGTCCTGAGCATAAGAGATGAGGTGATATGGACCTAGTCTAAAGCACTTAGAC
TACTGATCATAGCTGAAGATCGAAAACCATCTTTAATTTGATTTTTGGCTAAATAAATAT
ATGAGTGCCTGCTCTCTGCAAGGTGTGATGGTTGATGAATTATGTAAATCTGGTTCAG
AAGACCCATTTACTTGCCTTGGAGATCTAAGGGAAATGAAAAAAGGCAAGATTCATTC

AACATAAAACAGAGACATTTTTCTTGGTTCCAGGAAACTCCAGAGTTGCTTGCTCATT
TGTCTCCTTTCAAACCAGAACACTCCTAAAAGGATGCAGGAATCCTTCAATGTCCTTC
AATTCTGCTTTTATTATAGTTCATTAGTTTAAGATGAGAATCAAGATGAAAGGACTAGTT
TTAAAAGGTATTGGCAGACTGACCTGGCTTAGAGTCAGGAAGACACTTGAGAAACAA
CAACAAAAAAGGTGTTTCATATTTGCTATTTTTAGTAAAGATGTGTTAAATCTGAAGAAC
GTACTAAGTGCTGAGGAAATGAAGAAAAGCAAAAAGCCTAGGCCAAAATGTACTTTAT
ACCCACCAGCTTATGGTGGAACCACAAGAAAAAACGTAATAAGAGCAAATTTCATAAA
AGACTGCAGACCTTTCTTAACTGAAAAACTTCACACTGCAGCTGTGACTGTAAGACA
CAGAGAAGGAGTGATTAAATAAATAGATCTCCTTCTCTTTGGCGTGCTTTGTAGAGTG
GAAATAAAAGAGGACTTTTTTATTTTTGCATTCATACAAATATTGATGAAAATGATGCTA
AGCGCCAAATTAAATAGATTTGTTCCAAAGGCACTTAGCCTGATTTGGAGATGCAATA
AGAAAACAAATGGAGTGATACTCTGTTAAACAGAAGTATCTATTATGTCAAGCCATTGT
GGTAAATCTTAGATTAAAAAAAAAAAAAACACAAAAGATCTTGACTGAAAGACACTTG
CTTATCAATTACCTCATTGATTAAAATATAGGAGGTAGTTTAATGTTCATTCATTTTTTTC
ACTTGGTCATTCTACAAATATATCTGGGTGCCTACTAGATAAAGAATCCTTTGGTAGGC
TCTCAGTTGCCTCAGAGACATTACAAACTTGCATTCCTGCCTAGAGCATTTCTAAAAC
TTTGCTTTTGACCTGTTTCCTGATTACAGGCATGATTTTGTCCCTAAACAACATTGTGT
GGTTTGATTCCTACTCCATATCTCTTCCTCCGCTCTGGCCACTGCAATCAGCAGGTAG
CTGGGTTTTGTAGAGAACTTGCTCCTTGAGTTGCAGGAACTCTTCTTGTAATGGACG
GCTCATCCCTCATGAAAAATGACTAAGACCACTGTTTGCCAGAAGGAGGGATGCCAA
CCAACTAATTCCCAAATCTCAGTCTCACTGGGGAACCACCCATACCTGAGCCACAGT
TGGTATTGAAAAGCATTTACAGTTGTGGTCTATTTGCAATTCTTTGACCCTTTATGTGC
TGACACATGGATCTATTTCAGAAACAATCCTCAGCACATAATAAAATGGAGATGGAGA
CATGATTTCTTTTACAACAGCTTCATAATATATACCATAGAAATGTTCTTATCATCAAAAG
AATGTCAGTGGATGAAAATAGCTTAAAACTTAAGAAAGCTTGCTTAAATCTTAAGTTCC
ATAAAATACAAATTGGAAATGACAGAACAAAAAAAAGTAAAGTACTCTAAGTCTATGCC
AGACTAGGATTTGACCTTTTCTATTTTTAAATCACTCACAGAGGGTGGGACAGGAGG
GAGAGTGAAGGAAAGGTCAAATCTGTTCTAAGGGCAGTTGCCCTTTGTTCTGGATTC
TGTATTGAGAGCATTACTAGCTCCAGTTATAACTTGGGATTGCTTTTTAAAAGTTTAATG
CAGTTCCAATAACTGATTATTGTTGACATCAGGTCCTAATTTGCTGTATGCCTTTTGAG
AAAAATTTTTGTCTGAATGGCTATCAACTAAGCCCTGGTTCAGAAAGGAATGGAATTTA
TTAATTGAACCAATGTGACCCACTTTCATAAGAGTTCTTTAAAGCTGAAGCTAACATTT
GCTGCAGAGGAAAAAGACTATTGTTCATTGTAGTTTGTAAATTCTTTCAAAGTATCTGA
ACTAGTTCACGTGGCAAGCCCACAATAACATCCGTAAATGGAGAAGGGAATAATGGC
AAAGTATAATTGGTGAGTGGAACAATGTAAAAGGGGATTTGGGGTAATACTTGTTAGT
TTCAACTTTGTACATTTTTATTAATATTAGACTTTTTAATGTATCAGCTAGACACAGCACC
CTTTCTTCAAGCAGGGTTGGGGGGTACATTTTTGGAAAGTGGTAAGGAGAAAGTAAC
TGAAAGCCTTCCTTTCACAGTTTCTGGCATCACTATCACTGCCAATTAGACAAGAATA
AGAGAACATGCTATCATCTACTTTAGTCGCATATGTGAAGTTGTAAAGGAAACTCCTTC
ATCTCTTCGTTTCTGCCTCAAAGAATTCTATGCCAAAATGCTAAGATAAATGGAAGGAA
GTTGGACTTGTGAACTCATGTAAAAAACTGACTGTTGATTCTTTAGTGTTTTGAAGATC
CAAAAATGTTGCTCAGCATGGGTGACCAACAAAAAGCAATCTGAAGCTATCCCCCAC

ATCGTGACAGAAAAATTGGGGAGTTTGTTGTGTACTGTAAAATAAAATGTACTGCTTT
GAGAACTTGCA

**Table S5. Sequence of the human *XIST* locus**

**exon 1**

**exon 2**

**exon 3**

**exon 4**

**exon 5**

**exon 6**

repeat A

repeat F

repeat B

repeat C

repeat D

repeat E

CCTTCAGTTCTTAAAGCGCTGCAATTCGCTGCTGCAGCCATATTTCTTACTCTCTCGG
GGCTGGAAGCTTCCTGACTGAAGATCTCTCTGCACTTGGGGTTCTTTCTAGAACATT
TTCTAGTCCCCCAACACCCTTTATGGCGTATTTCTTTAAAAAAATCACCTAAATTCCATA
AAATATTTTTTTAAATTCTATACTTTCTCCTAGTGTCTTCTTGACACGTCCTCCATATTTT
TTTAAAGAAAGTATTTGGAATATTTTGAGGCAATTTTTAATATTTAAGGAATTTTTCTTTG
GAATCATTTTTGGTTGACATCTCTGTTTTTTGTGGATCAGTTTTTTACTCTTCCACTCT
CTTTTCTATATTTTGCCCATCGGGGCTGCGGATACCTGGTTTTATTATTTTTTCTTTGCC
CAACGGGGCCGTGGATACCTGCCTTTTAATTCTTTTTTATTCGCCCATCGGGGCCGC
GGATACCTGCTTTTTATTTTTTTTTCCTTAGCCCATCGGGGTATCGGATACCTGCTGAT
TCCCTTCCCCTCTGAACCCCCAACACTCTGGCCCATCGGGGTGACGGATATCTGCTT
TTTAAAAATTTTCTTTTTTTGGCCCATCGGGGCTTCGGATACCTGCTTTTTTTTTTTTTA
TTTTTCCTTGCCCATCGGGGCCTCGGATACCTGCTTTAATTTTTGTTTTTCTGGCCCAT
CGGGGCCGCGGATACCTGCTTTGATTTTTTTTTTTCATCGCCCATCGGTGCTTTTTAT
GGATGAAAAAATGTTGGTTTTGTGGGTTGTTGCACTCTCTGGAATATCTACACTTTTTT
TTGCTGCTGATCATTTGGTGGTGTGTGAGTGTACCTACCGCTTTGGCAGAGAATGAC
TCTGCAGTTAAGCTAAGGGCGTGTTCAGATTGTGGAGGAAAAGTGGCCGCCATTTTA
GACTTGCCGCATAACTCGGCTTAGGGCTAGTCGTTTGTGCTAAGTTAAACTAGGGAG
GCAAGATGGATGATAGCAGGTCAGGCAGAGGAAGTCATGTGCATTGCATGAGCTAAA
CCTATCTGAATGAATTGATTTGGGGCTTGTTAGGAGCTTTGCGTGATTGTTGTATCGG
GAGGCAGTAAGAATCATCTTTTATCAGTACAAGGGACTAGTTAAAAATGGAAGGTTAG
GAAAGACTAAGGTGCAGGGCTTAAAATGGCGATTTTGACATTGCGGCATTGCTCAGC
ATGGCGGGCTGTGCTTTGTTAGGTTGTCCAAAATGGCGGATCCAGTTCTGTCGCAGT
GTTCAAGTGGCGGGAAGGCCACATCATGATGGGCGAGGCTTTGTTAAGTGGTTAGC

ATGGTGGTGGACATGTGCGGTCACACAGGAAAAGATGGCGGCTGAAGGTCTTGCCG
CAGTGTAAAACATGGCGGGCCTCTTTGTCTTTGCTGTGTGCTTTTCGTGTTGGGTTT
TGCCGCAGGGACAATATGGCAGGCGTTGTCATATGTATATCATGGCTTTTGTCACGTG
GACATCATGGCGGGCTTGCCGCATTGTTAAAGATGGCGGGTTTTGCCGCCTAGTGC
CACGCAGAGCGGGAGAAAAGGTGGGATGGACAGTGCTGGATTGCTGCATAACCCAA
CCAATTAGAAATGGGGGTGGAATTGATCACAGCCAATTAGAGCAGAAGATGGAATTAG
ACTGATGACACACTGTCCAGCTACTCAGCGAAGACCTGGGTGAATTAGCATGGCACT
TCGCAGCTGTCTTTAGCCAGTCAGGAGAAAGAAGTGGAGGGGCCACGTGTATGTCT
CCCAGTGGGCGGTACACCAGGTGTTTTCAAGGTCTTTTCAAGGACATTTAGCCTTTC
CACCTCTGTCCCCTCTTATTTGTCCCCTCCTGTCCAGTGCTGCCTCTTGCAGTGCTG
GATATCTGGCTGTGTGGTCTGAACCTCCCTCCATTCCTCTGTATTGGTGCCTCACCTA
AGGCTAAGTATACCTCCCCCCCCACCCCCCAACCCCCCCAACTCCCCACCCCCACC
CCCCACCCCCCACCTCCCCACCCCCCTACCCCCCTACCCCCCTACCCCCCTCTGGT
CTGCCCTGCACTGCACTGTTGCCATGGGCAGTGCTCCAGGCCTGCTTGGTGTGGAC
ATGGTGGTGAGCCGTGGCAAGGACCAGAATGGATCACAGATGATCGTTGGCCAACA
GGTGGCAGAAGAGGAATTCCTGCCTTCCTCAAGAGGAACACCTACCCCTTGGCTAAT
GCTGGGGTCGGATTTTGATTTATATTTATCTTTTGGATGTCAGTCATACAGTCTGATTTT
GTGGTTTGCTAGTGTTTGAATTTAAGTCTTAAGTGACTATTATAGAAATGTATTAAGAGG
CTTTATTTGTAGAATTCACTTTAATTACATTTAATGAGTTTTTGTTTTGAGTTCCTTAAAA
TTCCTTAAAGTTTTTAGCTTCTCATTACAAATTCCTTAACCTTTTTTTGGCAGTAGATAG
TCAAAGTCAAATCATTTCTAATGTTTTAAAAATGTGCTGGTCATTTTCTTTGAAATTGAC
TTAACTATTTTCCTTTGAAGAGTCTGTAGCACAGAAACAGTAAAAAATTTAACTTCATG
ACCTAATGTAAAAAAGAGTGTTTGAAGGTTTACACAGGTCCAGGCCTTGCTTTGTTCC
CATCCTTGATGCTGCACTAATTGACTAATCACCTACTTATCAGACAGGAAACTTGAATT
GCTGTGGTCTGGTGTCCTCTATTCAGACTTATTATATTGGAGTATTTCAATTTTTCGTT
GTATCCTGCCTGCCTAGCATCCAGTTCCTCCCCAGCCCTGCTCCCAGCAAACCCCTA
GTCTAGCCCCAGCCCTACTCCCACCCCGCCCCAGCCCTGCCCCAGCCCCAGTCCC
CTAACCCCCCAGCCCTAGCCCCAGTCCCAGTCCTAGTTCCTCAGTCCCGCCCAGCT
TCTCTCGAAAGTCACTCTAATTTTCATTGATTCAGTGCTCAAAATAAGTTGTCCATTGC
TTATCCTATTATACTGGGATATTCCGTTTACCCTTGGCATTGCTGATCTTCAGTACTGAC
TCCTTGACCATTTTCAGTTAATGCATACAATCCCATTTGTCTGTGATCTCAGGACAAAG
AATTTCCTTACTCGGTACGTTGAAGTTAGGGAATGTCAATTGAGAGCTTTCTATCAGA
GCATTATTGCCCACAATTTGAGTTACTTATCATTTTCTCGATCCCCTGCCCTTAAAGGA
GAAACCATTTCTCTGTCATTGCTTCTGTAGTCACAGTCCCAATTTTGAGTAGTGATCTT
TTCTTGTGTACTGTGTTGGCCACCTAAAACTCTTTGCATTGAGTAAAATTCTAATTGCC
AATAATCCTACCCATTGGATTAGACAGCACTCTGAACCCCATTTGCATTCAGCAGGGG
GTCGCAGACAACCCGTCTTTTGTTGGACAGTTAAAATGCTCAGTCCCAATTGTCATAG
CTTTGCCTATTAAACAAAGGCACCCTACTGCGCTTTTTGCTGTGCTTCTGGAGAATCC
TGCTGTTCTTGGACAATTAAAGAACAAAGTAGTAATTGCTAATTGTCTCACCCATTAAT
CATGAAGACTACCAGTCGCCCTTGCATTTGCCTTGAGGCAGCGCTGACTACCTGAGA
TTTAAGAGTTTCTTAAATTATTGAGTAAAATCCCAATTATCCATAGTTCTGTTAGTTACAC
TATGGCCTTTGCAAACATCTTTGCATAACAGCAGTGGGACTGACTCATTCTTAGAGCC

CCTTCCCTTGGAATATTAATGGATACAATAGTAATTATTCATGGTTCTGCGTAACAGAG
AAGACCCACTTATGTGTATGCCTTTATCATTGCTCCTAGATAGTGTGAACTACCTACCA
CCTTGCATTAATATGTAAAACACTAATTGCCCATAGTCCCACTCATTAGTCTAGGATGT
CCTCTTTGCCATTGCTGCTGAGTTCTGACTACCCAAGTTTCCTTCTCTTAAACAGTTG
ATATGCATAATTGCATATATTCATGGTTCTGTGCAATAAAAATGGATTCTCACCCCATCC
CACCTTCTGTGGGATGTTGCTAACGAGTGCAGATTATTCAATAACAGCTCTTGAACAG
TTAATTTGCACAGTTGCAATTGTCCAGAGTCCTGTCCATTAGAAAGGGACTCTGTATC
CTATTTGCACGCTACAATGTGGGCTGATCACCCAAGGACTCTTCTTGTGCATTGATGT
TCATAATTGTATTTGTCCACGATCTTGTGCACTAACCCTTCCACTCCCTTTGTATTCCA
GCAGGGGACCCTTACTACTCAAGACCTCTGTACTAGGACAGTTTATGTGCACAATCCT
AATTGATTAGAACTGAGTCTTTTATATCAAGGTCCCTGCATCATCTTTGCTTTACATCAA
GAGGGTGCTGGTTACCTAATGCCCCTCCTCCAGAAATTATTGATGTGCAAAATGCAAT
TTCCCTATCTGCTGTTAGTCTGGGGTCTCATCCCCTCATATTCCTTTTGTCTTACAGCA
GGGGGTACTTGGGACTGTTAATGCGCATAATTGCAATTATGGTCTTTTCCATTAAATTA
AGATCCCAACTGCTCACACCCTCTTAGCATTACAGTAGAGGGTGCTAATCACAAGGA
CATTTCTTTTGTACTGTTAATGTGCTACTTGCATTTGTCCCTCTTCCTGTGCACTAAAG
ACCCCACTCACTTCCCTAGTGTTCAGCAGTGGATGACCTCTAGTCAAGACCTTTGCA
CTAGGATAGTTAATGTGAACCATGGCAACTGATCACAACAATGTCTTTCAGATCAGAT
CCATTTTATCCTCCTTGTTTTACAGCAAGGGATATTAATTACCTATGTTACCTTTCCCTG
GGACTATGAATGTGCAAAATTCCAATGTTCATGGTCTCTCCCTTTAAACCTATATTCTA
CCCCTTTTACATTATAGAAAGGGATGCTGGAAACCCAGAGTCCTTCTCTTGGGACTCT
TAATGTGTATTTCTAATTATCCATGACTCTTAATGTGCATATTTTCAATTGCCTAATTGAT
TTCAATTGTCTAAGACATTTCAAATGTCTAATTGATTAGAACTGAGTCTTTTATATCAAG
CTAATATCTAGCTTTTATATCAAGCTAATATCTTGACTTCTCAGCATCATAGAAGGGGGT
ACTGATTTCCTAAAGTCTTTCTTGAATTTCTATTATGCAAAATTGCCCTGAGGCCGGGT
GTGGTGGCTCACACCTGTAATCCCAGCACTTTGGGAGGCTGAGGTGGGAAGATCCC
TTACTGCCAGGAGTTTGAGACCAGCCTGGCCAACATTAAAAAAAAAAAAAAAGTAAGA
CAATTGCCCTGGAATCCCATCCCCCTCACACCTCCTTGGCAAAGCAGCAGGAGTGC
TAACTAGCTAGTGCTTCTTCTCTTATACTGCTTAAATGCGCATAATTAGCAGTAGTTGAT
GTGCCCCTATGTTAGAGTAGAATCCCGCTTCCTTGCTCCATTTGCATTACTGCAGGAG
CTTCTAACTAGCCTGAATTCACTCTCTTGGACTGTTAATGTGCATACTTATATTTGCTG
CTGTACTTTTTTACCATGTAAGGACCCCACCCACTGTATTTACATCCCAGCTGGAAGT
ACCTACTACTTAAGACCCTTAGACTAGTAAAGTTAGCGTGCATAATCTTAGGTGTTATAT
ACACATTTTCAGTTGCATACAGTTGTGCCTTTTATCAGGACTCCTGTACTTATCAAAGC
AGAGAGTGCTAATCAATATTAAGCCCTTCTCTTCGAACTGTAGATGGCATGTAATTGCA
GTTGTCAATGGTCCTTCAATTAGACTTGGGTTTCTGACCTATCACACCCTCTTTGCTT
TATTGCATGGGGTACTATTCACTTAAGGCCCCTTTCTCAAACTGTTAATGTGCCTAATG
ACAATTACATCAGTATCCTTCCTTTTGAAGGACAGCATGGTTGGTGACACCTAAGGCC
CCATTTCTTGGCCTCCCAATATGTGTGATTGTATTTGTCGAGGTTGCTATGCACTAGA
GAAGGAAAGTGCTCCCCTCATCCCCACTTTTCCCTTCCAGCAGGAAGTGCCCACCC
CATAAGACCCTTTTATTTGGAGAGTCTAGGTGCACAATTGTAAGTGACCACAAGCATG
CATCTTGGACATTTATGTGCGTAATCGCACACTGCTCATTCCATGTGAATAAGGTCCTA

CTCTCCGACCCCTTTTGCAATACAGAAGGGTTGCTGATAACGCAGTCCCCTTTTCTT
GGCATGTTGTGTGTGATTATAATCGTCTGGGATCCTATGCACTAGAAAAGGAGGGTCC
TCTCCACATACCTCAGTCTCACCTTTCCCTTCCAGCAGGGAGTGCCCACTCCATAAG
ACTCTCACATTTGGACAGTCAAGGTGCGTAATTGTTAAGTGAACACAACCATGCACCT
TAGACATGGATTTGCATAACTACACACAGCTCAACCTATCTGAATAAAATCCTACTCTC
AGACCCCTTTTGCAGTACAGCAGGGGTGCTGATCACCAAGGCCCTTTTTCCTGGCC
TGGTATGCGTGTGATTATGTTTGTCCCGGTTCCTGTGTATTAGACATGGAAGCCTCCC
CTGCCACACTCCACCCCCAATCTTCCTTTCCCTTCCGGCAGGGAGTGCCCTCTCCAT
AAGACGCTTACGTTTGGACAATCAAGGTGCACAGTTGTAAGTGACCACAGGCATACA
CCTTGGACATTAATGTGCATAACCACTTTGCCCATTCCATCTGAATAAGGTCCTACTCT
CAGACCCCTTTTGCAGTACAGCAGGGGTGCTGATCACCAAGGCCCCTTTTCTTGGC
CTGTTATGTGCGTGATTATATTTGTCTGGGTTCCTGTGTATTAGACAAGGAAGCCTTCC
CCCCGCCCCCACCCCCACTCCCAGTCTTCCTTTCCCTTCCAGCAGGGAGTGCCCCC
TCCATAAGATCATTACATTTGGACAATCAAGGTGCACAATTATAAGTGACCACAGCCAT
GCACCTTGGACATTATTGGACATTAATGTGCGTAACTGCACATGGCCCATCCCATCTG
AATAAGGTCCTACTCTCAGATGCCCTTTGCAGTACAGCAGGGGTACTGAATCACCAA
GGCCCTTTTTCTTGGCCTGTTATGTGTGTGATTATATTTATCCCAGTTTCTGTGTAATAG
ACATGAAAGCCTCCCCTGCCACACCCCACCTCCAATCTTCCTTTCCCTTCCACCAGG
GAGTGTCCACTCCATATACCCTTACATTTGGACAATCAAGGTGCACAATTGTAAGTGA
GCATAGGCACTCACCTTGGACATGAATGTGCATAACTGCACATGGCCCATCCCATCT
GAATAAGGTCCTACTCTCAGACCCTTTTTGCAGTACAGCAGGGGTGCTGATCACCAA
GGCCCCTTTTCCTGGCCTGTTATGTGTGTGATTATATTTGTTCCAGTTCCTGTGTAATA
GACATGGAAGCCTCCCCTGCCACACTCCACCCCCAATCTTCCTTTCCCTTCTGGCAG
GAAGTACCCGCTCCATAAGACCCTTACATTTGGACAGTCAAGGTGCACAATTGTATGT
GACCACAACCATGCACCTTGGACATAAATGTGTGTAACTGCACATGGCCCATCCCAT
CTGAATAAGGTCCTACTCTCAGACCCCTTTTGCAGTACAGTAGGTGTGCTGATAACCA
AGGCCCCTCTTCCTGGCCTGTTAACGTATGTGATTATATTTGTCTGGGTTCCAGTGTA
TAAGACATGGAAGCCTCCCCTGCCCCACCCCACCCTCAATCTTCCTTTCCCTTCTGG
CAGGGAGTGCCAGCTCCATAAGAACCTTACATTTGGACAGTCAAGGTGCACAATTCT
AAGTGACCGCAGCCATGCACCTTGGTCAATAATGTGTGTAACTGCACACGGCCTATC
TCATCTGAATAAGGCCTTACTCTCAGACCCCTTTTGCAGTACAGCAGGGGTGCTGATA
ACCAAGGCCCATTTTCCTGGCCTGTTATGTGTGTGATTATATTTGTCCAGGTTTCTGT
GTACTAGACAAGGAAGCCTCCTCTGCCCCATCCCATCTACGCATAATCTTTCTTTTCC
TCCCAGCAGGGAGTGCTCACTCCATAAGACCCTTACATTTGGACAATCAAGGTGCAC
AATTGTAAGTGACCACAACCATGCATCTTGGAAATTTATGTGCATAACTGCACATGGCT
TATCCTATTTGAATAAAGTCCTACTCTCAGACCCCCTTTGCAGTATAGCTGGGGTGCT
GATCACTGAGGCCTCTTTGCTTGGCTTGTCTATATTCTTGTGTACTAGATAAGGGCAC
CTTCTCATGGACTCCCTTTGCTTTTCAACAAGGAGTACCCACTACTTTTTAAGATTCTT
ATATTTGTCCAAAGTACATGGTTTTAATTGACCACAACAATGTCCCTTGGACATTAATG
TATGTAATCACCACATGGTTCATCCTAATTAAACAAAGTTCTACCTTCTCACCCTCCATT
TGCAGTATACCAGGGTTGCTGACCCCCTAAGTCCCCTTTTCTTGGCTTGTTGACATG
CATAATTGCATTTATGTTGGTTCTTGTGCCCTAGACAAGGATGCCCCACCTCTTTTCAA

TAGTGGGTGCCCACTCCTTATGATCTTTACATTTGAACAGTTAATGTGAATAATTGCAG
TTGTCCACAACCCTATCACTTCTAGGACCATTATACCTCTTTTGCATTACTGTGGGGTA
TACTGTTTCCCTCCAAGGCCCCTTCTGGTGGACTATCAACATATAATTGAAATTTTCTT
TTGTCTTTGTCAGTAGATTAAGGTCATACCCCATCACCTTTCCTTTGTAGTACAACAGG
GTGTCCTGATCAACCAAAGTCCTGTTGTTTTGGACTGTTAATATGTGCAATTACATTTG
CTCCTGATCTGTGCACTAGATAAGGATCCTACCTACTTTCTTAGTGTTTTTAGCAGGTA
GTGCCCACTACTCAAGACTGTCACTTGGAATGTTCATGTGCACAAACTCAATTCTCTA
AGCATGTTCCTGTACCACCTTTGCTTTAGAGCAGGGGGATGATATTCACTAAGTGCCC
CTTCTTTTGGACTTAATATGCATTAATGCAATTGTCCACCTCTTCTTTTAGACTAAGAGT
TGATCTCCACATATTCCCCTTGCATCAGGGGCATGTTAATTATGAATGAACCCTTTTCT
TTTAATATTAATGTCATAATTGTATTTGTGGACCTGTGTAGGAGAAAAAGACCCTATGTT
CCTCCCATTACCCTTTGGATTGCTGCTGAGAAGTGTTAACTACTCATAATCTCAGCTC
TTGGACAATTAATAGCATTAATAACAATTATCAAGGGCACTGATCATTAGATAAGACTCC
TGCTTCCTCGTTGCTTACATCGGGGGTACTGACCCACTAAGGCCCCTTGTACTGTTA
ATGTGAATATTTGCAATTATATATGTCTCCTTCTGGTAGAGTGGGATATTATGCCCTAGT
ATCCCCTTTGCATTACTGCAGGGGCTGCTGACTACTCAAAACTTCTCCTGGGACTGT
TAATAGGCACAATGGCAGTTATCAATGGTTTTCTCCCTCCCTGACCTTGTTAAGCAAG
CGCCCCACCCCACCCTTAGTTTCCCATGGCATAATAAAGTATAAGCATTGGAGTATTC
CATGCACTTGTCTATCAAACAGTGGTCCATACTCCCAACCCTTTTGCATTGCGCCAGT
GTGTAAAATCACAGGTAGCCATGGTGTCATGCTTTATATACGAAGTCTTCCCTCTCTCT
GCCCCTTGTGTGCCCTTGGCCCCTTTTTACAGACTATTGCTCACAATCTCAGGTGTC
CATATTTGCAGCTATTAGGTAAGATTGTGCTGTCTCCCTCTTCCCTTCCCTCTGCCCT
GCCCCTTTTGCCTCTTTGCTGGGTAATGTTGACCAGACAAGGCCCTTTCTCTTGGAC
TTAAACAATTCTCAGTTGCACTTTCCTTGGTCCCACCCATTATACATGAACCCCTCTAC
TTCCTTTCGCATTGCTTCTGAGTATGCTGACTACCCAAAGCCCCTTCTGTGTTATTAAT
AAACACAGTACTGATTGTCCCATTTTTCAGCCCATCAGTCCAAGATCTCCCTACCACT
TTGGTGTGTTGGTGCAGTGTTGACTATGAAAAGCAGGCCTGAACTAGGTGGATAAGC
CTTCACTCATTTTCTTTCATTTATTAATGATCCTAGTTTCAATTATTGTCAGATTCTGGG
GACAAGAACCATTCTTGCCCACCTGTGTTACTGCTTTACTGTGCAAAATACTGAAGGC
AAGTCAGACCCAGGGAGCTGGATTGCCATCCTTTATTTTGTGTTTCCAGTGTACACTA
TAAAATTGTCTCCCCAGGAAGGAAGGTTGGCACTTTCTCTGCATTCTTCTTTCCAGAG
CAGATTGCCTGGTTAAGAATCTCTTGTTGTCCCCTTTGTATATTGTTATTGTAAAGTGC
CAAATGCCAGGATACAGCCAGAAAAATTGCTTATTATTATTAAAAAAATTTTTTTAAGAA
AGACATCTGGATTGTAGGGTGGACTCGATAACCTGGTCATTATTTTTTTGAAGCCAAA
ATATCCATTTATACTATGTACCTGGTGACCAGTGTCTCTCATTTTAACTGAGGGTGGTG
GGTCTGTGGATAGAACACTGACTCTTGCTATTTTAATATCAAAGATATTCTAGAGTGGA
ACTCTTAAGACCAGTATCTTTGTGTGGGCTTTACCAGCATTCACTTTTAGAAAAACTAC
CTAAATTTTATAATCCTTTAATTTCTTCATCTGGAGCACCTGCCCCTACTTATTTCAAGA
AGATTGCAGTAAAACGATTAAATGAGGGAACATATGCAGAGGTGCTTTTAAAAAGCAT
ATGCCACCTTTTTTATTAATTATTATATAAAATGAAGCATTTAATTATAGTAATAATTTGAA
GTAGTTTGAAGTACCACACTGAGGTGAGGACTTAAAAATGATAAGACGAGTTCCCTAT
TTTATAAGAAAAATAAGCCAAAATTAAATATTCTTTTGGATATAAATTTCAACAGTGAGAT

AGCTGCCTAGTGGAAATGAATAATATCCCAGCCACTAGTGTACAGGGTGTTTTGTGGC
ACAGGATTATGTAATATGGAACTGCTCAAGCAAATAACTAGTCATCACAACAGCAGTTC
TTTGTAATAACTGAAAAAGAATATTGTTTCTCGGAGAAGGATGTCAAAAGATCGGCCC
AGCTCAGGGAGCAGTTTGCCCTACTAGCTCCTCGGACAGCTGTAAAGAAGAGTCTC
TGGCTCTTTAGAATACTGATCCCATTGAAGATACCACGCTGCATGTGTCCTTAGTAGT
CATGTCTCCTTAGGCTCCTCTTGGACATTCTGAGCATGTGAGACCTGAGGACTGCAA
ACAGCTATAAGAGGCTCCAAATTAATCATATCTTTCCCTTTGAGAATCTGGCCAAGCTC
CAGCTAATCTACTTGGATGGGTTGCCAGCTATCTGGAGAAAAAGATCTTCCTCAGAAG
AATAGGCTTGTTGTTTTACAGTGTTAGTGATCCATTCCCTTTGACGATCCCTAGGTGG
AGATGGGGCATGAGGATCCTCCAGGGGAAAAGCTCACTACCACTGGGCAACAACCC
TAGGTCAGGAGGTTCTGTCAAGATACTTTCCTGGTCCCAGATAGGAAGATAAAGTCTC
AAAAACAACCACCACACGTCAAGCTCTTCATTGTTCCTATCTGCCAAATCATTATACTT
CCTACAAGCAGTGCAGAGAGCTGAGTCTTCAGCAGGTCCAAGAAATTTGAACACACT
GAAGGAAGTCAGCCTTCCCACCTGAAGATCAACATGCCTGGCACTCTAGCACTTGA
GGATAGCTGAATGAATGTGTATTTCTTTGTCTCTTTCTTTCTTGTCTTTGCTCTTTGTTC
TCTATCTAAAGTGTGTCTTACCCATTTCCATGTTTCTCTTGCTAATTTCTTTCGTGTGTG
CCTTTGCCTCATTTTCTCTTTTTGTTCACAAGAGTGGTCTGTGTCTTGTCTTAGACATA
TCTCTCATTTTTCATTTTGTTGCTATTTCTCTTTGCTCTCCTAGATGTGGCTCTTCTTTC
ACGCTTTATTTCATGTCTCCTTTTTGGGTCACATGCTGTGTGCTTTTTGTCCTTTTCTT
GTTCTGTCTACCTCTCCTTTCTCTGCCTACCTCTCTTTTCTCTTTGTGAACTGTGATTA
TTTGTTACCCCTTCCCCTTCTCGTTCGTTTTAAATTTCACCTTTTTTCTGAGTCTGGCC
TCCTTTCTGCTGTTTCTACTTTTTATCTCACATTTCTCATTTCTGCATTTCCTTTCTGCC
TCTCTTGGGCTATTCTCTCTCTCCTCCCCTGCGTGCCTCAGCATCTCTTGCTGTTTGT
GATTTTCTATTTCAGTATTAATCTCTGTTGGCTTGTATTTGTTCTCTGCTTCTTCCCTTT
CTACTCACCTTTGAGTATTTCAGCCTCTTCATGAATCTATCTCCCTCTCTTTGATTTCAT
GTAATCTCTCCTTAAATATTTCTTTGCATATGTGGGCAAGTGTACGTGTGTGTGTGTCA
TGTGTGGCAGAGGGGCTTCCTAACCCCTGCCTGATAGGTGCAGAACGTCGGCTATC
AGAGCAAGCATTGTGGAGCGGTTCCTTATGCCAGGCTGCCATGTGAGATGATCCAAG
ACCAAAACAAGGCCCTAGACTGCAGTAAAACCCAGAACTCAAGTAGGGCAGAAGGT
GGAAGGCTCATATGGATAGAAGGCCCAAAGTATAAGACAGATGGTTTGAGACTTGAG
ACCCGAGGACTAAGATGGAAAGCCCATGTTCCAAGATAGATAGAAGCCTCAGGCCTG
AAACCAACAAAAGCCTCAAGAGCCAAGAAAACAGAGGGTGGCCTGAATTGGACCGA
AGGCCTGAGTTGGATGGAAGTCTCAAGGCTTGAGTTAGAAGTCTTAAGACCTGGGA
CAGGACACATGGAAGGCCTAAGAACTGAGACTTGTGACACAAGGCCAACGACCTAA
GATTAGCCCAGGGTTGTAGCTGGAAGACCTACAACCCAAGGATGGAAGGCCCCTGT
CACAAAGCCTACCTAGATGGATAGAGGACCCAAGCGAAAAAGGTATCTCAAGACTAA
CGGCCGGAATCTGGAGGCCCATGACCCAGAACCCAGGAAGGATAGAAGCTTGAAGA
CCTGGGGAAATCCCAAGATGAGAACCCTAAACCCTACCTCTTTTCTATTGTTTACACT
TCTTACTCTTAGATATTTCCAGTTCTCCTGTTTATCTTTAAGCCTGATTCTTTTGAGATG
TACTTTTTGATGTTGCCGGTTACCTTTAGATTGACAGTATTATGCCTGGGCCAGTCTTG
AGCCAGCTTTAAATCACAGCTTTTACCTATTTGTTAGGCTATAGTGTTTTGTAAACTTCT
GTTTCTATTCACATCTTCTCCACTTGAGAGAGACACCAAAATCCAGTCAGTATCTAATC

TGGCTTTTGTTAACTTCCCTCAGGAGCAGACATTCATATAGGTGATACTGTATTTCAGT
CCTTTCTTTTGACCCCAGAAGCCCTAGACTGAGAAGATAAAATGGTCAGGTTGTTGG
GGAAAAAAAAGTGCCAGGCTCTCTAGAGAAAAATGTGAAGAGATGCTCCAGGCCAAT
GAGAAGAATTAGACAAGAAATACACAGATGTGCCAGACTTCTGAGAAGCACCTGCCA
GCAACAGCTTCCTTCTTTGAGCTTAGGTGAGCAGGATTCTGGGGTTTGGGATTTCTA
GTGATGGTTATGGAAAGGGTGACTGTGCCTGGGACAAAGCGAGGTCCCAAGGGGA
CAGCCTGAACTCCCTGCTCATAGTAGTGGCCAAATAATTTGGTGGACTGTGCCAACG
CTACTCCTGGGTTTAATACCCATCTCTAGGCTTAAAGATGAGAGAACCTGGGACTGTT
GAGCATGTTTAATACTTTCCTTGATTTTTTTCTTCCTGTTTATGTGGGAAGTTGATTTAA
ATGACTGATAATGTGTATGAAAGCACTGTAAAACATAAGAGAAAAACCAATTAGTGTAT
TGGCAATCATGCAGTTAACATTTGAAAGTGCAGTGTAAATTGTGAAGCATTATGTAAAT
CAGGGGTCCACAGTTTTTCTGTAAGGGGTCAAATCATAAATACTTTAGACTGTGGGCC
ATATGGTTTCTGTTACATATTTGTTTTTTAAACAACGTTTTTATAAGGTCAAAATCATTCT
TAGTTTTTGAGCCAATTGGATTTGGCCTGCTGTTCATAGCTTACCACCCCCTGATGTAT
TATTTGTTATTCAGAGAAAATTTCTGAATACTACTAGTTTCCTTTTCTGTGCCTGTCCCT
GTGCTAGGCACTAAAAATGCAATGATTATTGATATCTAGGTGACCTGAAAAAAAATAGT
GAATGTGCTTTGTAAACTGTAAAGCACTTGTATTCTACTGTGATAAGCGTTGTGGATAC
AAAGAAAGGAGCAAGCATAAAAAAGTGCTCTTTCAAAAGGATATAGTACTATGCAGAC
ACAAGGAATTGTTTGATAAATGAATAAATTATATGTATATTTGAGGCCAATTTGTGTTTG
CTGCTCTGGTAATTTTGAGTAAAAATGCAGTATTCCAGGTATCAGAAACGAAAACACA
TGGAAACTGCTTTTAAACTTTAAAATATACTGAAAACATAAGGGACTAAGCTTGTTGTG
GTCACCTATAATGTGCCAGATACCATGCTGGGTGCTAGAGCTACCAAAGGGGGAAAA
GTATTCTCATAGAACAAAAAATTTCAGAAAGGTGCATATTAAAGTGCTTTGTAAACTAA
AGCATGATACAAATGTCAATGGGCTACATATTTATGAATGAATGAATGGATGAATGAATA
TTAAGTGCCTCTTACATACCAGCTATTTTGGGTACTGTAAAATACAAGATTAATTCTCCT
ATGTAATAAGAGGAAAGTTTATCCTCTATACTATTCAGATGTAAGGAATGATATATTGCT
TAATTTTAAACAATCAAGACTTTACTGGTGAGGTTAAGTTAAATTATTACTGATACATTTT
TCCAGGTAACCAGGAAAGAGCTAGTATGAGGAAATGAAGTAATAGATGTGAGATCCA
GACCGAAAGTCACTTAATTCAGCTTGCGAATGTGCTTTCTAAATTATAAAGCACTTGTA
AATGAAAAATTTGATGCTTTCTGTATGAATAAAACTTTCTGTAAGCTAGGTATTGTCTCT
ACAAAATTCTCATTGTATAGTTAAACCACAGTGAGAAGGGTTCTATAAGTAGTTATACA
AACCAAGGGTTTAAATACCTGTTAAATAGATCAATTTTGATTGCCTACTATGTGAACTC
ACTGTTAAAGGCACTGAAAATTTATCATATTTCATTTAGCCACAGCCAAAAATAAGGCA
ATACCTATGTTAGCATTTTGTGAACTCTAAGGCACCATATAAATGTAACTGTTGATTTTC
TCACTTGGTGCTGGGTACTAGGTTTATAAAATTGTATGATAGTTATTATATTGTGCAAAT
AAAGTAGGAAAATTTGAATAACAATGATTATCTTTTGAATACGCATACGCAAGGGATTG
GTTGTCTGAAGAATGCCACTATAGTAGTTATCTATTGTGTGCCAATCTCATTGCTAGGC
ATTGGGGATGCAAAGATAAACCATCTTTATTGTGTCTTGGGTAGCAGAAGAAAATATGT
GTAAAATCAATTTATAATTTGTAAACTGCCACCCATATATAAGCTATATCTGCTGAATGAT
CATTGATTACTCTTATCCTTAGAGATAACAACTGGGGGCACAAACATTTATTATCATTAT
TGAACCTACAACAGAGATCTATGTGTAGATTTACAAAGCCTACAGTTCTATACAGATAG
GAATGAACTATTGGCTTACTGAATGGTGATTACTTTCTGTGGGGCTCGGAACTACATG

CCCTAGGATATAAAAATGATGTTATCATTATAGAGTGCTCACAGAAGGAAATGAAGTAA
TATAGGTGTGAGATCCAGACCAAAAGTCATTTAACAAGTTTATTCAGTGATGAAAACAT
GGGACAAATGGACTAATATAAGGCAGTGTACTAAGCTGAGTAGAGAGATAAAGTCCTG
TCCAGAAGATACATGCTTCCTGGCCTGATTGAGGAGATGGAAAATTTTTGCAAAAAAC
AAGGTGTTGTGGTCTTCCATCCAGTTTCTTAAGTGCTGATGATAAAAGTGAATTAGAC
CCACCTTGACCTGGCCTACAGAAGTAAAGGAGTAAAAATAAATGCCTCAGGCGTGCT
TTTTGATTCATTTGATAAACAAAGCATCTTTTATGTGGAATATACCATTCTGGGTCCTGA
GGATAAGAGAGATGAGGGCATTAGATCACTGACAGCTGAAGATAGAAGAACATCTTT
GGTTTGATTGTTTAAATAATATTTCAATGCCTATTCTCTGCAAGGTACTATGTTTCGTAA
ATTAAATAGGTCTGGCCCAGAAGACCCACTCAATTGCCTTTGAGATTAAAAAAAAAAA
AAAAAAGAAAGAAAAATGCAAGTTTCTTTCAAAATAAAGAGACATTTTTCCTAGTTTCA
GGAATCCCCCAAATCACTTCCTCATTGGCTTAGTTTAAAGCCAGGAGACTGATAAAAG
GGCTCAGGGTTTGTTCTTTAATTCATTAACTAAACATTCTGCTTTTATTACAGTTAAATG
GTTCAAGATGTAACAACTAGTTTTAAAGGTATTTGCTCATTGGTCTGGCTTAGAGACA
GGAAGACATATGAGCAATAAAAAAAAGATTCTTTTGCATTTACCAATTTAGTAAAAATTT
ATTAAAACTGAATAAAGTGCTGTTCTTAAGTGCTTGAAAGACGTAAACCAAAGTGCAC
TTTATCTCATTTATCTTATGGTGGAAACACAGGAACAAATTCTCTAAGAGACTGTGTTT
CTTTAGTTGAGAAGAAACTTCATTGAGTAGCTGTGATATGTTCGATACTAAGGAAAAAC
TAAACAGATCACCTTTGACATGCGTTGTAGAGTGGGAATAAGAGAGGGCTTTTTATTT
TTTCGTTCATACGAGTATTGATGAAGATGATACTAAATGCTAAATGAAATATATCTGCTC
CAAAAGGCATTTATTCTGACTTGGAGATGCAACAAAAACACAAAAATGGAATGAAGTG
ATACTCTTCATCAAACAGAAGTGACTGTTATCTCAACCATTTTGTTAAATCCTAAACAG
AAAACAAAAAAAATCATGACGAAAAGACACTTGCTTATTAATTGGCTTGGAAAGTAGA
ATATAGGAGAAAGGTTACTGTTTATTTTTTTTCATGTATTCATTCATTCTACAAATATATTC
GGGTGCCAATAGGTACTTGGTATAAGGTTTTTGGCCCCAGAGACATGGGAAAAAAT
GCATGCCTTCCCAGAGAATGCCTAATACTTTCCTTTTGGCTTGTTTTCTTGTTAGGGG
CATGGCTTAGTCCCTAAATAACATTGTGTGGTTTAATTCCTACTCCGTATCTCTTCTAC
CACTCTGGCCACTACGATAAGCAGGTAGCTGGGTTTTGTAGTGAGCTTGCTCCTTAA
GTTACAGGAACTCTCCTTATAATAGACACTTCATTTTCCTAGTCCATCCCTCATGAAAA
ATGACTGACCACTGCTGGGCAGCAGGAGGGATGATGACCAACTAATTCCCAAACCC
CAGTCTCATTGGTACCAGCCTTGGGGAACCACCTACACTTGAGCCACAATTGGTTTT
GAAGTGCATTTACAAGGTTTGTCTATTTTCAGTTCTTTACTTTTTACATGCTGACACATA
CATACACTGCCTAAATAGATCTCTTTCAGAAACAATCCTCAGATAACGCATAGCAAAAT
GGAGATGGAGACATGATTTCTCATGCAACAGCTTCTCTAATTATACCTTAGAAATGTTC
TCCTTTTTATCATCAAATCTGCTCAAGAAGGGCTTTTTATAGTAGAATAATATCAGTGGA
TGAAAACAGCTTAACATTTTACCATGCTTAAGTTTTAAGAATAAAATAAAAATTGGAAAT
AATTGGCCAAAATTGAAAGGAAAAATTTTTTTAAAATTTCTCTAAATGTAGGCCTGGCT
GGGCTTTGACCTTTTCCGTTTTTAAATCACTCACAGAGGGTGGGACAGGAGGAAGA
GTGAAGGAAAAGGTCAAACCTGTTTTAAGGGCAACCTGCCTTTGTTCTGAATTGGTC
TTAAGAACATTACCAGCTCCAGGTTTAAATTGTTCAGTTTCATGCAGTTCCAATAGCTG
ATCATTGTTGAGATGAGGACAAAATCCTTTGTCCTCACTAGTTTGCTTTACATTTTTGA
AAAGTATTATTTTTGTCCAAGTGCTTATCAACTAAACCTTGTGTTAGGTAAGAATGGAA

TTTATTAAGTGAATCAGTGTGACCCTTCTTGTCATAAGATTATCTTAAAGCTGAAGCCA
AAATATGCTTCAAAAGAAGAGGACTTTATTGTTCATTGTAGTTCATACATTCAAAGCAT
CTGAACTGTAGTTTCTATAGCAAGCCAATTACATCCATAAGTGGAGAAGGAAATAGATA
AATGTCAAAGTATGATTGGTGGAGGGAGCAAGGTTGAAGATAATCTGGGGTTGAAAT
TTTCTAGTTTTCATTCTGTACATTTTTAGTTAGACATCAGATTTGAAATATTAATGTTTAC
CTTTCAATGTGTGGTATCAGCTGGACTCAGTAACACCCCTTTCTTCAGCTGGGGATG
GGGAATGGATTATTGGAAAATGGAAAGAAGAAAGTAACTAAAAGCCTTCCTTTCACAG
TTTCTGGCATCACTACCACTACTGATTAAACAAGAATAAGAGAACATTTTATCATCATCT
GCTTTATTCACATAAATGAAGTTGTGATGAATAAATCTGCTTTTATGCAGACACAAGGA
ATTAAGTGGCTTCGTCATTGTCCTTCTACCTCAAAGATAATTTATTCCAAAAGCTAAGA
TAAATGGAAGACTCTTGAACTTGTGAACTGATGTGAAATGCAGAATCTCTTTTGAGTC
TTTGCTGTTTGGAAGATTGAAAAATATTGTTCAGCATGGGTGACCACCAGAAAGTAAT
CTTAAGCCATCTAGATGTCACAATTGAAACAAACTGGGGAGTTGGTTGCTATTGTAAA
ATAAAATATACTGTTTTGAAAACTTTGAAAAAAAAAAAAAAAA

**Table S6. Sequence of the mouse *Xist* locus**

**exon 1**

**exon 2**

**exon 3**

**exon 4**

**exon 5**

**exon 6**

**exon 7**

repeat A

repeat F

repeat B

repeat C

repeat D

repeat E

CGGCTTGCTCCAGCCATGTTTGCTCGTTTCCCGTGGATGTGCGGTTCTTCCGTGGTT
TCTCTCCATCTAAGGAGCTTTGGGGGAACATTTTTAGTTCCCCTACCACCAAGCCTTA
TGGCTTATTTAAGAAAACATATCAAAATTCCACGAGATTTTTGACGTTTTGATATGTTCT
GGTAAGATTTTTTTTTTGACATGTCCTCCATACTTTTTGATATTTGTAATATTTTCAGTCA
ATTTTTCATTTTTAAGGAATATTTCTTTGTTGTGCCTTTTGGTTGATACTTGTGTGTGTA
TGGTGGACTTACCTTTCTTTCATTGTTTATATATTCTTGCCCATCGGGGCCACGGATAC
CTGTGTGTCCTCCCCGCCATTCCATGCCCAACGGGGTTTTGGATACTTACCTGCCTT
TTCATTCTTTTTTTTTCTTATTATTTTTTTTTCTAAACTTGCCCATCTGGGCTGTGGATAC
CTGCTTTTATTCTTTTTTTCTTCTCCTTAGCCCATCGGGGCCATGGATACCTGCTTTTT
GTAAAAAAAAAAAAAAAAACAAAAAAACCTTTCTCGGTCCATCGGGACCTCGGATACC
TGCGTTTAGTCTTTTTTTCCCATGCCCAACGGGGCCTCGGATACCTGCTGTTATTATT
TTTTTTTCTTTTTCTTTTGCCCATCGGGGCTGTGGATACCTGCTTTAAATTTTTTTTTTC
ACGGCCCAACGGGGCGCTTGGTGGATGGAAATATGGTTTTGTGAGTTATTGCACTAC
CTGGAATATCTATGCCTCTTATTTGCGTGTACTGTTGCTGCTGATCGTTTGGTGCTGT
GTGAGTGAACCTATGGCTTAGAAAAACGACTTTGCTCTTAAACTGAGTGGGTGTTCA
GGGCGTGGAGAGCCCGCGTCCGCCATTATGGCTTCTGCGTGATACGGCTATTCTCG
AGCCAGTTACGCCAAGAATTAGGACACCGAGGAGCACAGCGGACTGGATAAAAGCA
ACCAATTGCGCTGCGCTAGCTAAAGGCTTTCTTTATATGTGCGGGGTTGCGGGATTC
GCCTTGATTTGTGGTAGCATTTGCGGGGTTGTGCTAGCCGGAAGTAGAAAGCCAAG
GAGTGCTCGTATTAGTGTGCGGTGTTGCGCGGAAGCCGCAGAGGACTAGGGGATAG
GGCTCAGCGTGGGTGTGGGGATTGGGCAGGGTGTGTGTGCATATGGACCCCTGGC
GCGGTCCCCCGTGGCTTTAAGGGCTGCTCAGAAGTCTATAAAATGGCGGCTCGGGG
GCTCCACCCGAGGCTCGACAGCCCAATCTTTGTTCTGGTGTGTAGCAATGGATTATA

GGACATTTAGGTCGTACAGGAAAAGATGGCGGCTCAAGTTCTTGGTGCGGTATAACG
CAAAGGGCTTTGTGTGTCACATGTCAGCTTCATGTCTGAGTTAGCCTGGAGAGGTGG
CACATGCTCTTGAATGTGTCTAAGATGGCGGAAGTCATGTGACCTGCCCTCTAGTGG
TTTCTTTCAGTGATTTTTTTTTTGGCGGGCTTTAGCTACTTGGCGGGCTTTGCCCGAG
GGTACACTTGGTGCATTATGGTAGGGTGTGGTTGGTCCTACCTTGTGCCACTCGAAG
CTGAGGCAAGGCTAAGTGGAAGTGTTGGTTGCCACTTGACGTAACTCGTCAGAAAT
GGGCACAAGTGTGAAAGTGTTGGTGTTTGCTTGACTTCCAGTTAGAAATGTGCATTAT
TGCTTGGTGGCCAGGATGGAATTAGACTGTGATGAGTCACTGTCCCATAAGGACGTG
AGTTTCGCTTGGTACTTCACGTGTGTCTTTAGTCATCATTTTTTCGAAGTGCCTGCCC
AGGTCGGGAGAGCGCATGCTTGCAATTCTAACACTGAAGTGTTGGATGATGTCGGAT
CCGATTCGAGAGACCGAGGCTGCGGGTTCTTGGTCGATGTAAATCATTGAAACCTCA
CCTATTAAAAGAAAGAAAAGTATCTAAGGCCATTTCAAGGACATTTGACTCATCCGCTT
GCGTTCATAGTCTCTTACAGTGCTCTATACGTGGCGGTGCAAACTAAAACTCAGCCC
GTTCCATTCCTTTGTATTGTTCAGTGGCTAGTCTACTTACACCTTGGCCTCTGATTTAG
CCAGCACTGATCTCAAGCGGTTCTCTAAGCCTACTGGGTATAAGTGGTGACTTTGGC
CAGAGTCATAGTGGATCACAAATCACTGGTGAAGAGGTAGAATCCTACCTTCTTCCAA
AATCTACCCCATGACTATTGCTGGGGTTGCATTTTGATTTCAATGAATATTTTGGATGC
CAACGACACGTCTGATAGTGTGCTTTGCTAGTGTTTGAATTTAAAACCGAAGTGATTG
TTTTCAAAATGTATTTACGATTTGCTTACTTGTTGAATTCATTTTAATTACCTTTAGTGAA
TTGTTACTTTGGAGTCCTTAAAGTTTTCAATAATTTTTTTGGCAGATGATACTCAAATTA
CTTGGCACTTAAATGTACTTTCTTTCAAACTCATCCACCGAGCTACTCTTCAAATTTTT
AAGTCTTATAACACAGATACTGTTAATGTAAAGTGAACATTATGACTGGATGTCAGGAG
TATTTGAGGTTCTATACCAGTTCAGGCTTTGCTTTTGTTGCTATTGTTGATGCTATATTG
ACTAATGGTTTTACTTGTCAGCAAGAGCCTTGAATTGTAATGCTCTGTGTCCTCTATCA
GACTTACTGTTATAATAGTAATATTAAGGCCTACATTTCAACTTTCTGTGTGTTCTTGCC
TTTATGGCATCTAGATTCTCCTCAAGACTCAGCAAATAGTGCTGCTGCTATTGCTGCC
CCAGCCCCAGGCCCAGCCCCAGCCCCTGCCCCAGCCCCAGCCCCAGCCCCTGCC
CCAGCCCCAGCCCCTGCCCCTGCCCCAGCCCCTGCCCCAGCCCCAGCCCCAGCC
CCTACCCCTGCCCCTGCCCCTGCCCCACCCAACCAACCCAATCCAGTCCAGCCCTG
CCCCAGCCCAGTCCTAGCCCCAGGCCCAGATACTTTCAGACCTATCCCAAGCCCACT
TCTACTTAGAGAAATTCGAATCTTCATTGATTCAGTGCTAAAATGCAGTGTCCATCACT
CAGCCTATAAGACTGAGACAGCCCATCTATACCCCCTCCATACTGACTTCTAGAGTCA
TGGAATTTCACTTAATGCATAGAATCGTATTGCTAAAATGCAGTGCCCATCACTCAGCC
TATAAGACTGAGATAGCCCATCTATACCCCCTCCATACTGACTTACAGAGTCATGGAGT
TTCACTTAATGCATGCAGTCCTATTGCTAAAATGCAGTGCCCATAACTCAGCCTATAAG
ACTGAGATAGCCCATTTATACCCCATACCCCCTCCATACTGACTTCTAGGGTCATGGA
ATTTCACTTAATACATAGAATCGTATTGCTAAAATGCAGTGTCCATCACTCAGTCTATAA
GACTGAGATATCCCTATGTATACCCCATACTCCCTCCATACTGACTTCCAGAGTCATAG
AATTTCACTTTGCATACGGTCCTATTGCTAAAATGCAGTGTCCATCACTCAGTCTATAA
GACTGAGATATCCCTATGTATACCCCATACTCCCTCCATACTGACTTCCAGAGTCATAG
AATTTCACTTTGCATACGGTCCTATTGCTAAAATGCAGTGCCCATCACTCAGCCTATAA
GACTGAGATAGCCCATCTATACCCCCTCCATACTGACTTCCAGAGTCATGGAATTTCA
CTTAATGCATGCAGTCCTATTGCTAAAATGCAGTGCCCATCACTCAGCCTATAAGACT
GAGATAGCCCATCTATACCCCATACCCCCTCCATACTGACTTCCAGAGTCATGGAATT

TCACTTAATGCATGCAGTCCTATTGCTAAAATGCAGTGCCCATCACTCAGCCTATAAGA
CTGAGATAGCCCATCTATACCCACTCCATACTGACTTCCAGAGTCATGGAATTTCACTT
AATGCATGCAGTCCTATTGCTAAAATGCAGTGCCCATCACTCAGCCTATAAGACTGAG
ATAGCCCATCTATACCCACTCCATACTGACTTCCAGAGTCATGGAGTTTCACTTAATGC
ATGCAGTCCTATTGCTAAAATGCAGTGCCCATAACTCAGCCTATAAGACTGAGATAGC
CCATTTATACCCCATACCCCCTCCATACTGACTTCTAGGGTCATGGAATTTCACTTAAT
GCATAGAATCGTATTGCTAAAATGCAGTGTCCATTACTCAGCCTATAAGACTGAGATAT
CCCTATGTATACCCCATACCCCCTCCATACTGACTTCCAGAGACATAGAATTTCACTTT
GCATACGGTCCTATTGCTAAAATGCAGTGCCCATCACTCAGCCTATAAGACTGAGATA
TCCCTATCTATACCCTCTACCCCCTCCATACTGACTTCCAGAGTCATGGAATTTCACAT
AATGTATAGATTTCTATTGCTAAAATGCAGTGCCCATAACTCAGCCTATAAGACTGAGA
TAGCCCATCTATACCCCCTCCATACTGAGTTCCAGAGTCATGGAATTTCACTTAATGCA
TAGAATCGTATTGCTAAAATGCAGTGCCCATCACTCAGCCTATAAGACTGAGCCCATC
TATACCCCATACCCCCTCCATACTGACTTCCAGAGTCATGGAATTTCACTTTGCATACA
GTCCTACTTTACTTGTCCATGGACAAGTAAACAAAGAACTCTTGTCCTTCATGTTAATC
AAGATACACCAATCAAACAAGAGTTTTATATCAGAGACTTGCCATGGAGGTATCATCTC
TCAAGTCTCCTTTCCTTTAAGGAAAGAAAACCATTCTGTCATTGCTGTAGTAGTCACA
GTCCCAAGTTTCTAAGCAGTGTTCAGTCGTCTTTTCTCATGTATTACCTTGAGTACTGA
ATAATTCTGTCAGAAATATTTTGTCCATTGGATTAGACTTTAGCTAGTCCAGCCCTGTG
TGCATTTAGCAAAGGGGCAAACACAGGTCTGTTATCAGACAGTTAAAGTGCTCAGTC
CCAATTTTCAAGGCATTGGCCATTAAAGGGGGTAGAATACTATATACTGTTGGCATGCT
GTCATGGGTGCTATCGCCCCAGGTCACATCTTTCTAACTGATGGAGATACATTTATTT
GCTCATGATATTGTATACTAGTCTCACATGCTTTCTTATTTCAGCCAAAAACCTCTGCA
CTGGAACATTTTATGTGGATAATCCTGACTAGGAATTGAGTCTTTTCTCAAGGTCCTAA
TACTACCCTTGCTTTATGTAAAGAGGGTGCTGATTACTTAATGCCTCTTACACAATTGT
GCAAAATTGCAGTTGTTCAAGTCCCCTTCTGTTAGTAACCAAGATCCCATACCCTCAT
ACCCTAATGGGTGACAATCAAGGGTGCCAACCAATGAGACCACTTCTCTGTTCTGGT
CTTTCTGCTGTGCTGGGGAATCAAACCTTGAGTCTTGTGTACGCTAGTAAAGCACTG
TCATAGAGCTACAGCCCCACCGTGTGGTGGTTTGAGAGAACAGCCTCTTATGTAGCC
TGGGCTGGGCGGGACTTACAGGCATTGCCACCTGTAATGTAAACATATTTGTGCCTG
TTGTGTGCACAGCTGCATTTGTCCCTCTTCCTAAGCATTGGATAAAGAAACCAAACTA
AGTCAAGTCATTTTGTTGGTAATCAAGAAGACCTTTGATCTGTCCTGTTTTTAACTTCC
AGGCTGGCCTGGAACTTAGCATATAACCCAGGCTAGCCTTGAGCTCAGGATCTAGCC
TGCGTTTAACAAGTGTTGGCATATCTGGTTCCTACCACTATGCCCTGCATGCAGTCTT
TCATATTGTGAATGTGCATATGTCATTTCACTGTAGTAATCTGCATCTGGTGAAGACTT
ATTTGTATTGCAGCAGTATTTAAGATCCTTAACATAGTAAATGTGCACAGTGTTAACTCT
ATTGTACATATTCTCATGTCCACAGTTGTGCCTTTTAGATCAGGACTCCTGTACTTAGC
AAAGCAAAGAGGCTCACTAATATAAAGCTTCTTTCATGAGACTATAGATTGAAACGATT
CCAATACGGTCAATGGTCCTTCAAGGTAAGACTTCTGTCTCTGATCATTCATATCCTCT
TTGCTTTATGGAATTATGTATGTGCTGTGCACTTGAAACCCCTTCCTCAAACTATTTAT
GTACATACTGGCAATTTTAGTAGGATCAATTTTACTCTTAACTTTGAAGTACAGAAGTG
GTGTTGACCTATAAGGTCCCATTTTGTGGCTTGCTAATAATAATGACTGATTGTAGTAG
GCCTTTTCTGTTCACTACAGAAGGAAACCTGAACAGCGTAAAACTGTAATGGCCATAA
ACATGTACCTTGCATATTAGTATGCATTTACTGCACACATCTCATTCCATTTGGATACGA

TCCTACTCTCAAACCCTTTTGCAGTACAGCAAGGGTCACTAATCTTTTGGCTTCTTCA
TCTTCCTGGACACTGGATAAGGCTGTCCCCTCCTTTCCACTCTTTAATTTCCAGGACT
ATTACTTTAAAGACTTAATATTTGCATAAAGGATGGGGTTTTTAATTGATAACATGTCCC
TTGAACATTAATGTATATAACAGGGACATGATCCATTCATTTTAATAAAAATACTTGGCC
AGTTAATGTGTAAAATTACACTTATCCACAACCTTATTACTTTTCGGACCATTGTATCTC
TTGCACTCCTGCAAGGGATACCGTTTATCTCCCAAGGTCCCTGCTAGTGGACCATTAA
TATACAGTGAATCTTCCTTTGTCTTTGCCAGTAAACAAAGGCCATACTCCTTCGCCTTT
CATTTGCACTATATCAGGATATGCTGATCAACAAGGCCGCATTCTTTTGGACTGTTATC
ATATATTAAATGTATGCGTATGCACTGCCACCTGCTCTGTGCACTTGAAAGGATCCCA
CTCACTTCCTTAGCACCTTCAGCAGGAAGTGATAATAAGCTCAAGACTTTCATTTGGA
AAGTTCACATGTCTAAGCACTTCTCTAAGAACTACTGTACCCTCTTCTCCGCTTTAAA
GCAGAAAGAGGGTTGTACGAAGTGCTCTTCATTTGGACTTAAGTGCATTAATGCAGTT
AGTTGTCCATCATTACCTTTGGAGTTGGATTTTACATCCTTGTACTCTTTTGACACCAG
AGGCATATTAATTATTTCTGAGCACTTCTCTTGTCAATATTAATCTGTACCCTTACACATA
TGACCTGTGCGGCAGCAAAGGTTCTGAAATGCCTACCTTTTGACTGGGGCTGCTGA
GTGGTAGTAACTATTAGTAACCTCAGCATTTGGATGATTACTATGCAAAAATGTCAAGG
ACCTGTGTGCTCTCTTTGCATACCATCAAGGCTACTGAGTCCCAGAATTAATTGCTAA
GTTATGCGTATTTATAACTATGAATGTCTGGAATATTTTGTCCCCTTTACATTATTGCAG
AGGTTGCTGAGCCCCCGAAACTACCCGGTACTGTCAATGAGCACAGGGGCTCTGAC
GAATGACCTGCTCTCTTCCTTAAACTGATTTTGGGACTCTTAATAGGCACAATGGCAG
TTCTGGATGGTTTATTTTCTACTCCAACTTGAGCAAATCCCCTGCTAGTTTCCCAATGA
TATAATAAAGTACAGCAGTATGTACACCCAACAATGACCCGGATTTCGACCCTTTTTGC
ATTGCTTTAATATATACAATCCTAAATAGTCACAATCTCACACTTTATAGTGTTCCTTTTG
CCCGGCCTCTAGTTTGTCCATTGACCACTTTTCTGAATCACTAATTCTCACAAACCCA
TCATTAAGGAAGAGTTTGTGCCCTTTCTCAATTCCATCATGCCATCCCTTTTGCCTCTT
TGTTTGAACAGTATTGACTGGGCAAAGCCCTTCTCTTGACTTAAAGTCAACAACACCA
GTTTACTCACTTCATATGGCTACAGTGTCTCAGTTGCCTTCTCCTTGCTCCCACTGAA
CAGAGACACCTCGAATTCTTACATTATTCTGGGTAATGTTAATTACCCCAAACACCCTA
TGTGTCATTAATAAATTTTGGTGTATTTATACACTGAATAGCAAAAGCAGGCCAAAACTA
GGTGGATGAGCCTTCAATCTTTAACTTGCACTTCTAAATTATTCCAATTCCAACTGCTG
GCACATTCTAGGGCCAGGAACCATTCTTGCCTACCTTTATTAATGCTTTATTGTGCAAA
ATATTGCAGGCAAGTAGCTCAGGGAGTTGGATTGCCACCTTTTACTTGGGGCTTTCC
TTTACAGTATGAACTGAAAATTGTCTTCCTGAGAAGGAAGCTTAGCACTTTTCTTTCC
GTTCTTCCTCCAGGAAGGAGCCAACTGTCTGCTTAAGAAACTTTAAGCCCGATTTTG
TATATTGCTACTGTACAGGACCAACTGCCAGAAAAGTTATTGATAATTTTATTCCTTAAG
AAAGGCATTTGGATTGCAAGGTGGATTGACTGTGAGATCATTAGCTTTTGTGAAGTAA
AAATAGCCATTTGTGTCATGTTTCTGAAGACTAAGCAGTGTCTCAGTGTACTGAGGGT
GATGAGTCTGTGGAAAGATCAGTGCAACTATTGCAGAATGTTTAAGACAAGTATCTTT
GCTTGGTCTTTACTACAAGTTTAACAAAACGAAAAAGTCAATCTTTGTGTGGCCTTTA
GTATGATTAACTTTTTGGAAGATGACCTAAGCCTTCTAATCATTATATTTTGTCTGACAT
TGGTCACCAGTCCTTGCTTATTTTTAAAAGGTGACTGGATGGATTAAATTTGAGAACAT
GTCAAGTCGCCTTTGAAAATTATATAGGCCATCACATTTAATTAATTCATTCTATCCACC
ATTAAACTCTGGCAATAATTTGAAGTAGCTTGAAAATTCCTAAAGTGGGAATTTATTTTA
GAGATGATAGAACCTGTTTCCCCACTTTACATTTTAAAATATGTCTGCCAGGATCTAAT

CATTCCTTTAAACGTACACTTCAAAGAGAGATTTTCCTAGTAAGAAAAGAGCTTTCTCT
AGTGTGAAGGGTGCTTTGTAGCCGCCGAGTACTTAGGTCTTTTTTGGGAGCTATTGT
GTATGAGTGTATGTATGTGTGTGTGTACATGCATGTTGCTGCGCGCAGTCATTCATTC
ACATGGTGCTCAGACAACAATGGGAGCTGGTTCGTCTATCTTGTGGGTCCTGGAGAT
CAAAGTGAGATCATCAGGCTTGGCAGCAAGTGCCTTTACCCTCCGCGTGCCATCTTG
CCATCCCGCTGCTGAGTGTTTGATATGACATTGCTGATGAAAATAATCATCACAACAG
CAGTTCTCCCAGCATTACTGAGAAATGATACTATTTTTCTGAGGAGGATGTTCAAGTAA
CTCATCCAGTGCAGGATCCTGCTTGAACTACTGCTCCTCCGTTACATCAGACTCTGG
CTGTTTAGACTACAGGATGAATTTGGAGTCTGTTTTGTGCTCCTGCCTCAAGAAGAAG
GATTGCCTGGATTTAGAGGAGTGAAGAGTGCTGGAGAGAGCCCAAAGGGACAAACA
ATCCCTATGTGAGACTCAAGGACTGCCAGCAGCCTATACAGCTACATTACATCTCAGC
AGAACTTCTCTTCAAGTCCTCGCTACTCTGAACAAAAAGCTTACAGGCCACATGGAG
AAAAAAAGATCTCCCCCCAGAATTGTGGGCTTGCTGCTTTGCAGTGCTGGCGACCTA
TTCCCTTTGACGATCCCTAGGTGGAGATGGGGCATGAGGATCCTCCAGGGGAATAG
CTCACCACCACTGGGCAACAGGCCTAGCCCAGATTTCAGTGAGACGCTTTCCTGAA
CCCAGCAAGGAAGACAAAGGCTCAAAGAATGCCACCCTACATCAAAGTAGGAGAAAA
GCTGCTGCAATAGTGGCACTGACCTTCGAGGAAGCCATTCTGCTCTATTTGGTTCTC
TCTCCAGAAGCTAGGAAAGCTTTGCCAGCTGTTTACATACTTCAAGATGCACTGCTAC
CCTACTCATGCCATATAATACACAATGCCATCTACCAAATATTACCCTTCCCCAAAGCA
GCACAGAAAACTGGGTCTTCAGCGTGATCAAGCAATGTGAACACACAAAAGGAAGG
CAGCTTTATAAATGACCCGAGGATCAACATGCCTGACTGCAGCATCTTAAAAGCAATA
GAATGAGTGTGTATTGTGGGTGTGTCTATTTCTTGTTTTATGTATCTATTTTTTCCTTGG
TCTGTGTGTCTAATTCTTTGTTACATCTATTTCTTCCTTGCTTTGTGTGTCTATTTCTTC
CTTGCTTTGTGTGTCTATTTCTTCCTTGCATTATGTCTAATTCTTTGTTATATCTATTTCT
TCCTTGCTTTGTGTCTATTTCTTCCTTGCAGTTGTGTCTAATTCTTTGTTACATCTATTT
CTTCCTTGCTTTGTGTGTCTATTTCTTCCTTGCATTGTGTCTAATTCTTTGTTATATCTAT
TTCTTCCTTGCTTTGTGTGTCTGTCTTCCTTGCTTTGTGTCTATTTCTTCCTTGCAGTT
GTGTCTAATTCTTTGTTACATCTATTTCTTCCTTGCTTTTGTGTGTCTTTCTTTCTTGCT
TTTGTGTGTCTATTTCTTCCTTGCAGTTGTGTCTAATTCTTTGTTACATCTATTTCTTCC
TTGCTTTTGTGTGTCTATTTCTTCCTTGCATTGTGTCTAATTCTTTGGTATATATATTTCT
TCATTGCTTTGTGTGTCTATGTCTCCTTGTGTTGTCTAATTCGTTGTTGCATCTATTTCT
TCCTTGCTTTGTGTGTCTATTTCTTCCTTGCTTTGTGTGTCTATGTCTTCCTTGCTTTG
TGTGTCTATGTCTTCCTTGTTTTGTGTATCTACTTCTTCCTTGTGTGTCTAATTCTTTGT
TACATCTATTTCTTCCTTCCTTTGCATGTCTCCTTCTTTCCTTTGTGTGTCTTTTCTGTC
TGCAGTGTGTCTTACCTATTCCCATGTTTCTCCTGCATGTTCTTTCTTGCAGAGCTTT
GAGCTTTGTTTCACTTTCTCTGGTGCCTGTGTGGTCTGCTTTGTCTTCACTAGCTATG
GCTCTCTGTTTTATCTATCTGGTTGCTATTTCTCTTAGCTTTTCTTTCACTCCTGCCTTT
CGTGACTCCCCTTTGGGTCACATGTTGCATGCATCCCTCTCTTTTTCTTGTGCTCACC
CCACTTGTTCTTTGTTCAAGTTCTCTTTGTCAGTCCATTTCAGTTTTCTTTCTGCTGCT
TCTATCCTTAGTGAATTCTTGTTTACATTTCTTCCCTGCCTTTCTTGGGCCACTTTCTC
TGTTTTCTTTTGTATTTGTGTCTCTTTGCTATTGGTGGATTTCTTATCTCAGCATCATTC
TGTTGCTTTGTGTTTGCTTGTGTTTCTATCTTCTACTTTCCTCCTTTCTGTTCACTTTG
AGCATTTCATCTCTTTACAAGTCTGTGTCTCTCTTGTATTCTAAAGTAATCCTTTCTTGG
ATGTTTCTTTGTATGTACATGTGCGTGTGTGCATGTGTGTTATGTGTGTCATGTGTGAG

AGGAGCTTCATAGCCCCTTCCCAATAGGTCCAGAATGTCACCCGTGGAGCCGTTCCT
CACACCAGACTGCCCTGAGAAATAATCTAAGACAAAATACATCATTCCGTCCGGTCAG
GATTCAAGTGGCTCTGAAGTGAACGCCCAAGTAGAAGACAGAAGTTTTGCGACTTGA
GATTTAAAAGGACCAAAATACACAGATGGCCCGTCTTGAGCTGGCTGGACAGAATGC
TGACAACCCAAAGAAGAGGAACTGTTTCTACAGGACACCTGTGACTTCCAAGAGCG
GGGAACTACGTATGTCATAAGACACAAAACCTGAGCTAAGTCCAAGCATAAGACCTAA
GGACCCAATCCTATATGGACAGAATATTTAAGAGATAAAGGCCTATGGCCCAGAACTC
TGGAAGGATATTTCTATCCTTCTATCCCCAAGACCAAGAAGGGAAATTCGAAGATGAG
ACCTGCCCCCCAACCCCAGCATCCCTTTCCATTTCTTATATTTCTATTTAAGCTGTCTT
CACTTGAGATGTAATTTTTCATTGTTGCCATTGCCCATAAAGGAATACGTTTTTAGCTG
GATAGTATTGTGCAAGGGTCTGTTTTAAACTGGGTCTTAGCCATTTGTTAAATTGTTGA
TGTTTTACAACTTCCATTTCTCTTCACATCTGCTCCACTTGAGACGGAACTAAATCCA
GCCAGTGTATATAGCCTGACTATTGAAACTTCCCTAGGAATAAGCATGCATACAGATAT
GCATACTGCCATCCTCCCTACCTCAGAAGCCCTAGGCTGACAAGAAAAGGAAAGCAT
CAGGTTGTTAGGGGGAAAACAATGTCAGGCTATCTAGAGAAAATATAAAGAGTTGTTC
CAGACCAATGAGAAGAATTAGACAAGCAATATGCAGATGTGCCAACCCTCTGAGAAG
CACCAGCCAGTGTCACCTTCTTTCTTTGGGCTTAGGTGAGCAGGGTATGGTTTTCTA
ATAATGGTTTGGGGACAAAATGAGGTCTGAACTCCCTGCTCATAGTAGTGGCCGAGT
AATTTGGTGCATTTCACCAAAGGAACTCCTGGGTCTAATACCTACCTTTAAAATTAATG
ATGAGAGACTCTAAGGACTACTTAACGGGCTTAATCTTTTTCGTGCCTTCCTCTTCCT
CTGTAAGAGGGAAGTTAAATGACACAGGATGAAAAAGTAACATGCTCATAGCACATTG
GCAATTATACATGGTTATTATCTGAAAGTGTAGAGCTTTTCCTATAAGGCATCAGACTAA
GTACCTGAAGCTTTGTGGGTTCATGGTCTTAGTTGCATATTCCTTAGTTGCAAATCCTT
TTCAAAAGGTAAGAAAAAGGCACACTGGTCTATTGCCTGTACTTGATCAAGCCCTGAT
ATGAATGCCAGGGAATGTCTGAGTAACATTAATTTCCTTCCCTGCATATTTTTTGTGCT
GAATACTAAGGCTGTGATGCTTCACTGTGGTCACCCCCAGGTAACAAGATATTACCAG
GTAACCAGGAAACGTATGAATACGTAAACCATGAAGCCTACTGTAACTTCCAAGTCAG
TGCTGAGTATGTATTACATAGTAGCTGAAGTCTACGCCTCTGTGTGCTATAGGCACAA
AGATTGCTCTAGGAATAACATGCTTTGTAAAAACAAATATATGAACATAACGGGGCTTG
AATGAATAACAGTCCATATACTTAAGGCCAGTGTGTTTCTTCTGCTTTGGTGAGGCTC
AGTAAGTTATATTATACCAGGTAGCAGAAGAGAAAACACATGGAAACTGATTTTAAACT
ACAAACTAGGTCACTAATGCAGGTGATTGATTACCCTATTCTGATCACCTTCTAATTTC
TGAATACCCATGTTCAGCACTGGGAATAACAAAGGGGGACATTACCACAGAACTAGA
ATTTACAAAAGAATGCATTAAATAAAGCATTATACAGCTATCAATTGTTCCATGTGTGCA
AATGAATGACTACTAACTACCTCTGATGTATCCGATATTGTTTTGGGTACATGAAATATT
CATGAGTAACTGCCATGAAATAAGAATGTTTGCATTCCATACTATTCATAAGGAATGAG
CCAATGCTTAATTTAATCAGTCAAAACTTGAGTGATAAGGGCATGTTAATACAAGAACA
TTTGCCCAGGTCACATTATGGTTGTGGGTACTTTCTTAACTATAAAGCAGTTCAGTAGT
ATAAGACAAGACAAATTTTCTATAGAAATAAAGCTGCCTATAAAATAGGCATAGTCTCTA
CAAAATTTTCATTGTACTTTTTAGCCCATAATGGGAAGAGTACAGTTAACAAGCTGGGT
GTGGTAGCATGTGCTCTGAGCTGAAGCAACAGGACCACTTGAGCCCAGAAATTGGA
GGCTAGCCTGGGAAGACCATAAGGTCAATCTCAAACCTGGAGGCTAAATATTGTCTC
CCATGTGTATATTCTCTTTCATGGGTACTGGAGAGATACACAGACGTACATTTCAGTGT
GTCCACACTTGAGAATAATATGTACGTTGGCATTTTATGAACTCGGAGGTACCATATAA

ATGTAACAATTCATTTTCTTACTTGGTATCAATTTCCAGGCTTTTAAAATTCTGCCACAT
TTATTATACTGTGAAAATAAAGTAAATAAGTAACTGTGAACCACTGAATATATGAAGCAT
TCAATACTTGATGAGTACATACTGAATGGCAGTCATTTATTACAAAACAGTGCCCTTGC
TAGGCACTGGGATGCAAAGAGCATTCTCATTGTCCTGTGTATCTAAAGAAATTATGCAT
GAGATTAATTTATAATTTGTAAACTGCCATATATATGTGTATATATGCAATATTTGCCTGGT
GTGCAATGACTTTGCTTTTATCCCAGGCATGCACAACAGATCTGTGTGGAGCTTTGT
GAAGTCTACAGTTCTATAAAGCCGGGACCTAACTGTTGGCTTTATCAGTGAACAGTGA
TTACTTTCTAAGTTTCATAATGGCTGAAACTTAATCATAATGCTTATCACCTAACACCAC
CTAATAATAATTTTACCATGCTATGTGTTGAGCGAACACATAGATTGCTTTCTAGCATTA
TGTAGCACTTATAGGAGTGAAATCTAGACCAAAACTTCAATTCACTTCAATGAGGAAAT
GAAAACAGAAAAAAAAAAATGGATTTGTGCAAGGCAGTGTGCTAAATGTTACACTGAGT
GGACTATGCTGTCTAGGATACTTCCCAGCTGGCTTGACTGAGGAGGTGGAAAAGGTT
TTATTAATGACAGGAACTTTTTCCATCCAGTTTCTTAAATGTTTGTTGAATGCTGCTGC
CAGAGATGAATTACAAACACCTTGCCAGTAAAGGAGTTTTATAGGGCCAGAGTGAGAT
AATCCCAGAGCATGGGTATCAGGGAACAAAACGGGAAGAGGCCAGAGCATCTGATG
GCATGTACTCAGTGTGGCCCAGAACCTCTCGAACTAGATGTACTGGCTGGAGGGAC
CAAGCATGCAGAACACAACACCTAATGAAACATTGTATATAAAATATGCTAACCTAGGT
CCTAAAACTAAAATGTGAGGTGGACCTAGTGTAGATCACTGATCATAGGAGACATGGT
CTCATAAAGCCCAGGCTGGTTCTAATTGGTGACTGTCACAGCTTCTCAAGTGCTGAG
ATTACAGATGTGCTTAACCCATGCCCAGCCTGAAGAATATATCTGATTACTGAGTGAAT
AATATTTTTAAAGAATTATATATTTTATGTATATGAGTACGCTGTTGCTGTCTTCAGACAC
ACCAGAAGAGGGCACCACATCACATTACAGATGGTTGTGAGCCCCCATGTGGTTGTT
GGGATTTGAACTCAGGACCTTCGGAAGAGCAGTCAGACTCTTAACCACTGAGTCATC
TCTCCAGCCTTCTGAGTAAATATTTTAACTATAATGGCTGTTTGCGAAACCCAACCAAG
GCCAAGATTCCTTCAACATAAACTGGAGACTTCCTAGCTAAGGAAGCTCCAAAAGTC
ATTTTCTCATTGGCCTAGCTTGAAGCCAGGACAGACTTAAAGTCTGTCCTTTAATTCAT
TACCCATTTTCCTTTTCTTACTGTTGAAGTGTTTCAAAGGAGAATCAAGATGAATCGAT
AATTCTAAACGTATTTGTTCATTGCCTGGCTCAGCGTCATGTGAGCAAGAAGAATATAC
TATCACACTCATACTTTTAACTTAAGTGTGATGAAAGTGCAGTTCTAAGTACTAAAATTT
CTAAGTACTGAAAAGAACAAAGACATTTAAAGGATGCAACCCAAAGTGTACTTTACCT
CAGTAGTTTCTGAGGGGACTGCAGTCACACCTTGAGACTACAGCTCTCACTTTAGCT
GGGAAAAACATCAAGGTGTAGAGGAGGCAAGTTAAATAAAAAGTTGCTCCCCTCCTC
ATGGGCATGCTTGGTAGAGTGGAAATAATAAAAGAGGTTCTCTATTTCCTCGGTTCCA
CACATTGCAGAAGATGCTACTGGATGCTAAGTGCAACACATTTGTTCCAAAAGGGCA
CTCAGTGTGACTTACAGATGCCCCGGAAAGCAGAGGGATGCTCTTTATTAAACAGAA
ATATTAGCTCAAACGTTTTCTAGACTGAAGAACACTTTCCTCATTTCCCACAGTTTGCC
TCAGAGGTTGAATACAGGAAGGTTATTATTCATTCATTTGCTTTATTGGTTCGCCTGTT
CTACAAGGATTTGCATGTCTCTTAGGCCTTCACTTGGCTCCTGAGACATGGAAAAAG
GAAACATAGACATAGGGAAGTGCTGGATGGGGGGGGGGGTCTCTTTTCTGGGTAG
TGGCACGACTTAGTCCTTAGTCCCCAAGTAATATGCAATGTGAGTCCTCATCCTCATG
TCTTCTCCGGCCACTGCAATGAGTGGGAAGCTGGGCTTTGTAGCAAGCCTGACCCT
AAAGTTACAGAAGCCCTCCACGCTAAGAAACTCAATTTTCTAGGCCATTTTAGCTATG
ACTGTGACCACTACTGGTCAGGAGGGATGACAGCCATCTAAGTTCCACAATCTTAGG
CTACTTTGCATTATCCTGGGGCAAACAAGCCATTTTTGAGCTGCAGCAGGCTTTGAAA

TACATTGACCAATTTTGCCTGTGTTCGTTAAACCTTTTACCTTTTTACATGCTAATGCTC
ACAGTAATTTAGAAATGTTCTCCTTACTATAATATACTCAAGGTGGCTTGCTATGGTAAA
ATAATGCCAGTGGATGAAAATAACATTAATGTTTAACATTCTTGCATAAAATTTAAGAATA
ATAAAATTGACAACAATCAGAAAACTGGAGGAACGAAAGACCAAATTGAAAGAACTTG
AAAAAGATTAAAAATGCCTGTGCTTTGACCCTTTCCATTTTTCTTTCACTCACAGAGG
GTGGGACAGGAGGCCGAGTGAAGGAAAGGGTCCAGCCTGTCTATCTGGAATCTAAG
TTGGGACTTTAATGCAGTTCCACAAAATTGGTATTAATTCGCTAAATGTTTCTGAAAAT
GTATTTTCATCTAAATGGCTATCAGCTAAGCCTTGAGTCAAATGGGAATGAAACAGATT
AAGTCAATGTGATCTCTTTATCCAAGTTGCCTTAGAGCTGAAGTCACAATTTGCTGTAA
GGAAGCTTATTCATTGTAGCATACGCATACTTTCAAAGTATCTAGACTTTACTTAGTAAC
CCAATCAGGACATTCAGGCAAAAGAAAAGGAACAGAGAAGATGGAGCCAGGTTGAA
GAGGTCTGGGAGTTCAAACAAATTTTTTTCATTTTCATTAAAACTCAATTGGGCATCAA
AAGTGTTACTAATATTAGCTTTTAATTAGTGGAAATTGGCTGGATTCAGTAATATCCCTT
TGTATGGGTAGGAATGGGCTTACATTTCTGGAATTTGCAAAGGAAAAAATAACTGAAA
GCCTTCCTTTCACAGTTACTGCCATCAATATTGCTACCAATTAAGCACATCCTACCATC
ATCTGCTTTGATCACATAAATGAACTGTGTACCAATCTGTTGTTGAAAGACTGGAGTC
ATCTTCCCACCAACTGTGAAAAAACACATGGAAAACACCTGGACTTTGTGAACGGAT
GCGGAATACAGAACTTCTGTTGACTCTTGGGTGTTTTGAAGACTTGAAAAAAAAAACT
GTTGCTTACCAACATGTCACAATGAGTCCGTGTGTGGGTGGGTGGATGGGTGGGTG
GGTGGGTGGGTGGGTGGTTGAGTGGGTGGGGTAGTTTGCTGTTAAATAAAATGCTTT
GTTTTGAA
